# Orientationally Resolved Electron-Spin Relaxation and Coherence in Fullerene-Encapsulated Rare-Earth Dimers with a Single-Electron Metal–Metal Bond

**DOI:** 10.1021/jacs.5c22495

**Published:** 2026-02-10

**Authors:** Michal Zalibera, Lukas Spree, Fupin Liu, Marco Rosenkranz, Leonid Rapatskiy, Alexander Schnegg, Alexey A. Popov

**Affiliations:** † Institute of Physical Chemistry and Chemical Physics, Slovak University of Technology, 81237 Bratislava, Slovakia; ‡ Leibniz Institute for Solid State and Materials Research (IFW Dresden), 01069 Dresden, Germany; § Center for Quantum Nanoscience, Institute for Basic Science (IBS), 03760 Seoul, Republic of Korea; ∥ School of Chemistry and Materials Science, 12534 Nanjing Normal University, Nanjing 210023, China; ⊥ Max Planck Institute for Chemical Energy Conversion, 45470 Mülheim (Ruhr), Germany

## Abstract

Rare-earth dimers with single-electron metal–metal bonds feature strong hyperfine and exchange interactions between metal atoms and an unpaired valence electron. Their stabilization inside carbon cages opens the possibility for applications in quantum information processing and sensing but requires investigation of their unusual spin states and dynamics. Herein, we present a systematic EPR study of dimetallofullerenes M_2_@C_80_(CH_2_Ph) with M_2_^5+^ dimers, whose composition varied from Y_2_ (*S* = 1/2) through YGd (*S* = 4) to Gd_2_ (*S* = 15/2). High-field W-band EPR spectroscopy facilitated the analysis of different molecular orientations and *T*_1_ anisotropy and allowed resolution of individual |*m*_S_⟩ → |*m*_S_ + 1⟩ transitions in high-spin Gd dimetallofullerenes. For Y_2_@C_80_(CH_2_Ph), we demonstrated that fullerene isomerism strongly influences the molecular and spin dynamics of metal dimers. The pronounced difference in spin-lattice relaxation times *T*_1_ was found for the *I_h_* and *D*_5*h*_ cage isomers and rationalized by Raman spectroscopic study and calculations of spin-phonon couplings. Lateral metal modes, which play the main role in the spin-lattice relaxation of endohedral metallofullerenes, were found at higher frequencies in the *D*_5*h*_ isomer, in line with its longer *T*_1_. The orientational dependence of spin decoherence was ascribed to the rotational motion of the Y_2_@C_80_(CH_2_Ph) molecules in glassy toluene. Substitution of Y with Gd accelerated spin-lattice relaxation and decoherence. A similar *T*_1_ and phase-memory relaxation time (*T*_m_) of YGd@C_80_(CH_2_Ph) and Gd_2_@C_80_(CH_2_Ph) proved that the effect does not scale with the number of Gd atoms. While the Rabi frequency is constant across the whole spectrum in Y_2_@C_80_(CH_2_Ph), YGd@C_80_(CH_2_Ph) and Gd_2_@C_80_(CH_2_Ph) exhibit variations in the Rabi frequencies for their |*m*_S_⟩ → |*m*_S_ + 1⟩ transitions.

## Graphical abstract

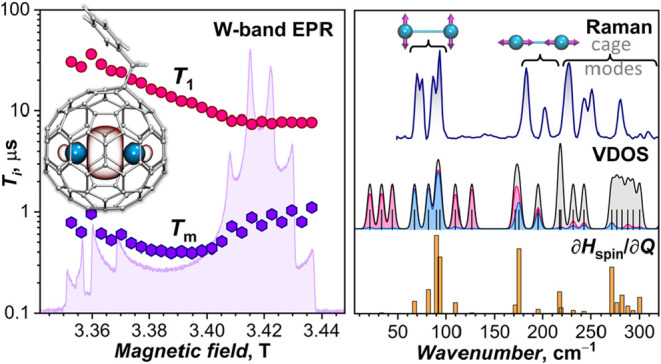

## Introduction

Spin in a molecule is one of the qubit platforms intensely investigated for use in quantum information processing and sensing.^1–11^ As spin-lattice and spin-spin relaxation times set fundamental limits for qubit performance, strategies to increase these figures of merits are being actively pursued,^12–25^ alongside further steps necessary to control and scale qubit operations. The ability of fullerenes to support various radical states via endohedral encapsulation or delocalization over their extended π-system made them viable qubit candidates,^26–32^ N@C_60_ being the most famous among them.^33–41^

Aside from the nitrogen atom, carbon cages are able to stabilize ample variety of endohedral species, including endohedral metallofullerenes (EMFs) with peculiar spin properties.^42–49^ One of the unusual entities brought to light by stabilization inside the fullerene cage is the single-electron bond between rare-earth metals.^50^ It was first obtained in 2008 in the form of azafullerenes M_2_@C_79_N (M = Y, Tb,^51^ later also Gd^52^ and Dy^53^). The stable closed-shell charge state of the azafullerene cage is C_79_N^5–^, suggesting that the endohedral dimer has the charge of M_2_^5+^ and can be formally described as [M^3+^–e–M^3+^]. The oxidation state of the metal atoms is thus +2.5, and they share a metal–metal bonding orbital of hybrid s/p/d character populated by one electron. The availability of dimetalloazafullerenes is limited by their relatively low yield in arc-discharge synthesis. Fortunately, single-electron metal–metal bonds in EMFs were open to a more detailed exploration after M_2_^5+^ dimers were stabilized inside more abundant all-carbon fullerenes by functionalization with the benzyl group, as in M_2_@C_80_(CH_2_Ph) (M_2_ = Sc_2_, Y_2_, La_2_, Gd_2_, Tb_2_, TbY, Dy_2_, Ho_2_, and Er_2_),^54–57^ and later with CF_3_, as in M_2_@C_80_(CF_3_) (M_2_ = Y_2_, Nd_2_, and Tb_2_).^58,59^ The first example of the nonfullerene single-electron lanthanide–lanthanide bond was also recently obtained in the (Cp^*i*Pr5^)_2_M_2_I_3_ complex.^60^

For molecular magnetism, it is particularly important that the unpaired electron residing on the metal–metal bond interacts strongly with the nuclear and electron spins of the metal atoms. Thus, dimetallofullerenes (di-EMFs) of Sc, Y, and La with a single-electron M–M bond exhibit large hyperfine coupling with nuclear spins of metals.^51,54,56–58,61,62^ For Gd di-EMFs, colossal ferromagnetic Gd-*e* exchange coupling was established first computationally^63,64^ and then experimentally,^65,66^ turning Gd_2_^5+^ into a giant-spin system with *S* = 15/2. For anisotropic lanthanides, particularly Tb and Dy, di-EMFs M_2_@C_79_N, M_2_@C_80_(CH_2_Ph), and M_2_@C_80_(CF_3_) were found to be hard single-molecule magnets with long spin-lattice relaxation times and very broad magnetic hysteresis.^53,55,56,58,67^ Spin-density distributions of all these di-EMFs plot the picture of the unpaired electron trapped between two metals, which are in their turn isolated inside the fullerene and shielded from a potentially aggressive environment. Despite the unusual oxidation state of metals, di-EMFs are remarkably air-stable and can be further functionalized and processed as self-assembled monolayers under ambient conditions,^68^ while azafullerenes M_2_@C_79_N can be also sublimed without decomposition.^69^

The spatial isolation of the M_2_^5+^ dimer inside the fullerene akin to N@C_60_ suggests that the spin-lattice relaxation and spin coherence in di-EMFs might be long. Preliminary report on Y_2_@C_79_N,^70^ comprehensive studies of Sc_2_@C_80_(CH_2_Ph),^54,71,72^ and more recent analysis of mixed-metal CaY@C_82_ ^73^ and CaSc@C_82_ ^74^ demonstrated that di-EMFs with *S* = 1/2 indeed demonstrate reasonably long coherence times and Rabi oscillations, albeit not as impressive as in N@C_60_. Complexes of rare-earth metals with larger spins can be also of interest as qubit candidates.^75–91^ Although their relaxation times are usually shortened by spin-orbit and spin-spin interactions, the possibility to encode several qubits or implement quantum gates and error corrections within a single spin system is their important advantage,^83,92–99^ which can offset their shorter coherence times. Studies of spin coherence in lanthanide EMFs have been limited so far to the 4f^7^ systems Eu^II^@C_2*n*_ (2*n* = 74, 80, 82, 84),^100^ Gd^III^@C_82_(morpholine)_5,7,9_,^101^ and the di-EMF Gd_2_@C_79_N.^65^

In this work, we conducted a systematic study of spin dynamics in rare-earth M_2_^5+^ dimers (M = Y, Gd), stabilized in M_2_@C_80_(CH_2_Ph) di-EMF derivatives. The endohedral spin system on this molecular platform can be varied by gradual substitution of Y by Gd in essentially identical structural settings. This allowed us to synthesize and study Y_2_@C_80_(CH_2_Ph), YGd@C_80_(CH_2_Ph), and Gd_2_@C_80_(CH_2_Ph) and explore how the variation of the total spin from *S* = 1/2 through *S* = 4 to *S* = 15/2 is reflected in the spin-lattice relaxation and spin coherence of endohedral metal dimers. By comparing two isomers of Y_2_@C_80_(CH_2_Ph) with *I*_*h*_ and *D*_5*h*_ cage symmetry, we also analyze the influence of the fullerene host and demonstrate that the isomers exhibit considerably different spin-lattice relaxation times, which is rationalized based on the analysis of their vibrational spectra and spin-phonon couplings. The use of high-field W-band EPR spectroscopy throughout this work facilitated the resolution of different molecular orientations and the analysis of the spatial anisotropy of spin-lattice and spin-spin relaxation.

## Results and Discussion

### Molecular and Spin Structures of M_2_@C_80_(CH_2_Ph)

#### Synthesis and Characterization

The synthesis of Y_2_@*I_h_*-C_80_(CH_2_Ph) and Gd_2_@*I_h_*-C_80_(CH_2_Ph) (abbreviated hereafter as {Y_2_-*I_h_*} and {Gd_2_-*I_h_*}, respectively) was reported by us earlier.^55,56^ Briefly, the arc-discharge evaporation of graphite rods filled with metal oxide and graphite powder in a He atmosphere was followed by extraction of soot with boiling dimethylformamide and the reaction of the extract with benzyl bromide at elevated temperature. The mixture of benzyl adducts of various fullerenes was then separated by high-performance liquid chromatography (HPLC). To obtain YGd@*I*_*h*_-C_80_(CH_2_Ph) ({YGd-*I*_*h*_} hereafter), the same synthetic procedures were performed using a mixture of Gd_2_O_3_ and Y_2_O_3_ oxides. The process yielded a mixture of {Y_2_-*I*_*h*_}, {YGd-*I*_*h*_}, and {Gd_2_-*I*_*h*_}, among other metallofullerene derivatives. Despite a very similar retention behavior, the separation of {YGd-*I*_*h*_} from {Y_2_-*I*_*h*_} and {Gd_2_-*I*_*h*_} could be accomplished by recycling HPLC, as described in Figure S1. Mass spectra of individual {Y_2_-*I*_*h*_}, {YGd-*I*_*h*_}, and {Gd_2_-*I*_*h*_} (Figure 1a) confirm the compositional purity of the samples studied further by EPR, while nearly identical vis–NIR absorption spectra (Figure 1b) prove that they are isostructural and have the same *I*_*h*_-C_80_ fullerene cage.

**Figure 1:**
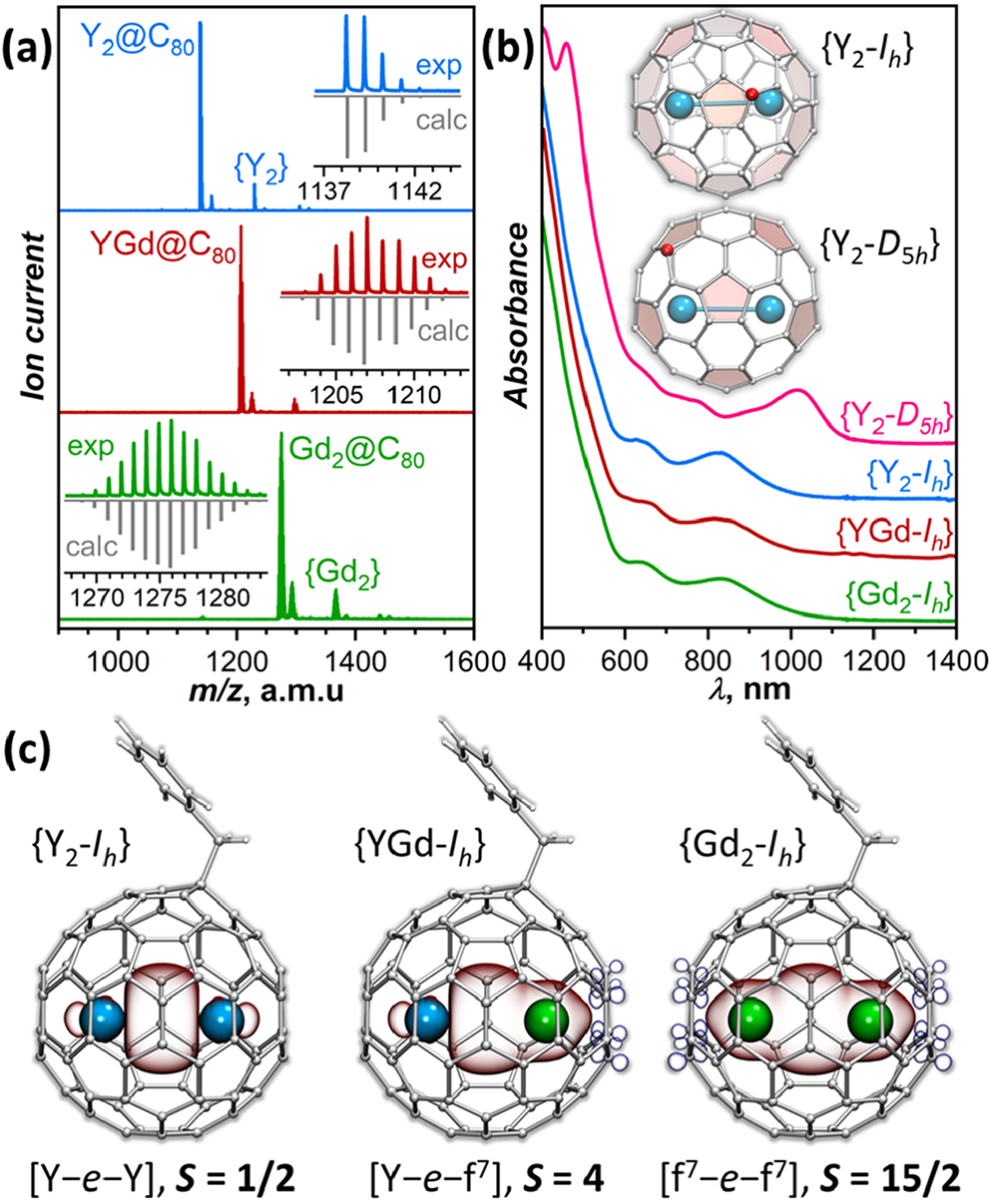
(a) MALDI-TOF mass-spectra of {Y_2_-*I*_*h*_}, {YGd-*I*_*h*_}, and {Gd_2_-*I*_*h*_} (positive ion mode, matrix: tetraphenyl butadiene, strong fragmentation under MALDI conditions results in much higher intensity of M_2_@C_80_^+^ fragments than molecular {M_2_}^+^ ions); insets compare the measured and calculated isotopic patterns of M_2_@C_80_. (b) Vis–NIR absorption spectra of {Y_2_-*D*_5*h*_}, {Y_2_-*I*_*h*_}, {YGd-*I*_*h*_}, and {Gd_2_-*I*_*h*_} in toluene solution; the inset compares the *D*_5*h*_ and *I*_*h*_ cage isomers of Y_2_@C_80_(CH_2_Ph) viewed along the *C*_5_ axis of the fullerene cage perpendicular to the Y–Y bond, the carbon atom bonded to the CH_2_Ph group is highlighted in red. (c) DFT-computed spin density in {Y_2_-*I*_*h*_}, {YGd-*I*_*h*_}, and {Gd_2_-*I*_*h*_} (PBE0-DKH/TZVP method, Orca suite;^102^ Y: blue; Gd: green; spin-density isosurfaces are semitransparent).

During the separation of the Y-EMF mixture, we identified and isolated a new isomer of Y_2_@C_80_(CH_2_Ph) with different retention time than that of {Y_2_-*I*_*h*_} (Figure S2). Its vis–NIR absorption spectrum is also quite different from that of {M_2_-*I*_*h*_} species (Figure 1b) but exhibits a close similarity to the spectra of La_2_@*D*_5*h*_-C_80_(C_3_N_3_Ph_2_)^61^ and Nd_2_@*D*_5*h*_-C_80_(CF_3_).^59^ Given the high sensitivity of absorption spectra of EMFs to the isomeric structure of fullerene cages and addition pattern of exohedral groups, we conclude that the new isomer of Y_2_@C_80_(CH_2_Ph) has the *D*_5*h*_-C_80_ fullerene cage. This structure is hereafter denoted as {Y_2_-*D*_5*h*_}.

#### Spin Structure and Spin-Density Distribution

Before going into the details of EPR studies, it is useful to briefly review the electronic structure and spin-density distribution of {M_2_-*I*_*h*_} species based on density functional theory (DFT) calculations.^50,55,56^ {Y_2_-*I*_*h*_} has an unpaired electron residing on the Y–Y σ-bonding orbital. The spin density of the molecule (Figure 1c) therefore coincides with the orbital density of the SOMO and is mainly localized between two Y atoms. Contributions of Y atomic orbitals to the Mulliken spin population are 2 × 26% *s*, 2 × 16% *d*, and 2 × 8% *p*, giving the net spin population of 50% for each metal atom. If the *z*-axis is chosen parallel to the Y–Y bond, *d* and *p* AO contributions to the Y–Y σ-bonding MO are mainly given by *d*_*z* ^2^_ (13%) and *p*_*z*_ (8%), with a small admixture of *d*_*xz*_ and *d*_*yz*_ (∼1.5% each). Note that the decomposition into *s*, *p*, and *d* components is not unique, and other approaches to population analysis may give different results.

In addition to the metal–metal bonding SOMO, which is nearly identical to that in {Y_2_-*I*_*h*_}, {Gd_2_-*I*_*h*_} also has 4f^7^ electrons on each Gd atom. Computational, EPR, and magnetometry studies showed that the ground spin state of the [4f^7^–e–4f^7^] system has a ferromagnetic alignment of all spin moments, giving the total spin of *S*_tot_ = 15/2.^52,63–66^ The spin-density distribution of this state looks like a superposition of the SOMO density and nearly spherical 4f^7^ spin distributions in each Gd (Figure 1c). The large Gd-localized spin results in some spin polarization of the metal-coordinated carbons. Spin polarization of fullerene carbons is also present in {Y_2_-*I*_*h*_}, but it is much weaker and is not seen when plotted at the same isodensity value. If {Gd_2_-*I*_*h*_} is treated as a 3-center spin system, its spin Hamiltonian has the following form:

$${Ĥ}_{\mathrm{spin}}=-2j_{1,2}{Ŝ}_{{\mathrm{Gd}}^{\prime }}{Ŝ}_{{\mathrm{Gd}}^{\prime \prime }}-2j_{\mathrm{Gd},e}ŝ\left({Ŝ}_{{\mathrm{Gd}}^{\prime }}+{Ŝ}_{{\mathrm{Gd}}^{\prime \prime }}\right)$$ 1

where *Ŝ*_Gd^′^_ and *ŝ* are spin operators of Gd and the unpaired valence electron, while *j*_1,2_ and *j*_Gd,e_ are Gd–Gd and Gd–e spin-coupling parameters. Broken-symmetry (BS) DFT calculations for {Gd_2_-*I*_*h*_} predicted *j*_Gd,e_ = 181–184 cm^–1^ and *j*_1,2_ = −1.2 cm^–1^,^56^ while the experimental effective coupling constant *j*_Gd,e_^eff^ = 160 ± 10 cm^–1^ was determined under the assumption that *j*_1,2_ is zero.^55^ With such a large coupling, only the ground spin state with *S*_tot_ = 15/2 contributes to the magnetic properties of {Gd_2_-*I*_*h*_} up to ∼100 K, and even at room temperature, the population of this state exceeds that of all othes state taken together.

The mixed-metal system {YGd-*I*_*h*_} has the properties of both of its homometallic congeners. Y^3+^ and Gd^3+^ have similar ionic radii, and the valence-spin distribution due to the Y–Gd bond is quite symmetric, while the Gd side also has an additional spin density arising from 4f^7^ electrons. In the Y and Gd halves of the {YGd-*I*_*h*_} molecule, the spin-density distribution looks exactly like in {Y_2_-*I*_*h*_} and {Gd_2_-*I*_*h*_}, respectively (Figure 1c). In other words, the substitution of one metal from Gd to Y in {Gd_2_-*I*_*h*_} does not affect the remaining Gd–e interaction, and vice versa, while the substitution of Y by Gd in {Y_2_-*I*_*h*_} does not change the spin distribution on the remaining Y–e part. This is quite remarkable, given the large spin of Gd and the spin polarization it might induce (as in the Gd-coordinated carbons). BS-DFT predicts the ferromagnetic ground state of {YGd-*I*_*h*_} with *S*_tot_ = 4, the energy gap of 1198 cm^–1^ to the state with *S*_tot_ = 3, and the opposite alignment of 4f^7^ and valence-electron spins. Projecting this onto the exchange Hamiltonian *Ĥ*_spin_ = −2*j*_Gd,e_ *ŝŜ*_Gd_ gives *j*_Gd,e_ = 171 cm^–1^, very similar to the *j*_Gd,e_ value in {Gd_2_-*I*_*h*_}. In view of this strong ferromagnetic coupling, the magnetic properties of {YGd-*I*_*h*_} are expected to be predominantly defined by its ground state with *S*_tot_ = 4.

### EPR Spectra of M_2_@C_80_(CH_2_Ph)

#### EPR Spectra of {Y_2_-*I*_*h*_} and {Y_2_-*D*_5*h*_}

A strong interaction between the unpaired electron spin residing on the metal–metal bond and nuclear spins of ^89^Y (100%, *I*_Y_ = 1/2) gives a pronounced hyperfine structure in the EPR spectra of {Y_2_-*I*_*h*_} and {Y_2_-*D*_5*h*_} (Figure 2a,b). At room temperature in toluene solution, {Y_2_-*I*_*h*_} shows a triplet with a 1:2:1 ratio as expected for two equivalent Y atoms. The large isotropic hyperfine coupling (hfc) constant *a*_iso_(^89^Y) of 224 MHz reflects a considerable Y-5s contribution to the Y–Y bonding orbital and spin density. While the molecular structure of {Y_2_-*I*_*h*_} has low symmetry, the equivalence of two ^89^Y hfc constants points to the fast circulation of the metal dimer inside the fullerene cage on the EPR time scale.^56^ Indeed, DFT calculations of {M_2_-*I*_*h*_} adducts showed the presence of several nearly isoenergetic minima for M_2_ positions, while DFT-based Born–Oppenheimer molecular dynamics (BOMD) revealed a considerable rotational motion of the metal dimer during 60 ps at 300 K (Figure 3a, see also the discussion in ref 56). Variable-temperature SC-XRD study of {Dy_2_-*I*_*h*_} also showed a strong increase of rotational disorder of the metal dimer from 100 to 300 K.^55^ Since metal atoms tend to avoid the cage C-sp^3^ atom bearing an exohedral group, the metal dimer circulation in {M_2_-*I*_*h*_} preferentially occurs along the belt of hexagons, located in the plane roughly perpendicular to the C_cage_–C_addend_ bond.

**Figure 2:**
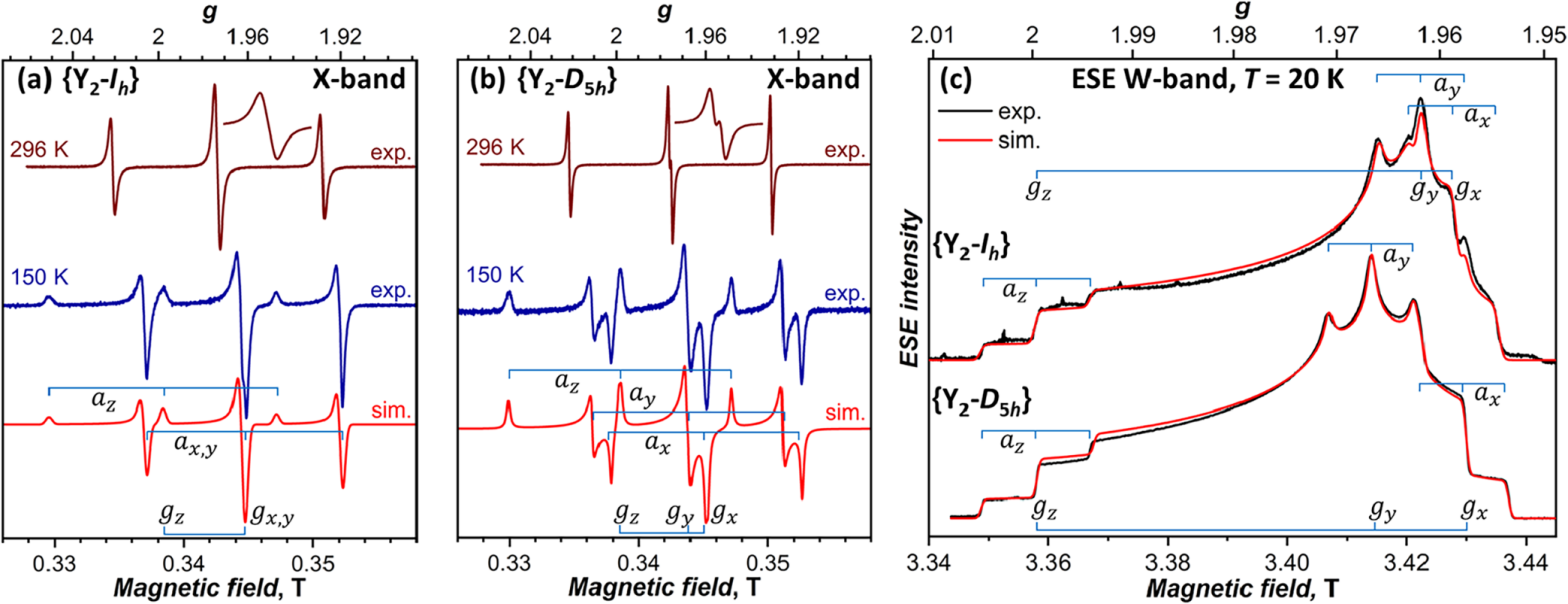
(a) X-band EPR spectra of {Y_2_-*I*_*h*_} in liquid and frozen toluene solution (296 and 150 K, respectively) compared to simulations with EasySpin^103^ for 150 K; (b) the same as (a) but for {Y_2_-*D*_5*h*_}. The insets in (a, b) magnify the central line in the isotropic spectra, showing a broadened singlet for *I*_*h*_ and a doublet for *D*_5*h*_ isomers. (c) ESE-detected W-band spectra of {Y_2_-*I*_*h*_} and {Y_2_-*D*_5*h*_} in frozen *d*_8_-toluene at 20 K (black: experiment; red: simulations). Small sharp peaks in the W-band spectrum of {Y_2_-*I*_*h*_} between 3.34 and 3.38 T are caused by residual amounts of Mn impurity in the cavity.

**Figure 3:**
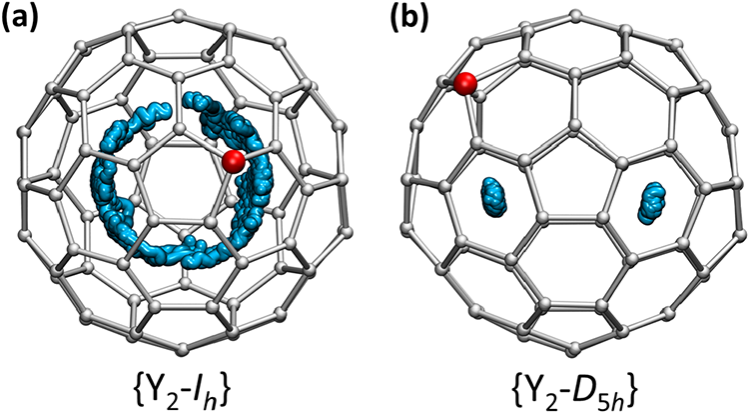
Trajectories of Y atoms in (a) {Y_2_-*I*_*h*_} and (b) {Y_2_-*D*_5*h*_} obtained in DFT-based Born–Oppenheimer molecular dynamics (*T* = 300 K; total propagation time = 60 ps; PBE/DZVP level; CP2K package^105^). Oscillations of carbon atoms near their equilibrium positions are not shown. The addition sites of the benzyl group, which was replaced with CH_3_ in BOMD simulations, are marked in red.

A different dynamic situation is found for {Y_2_-*D*_5*h*_}. Although its room-temperature isotropic EPR spectrum is an apparent triplet (Figure 2b), the splitting of the central line indicates that two Y atoms may not be equivalent, and the spectrum can be described by two slightly different *a*_iso_(^89^Y) constants of 213 and 218 MHz. This finding is consistent with previous DFT studies of M_2_@*D*_5*h*_-C_80_ monoadducts,^61^ which revealed a single preferable position of the metal dimer with a considerable energy gap to other orientations. Similarly, our BOMD simulations showed that Y atoms only oscillate near their equilibrium positions during the whole 60 ps run at 300 K (Figure 3b). Thus, a less symmetric *D*_5*h*_-C_80_ cage has a more anisotropic environment compared to *I*_*h*_-C_80_, which is further enhanced by exohedral addition to a nonsymmetric position in {Y_2_-*D*_5*h*_} and altogether leads to the hindered dynamics of the metal dimer.

Aside from the different internal dynamics, {Y_2_-*I*_*h*_} and {Y_2_-*D*_5*h*_} have a very similar spin distribution as follows from the values of their isotropic *g*-factors and hyperfine constants. Lower-temperature measurements in frozen solution revealed that the anisotropy of hyperfine and especially *g*-tensors is more sensitive to the fullerene cage isomerism. At 150 K, below the solvent melting point, fullerene molecules are frozen in the toluene matrix and show powder-like X-band EPR spectra (Figure 2a,b). Hyperfine tensors are axial for both cage isomers, with *a*_*z*_ values exceeding *a*_*x*,*y*_ at ∼40 MHz. For {Y_2_-*D*_5*h*_}, we cannot resolve the difference in the hyperfine parameters of two Y atoms anymore, and the fit of the spectra gave nearly identical values within the uncertainty of 1–2 MHz. The *g*-tensor is axial for the {Y_2_-*I*_*h*_} isomer but is clearly rhombic for the {Y_2_-*D*_5*h*_} isomer (Table 1). Note that the *g*_*z*_ principal values and the average of *g*_*x*_ and *g*_*y*_ are nearly equal for both structures. The higher *g*-tensor symmetry in {Y_2_-*I*_*h*_} is presumably an effect of residual circulation of the metal dimer. When the temperature is lowered further to 100 K, the spectrum of {Y_2_-*I*_*h*_} exhibits additional splitting (Figures S3 and S4) caused by the divergence of *g*_*x*_ and *g*_*y*_.

**Table 1:** Spin Parameters (***g*** *-* and ***A*** *-*Tensors) of {Y_2_-*I*_h_} and {Y_2_-*D*_5_ *_h_*}

	*g*	*A*, MHz	conditions	refs^a^
{Y_2_-*I*_*h*_}	*g*_iso_ = 1.973	*a*_iso_ = 224	X-band, 296 K	^56^
	*g*_*x*,*y*_ = 1.962, *g*_*z*_ = 1.998	*a*_*x*,*y*_= 208, *a*_*z*_ = 246	X-band, 150 K	^56^
	*g*_*x*_ = 1.958, *g*_*y*_ = 1.962, *g*_*z*_ = 1.998	*a*_*x*,*y*_ = 199, *a*_*z*_ = 258	W-band, 20 K	t.w.
	*g*_*x*_ = 1.965, *g*_*y*_ = 1.970, *g*_*z*_ = 2.002	Y1: *a*_*x*,*y*_= −190, *a*_*z*_ = −223	DFT PBE-ZORA	t.w
		Y2: *a*_*x*,*y*_= −180, *a_z_* = −213		
{Y_2_-*D*_5*h*_}	*g*_iso_ = 1.974	*a*_iso_ = 213, 218	X-band, 296 K	t.w.
	*g*_*x*_ = 1.958, *g*_*y*_ = 1.966, *g*_*z*_ = 1.997	*a*_*x*,*y*_ = 202, *a*_*z*_ = 241	X-band, 150 K	t.w.
	*g*_*x*_ = 1.957, *g*_*y*_ = 1.967, *g*_*z*_ = 1.999	*a*_*x*,*y*_ = 198, *a*_*z*_ = 260	W-band, 20 K	t.w.
	*g*_*x*_ = 1.962, *g*_*y*_ = 1.971, *g*_*z*_ = 1.999	Y1: *a*_*x*,*y*_= −190, *a*_*z*_ = −223	DFT PBE-ZORA^b^	t.w.
		Y2: *a*_*x*,*y*_= −187, *a_z_* = −220		

a “t.w.” means this work.

b DFT calculations were performed using the PBE functional with ZORA scalar relativistic correction and ZORA-adjusted def2-TZVP basis sets as implemented in Orca.^102,104^

The equivalence of Y atoms in the low-temperature spectrum of {Y_2_-*D*_5*h*_} indicates that splitting of the central line in the room-temperature spectrum may be caused by dynamic factors rather than different coupling constants. Indeed, the intensity distribution in the room-temperature spectrum of {Y_2_-*D*_5*h*_} deviates from the 1:2:1 ratio, suggesting that the rotational averaging is not complete. To better understand this phenomenon, we studied EPR spectra of {Y_2_-*I*_*h*_} and {Y_2_-*D*_5*h*_} in liquid toluene between 296 and 176 K and fitted them under the assumption of the slow-motion regime using the function *chili* of EasySpin (Figures S3 and S4). The rotational correlation time (*T*_corr_) and Lorentzian line width were used as fitting parameters, whereas *g*- and A-tensors were fixed to values determined in the frozen solution at 150 K. The *T*_corr_ of {Y_2_-*D*_5*h*_} gradually shortens from 3.43 ns at 176 K to 0.06 ns at 296 K. Importantly, simulations in the slow-motion regime predict the splitting of the central line between 236 and 296 K. In {Y_2_-*I*_*h*_}, a similar splitting is observed at lower temperatures, because its *T*_corr_ values below 270 K are twice shorter than in {Y_2_-*D*_5*h*_}. At 276 and 296 K, the spectra of {Y_2_-*I*_*h*_} are essentially isotropic, and *T*_corr_ values of 0.01 ns determined for these temperatures are probably not very reliable. Above 270 K, {Y_2_-*I*_*h*_} also shows anomalous broadening of the line width. The exact reason for this effect is not clear at this moment, but an interplay of the molecular rotation and the fast rotation of the Y_2_ cluster is a plausible suspect.

The measurements at helium temperatures were performed in pulse mode with Electron Spin Echo (ESE) detection at the W-band spectrometer with a home-built EPR/ENDOR microwave cavity.^106–108^ The 10-fold increase in the microwave frequency and resonance magnetic field changed the spectral pattern of Y_2_@C_80_(CH_2_Ph): While the hyperfine splitting is comparable to the *g*-tensor anisotropy in the X-band spectra, the W-band spectra are dominated by the *g*-tensor anisotropy with additional hyperfine splitting at turning points corresponding to *g*_*x*,*y*,*z*_ values (Figure 2c). At 20 K, when all internal dynamics can be considered frozen, a rhombic *g*-tensor is found for both isomers, but {Y_2_-*D*_5*h*_} still has a more pronounced difference between *g*_*x*_ and *g*_*y*_ (Table 1). The *A*-tensor preserves its axial structure, but cooling from 150 to 20 K increases its apparent anisotropy, and in particular the value of the *a*_*z*_ constants (Table 1).

DFT calculations at the PBE-ZORA level reproduce the similarity of the *g*- and A-tensors of both isomers (Table 1). The tensors are collinear with *z* principal axes aligned along the Y–Y bond. The *A*-tensor anisotropy is consistent with the large contribution of 5s orbitals to the SOMO, ensuring a substantial isotropic part, but also reflects the contribution of 4d and 5p AOs, which induces a certain degree of anisotropy. Anisotropy of the *g*-tensor in {Y_2_-*I*_*h*_} and {Y_2_-*D*_5*h*_}, with *g*_*z*_ being close to the free-electron value *g*_e_, and *g*_*x*,*y*_ showing more pronounced deviations from *g*_e_, can be qualitatively understood based on simple orbital considerations. In the first-order linear response theory, deviations of principal *g*-tensor components from *g*_e_ in a radical with a nondegenerate ground state are caused by the admixture of excited states through spin-orbit coupling (SOC):

2 $${\Delta g}_{x\left(y,z\right)}=-2\lambda \sum_{j}\frac{\left\langle \Psi_{0}\right|{\mathrm{L̂}}_{x\left(y,z\right)}\left|\Psi_{j}\right\rangle \left\langle \Psi_{j}\right|{\mathrm{L̂}}_{x\left(y,z\right)}\left|\Psi_{0}\right\rangle }{\Delta E_{0\to j}}$$

where λ is the spin-orbit coupling constant, Ψ_0_ (Ψ_*j*_) is the ground (excited)-state wave function, Δ*E*_0→*j*_ is the excitation energy, and *L̂*_*x*(*y*,*z*)_ are orbital angular momentum operators. For the following analysis, it is sufficient to consider only orbitals localized on the Y_2_^5+^ dimer and their Y-AO components. The σ-bonding SOMO is a linear combination of *s*, *d*_*z* ^2^_, and *p*_*z*_, while the two lowest-energy unoccupied orbitals have π_*x*_ and π_*y*_-bonding character and are built mainly upon *d*_*xz*_ and *d*_*yz*_ Y-AOs. The action of *L̂*_*z*_ upon *d*_*z* ^2^_ and *p*_*z*_ gives zero, while *d*_*xz*_ (*d*_*yz*_) are transformed by *L̂*_*z*_ to *d*_*yz*_ (*d*_*xz*_). Therefore, the only nonzero contributions to Δ*g*_*z*_ appear from small (∼1.5%) admixtures of *d*_*xz*_ and *d*_*yz*_ to the SOMO. *L̂*_*x*_ and *L̂*_*y*_ operators produce more substantial values of Δ*g*_*x*_ and Δ*g*_*y*_ because *L̂*_*x*(*y*)_|*d*_*z* ^2^_⟩ → ± *i*√3*d*_*yz*(*xz*)_ and *L̂*_*x*(*y*)_|*d*_*yz*(*xz*)_⟩ → *i*(*d*_*x* ^2^–*y* ^2^_ ± √3*d*_*z* ^2^_), and thus *d*_*xz*_ and *d*_*yz*_ AOs in unoccupied MOs will couple to *d*_*z* ^2^_ in the SOMO after transformation by *L̂*_*x*(*y*)_ and *vice versa*. Note that *d*_*z* ^2^_ does not couple to *d*_*x* ^2^–*y* ^2^_ and *d*_*xy*_ through angular momentum operators. The π_*x*_ and π_*y*_ MOs in the isolated Y_2_^5+^ dimer are degenerate, leading to equal Δ*g*_*x*_ and Δ*g*_*y*_ values and axial anisotropy, but the lower symmetry of *I*_*h*_-C_80_(CH_2_Ph) and *D*_5*h*_-C_80_(CH_2_Ph) fullerene hosts and mixing with the fullerene π-system lift the orbital degeneracy and change orbital compositions, thereby introducing some differences between *g*_*x*_ and *g*_*y*_ in {Y_2_-*I*_*h*_} and {Y_2_-*D*_5*h*_}.

Hyperfine couplings in {Y_2_-*I*_*h*_} and {Y_2_-*D*_5*h*_} with the formal Y^+2.5^ oxidation state can be compared with other Y-based radicals with unconventional Y oxidation states, which are also characterized by a dominant contribution of 5*s*/4d AOs to the spin density (Table S1). The *a*_iso_(^89^Y) values of {Y_2_-*I*_*h*_} and {Y_2_-*D*_5*h*_} are close to those in other endohedral Y_2_^5+^ dimers, which span the narrow range of 200–230 MHz.^51,58,68,109^ Similar constants are also reported for mixed-metal endohedral dimers, 200 MHz in ThY@*D*_3h_-C_78_,^110^ 252 MHz in CaY@*C*_2*v*_-C_80_, and 260 MHz in CaY@*C*_*s*_-C_82_.^73^ (Cp^iPr5^)_2_Y_2_I_3_ also has a Y_2_^5+^ dimer in its structure,^60^ but features a very small *a*_iso_(^89^Y) constant of 4 MHz, suggesting the negligible 5s contribution and predominantly 4*d*_*z* ^2^_-composition of the Y–Y bonding MO. Y­(II)-based radicals^22,111–120^ exhibit a wide spread of *a*_iso_(^89^Y) values from 49 MHz in Y­(NH­{2,6-(2,4,6-(^i^Pr)_3_C_6_H_2_)­C_6_H_3_})_2_ ^111^ to 505 MHz in Y­(Cp^*i*Pr5^)_2_,^120^ reflecting the variable balance between 5s and 4d AOs in their SOMO. The largest *a*_iso_(^89^Y) value of 801 MHz is found in the matrix-isolated YO radical.^121^ For all of these species, the *g*-factor is usually smaller than the free-electron value by 0.01–0.03.

#### EPR Spectra of {Gd_2_-*I*_*h*_} and {YGd-*I*_*h*_}

As the strong spin-spin exchange interaction in {Gd_2_-*I*_*h*_} and {YGd-*I*_*h*_} suggests that their EPR spectra are dominated by the ground spin states, we will limit our discussion to *S*_tot_ = 15/2 and *S*_tot_ = 4, respectively. The earlier X- and Q-band study of {Gd_2_-*I*_*h*_} showed the presence of a single-line spectrum at room temperature in solution, while cooling to 100 K revealed a complex structure in the spectra caused by zero-field splitting (ZFS).^55^ Despite the overall complexity, the spectra could be well reproduced by simulations with a standard ZFS Hamiltonian ([Disp-formula eq3]):

3 $${Ĥ}_{\mathrm{spin}}=D\left({Ŝ}_{z}^{2}-\frac{1}{3}S\left(S+1\right)\right)+E\left({Ŝ}_{x}^{2}-{Ŝ}_{y}^{2}\right)+\mu_{B}\mathrm{Bg}Ŝ$$

with an isotropic *g*-factor of 1.987, ZFS parameters *D* = 1.03 GHz and *E* = 0.23 GHz, and ZFS strain of ∼0.03 GHz used to model the line broadening (Table 2, see Table S2 for comparison with parameters of other Gd-based metallofullerenes).

**Table 2:** Spin Parameters of {Gd_2_-*I_h_*} and {YGd-*I_h_*} Compared with Other Gd-Based Metallofullerenes with a Single-Electron M–M Bond

	*S*	*g*	*D*(strain), GHz	*E*(strain), GHz	conditions	refs
{Gd_2_-*I*_*h*_}	15/2	*g*_⊥_ = *g*_∥_ = 1.987	1.03 (0.029)	0.23 (0.027)	W-band, 6 K; X, Q-band, 100 K	t.w.;^55^
Gd_2_@C_79_N	15/2	*g*_⊥_ = *g*_∥_ = 1.99	0.96 (0.060)	0.14 (0.045)	X, Q-band, 3–8 K	^65,122^
[Gd_2_@*I*_*h*_-C_80_]^−^	15/2	*g*_⊥_ = *g*_∥_ = 1.99	1.02	0.31	Q-band, 6 K	^123^
{YGd-*I*_*h*_}	4	*g*_⊥_ = 1.977, *g*_∥_ = 1.988	3.75 (0.18)	0.41 (0.07)	W-band, 6 K	t.w.

Unlike the X-band spectrum, in which the resonances of {Gd_2_-*I*_*h*_} fall in the field range with multiple |*m*_*S*_⟩ level crossings, the 10-fold higher frequency of the W-band shifts the resonances to the field, in which |*m*_*S*_⟩ → |*m*_*S*_ + 1⟩ transitions are well-defined (Figure 4a–c). Thus, the ESE-W-band measurement at 6 K gives a much simpler spectral profile with clearly resolved steps corresponding to turning points of individual |*m*_*S*_⟩ → |*m*_*S*_ + 1⟩ transitions, which can be perfectly reproduced by simulations with the ZFS parameters mentioned above. Furthermore, the energy differences between the |*m*_*S*_⟩ levels in the high magnetic field are large enough to observe thermal depopulation of the higher-energy levels. Actually, only the transitions within the lower half of the spin 15/2 manifold contribute significantly to the signal observed at 6 K (Figure 4c). This thermal depopulation can be used to determine the sign of the ZFS *D* parameter. By comparing the spectral patterns obtained for {Gd_2_-*I*_*h*_} at 6 and 10 K with the corresponding calculated EPR spectra (Figure 4b), we can unambiguously conclude that *D* is positive. Note that the two stable isotopes of Gd have a nuclear spin 3/2, ^155^Gd (14.8%) with a *g*_*n*_ of −0.1715 and ^157^Gd (15.7%) with a *g*_*n*_ of −0.2265, but the abundance of these isotopes and their hyperfine splitting are not sufficient to be resolved in the EPR spectra of {Gd_2_-*I*_*h*_}.

**Figure 4:**
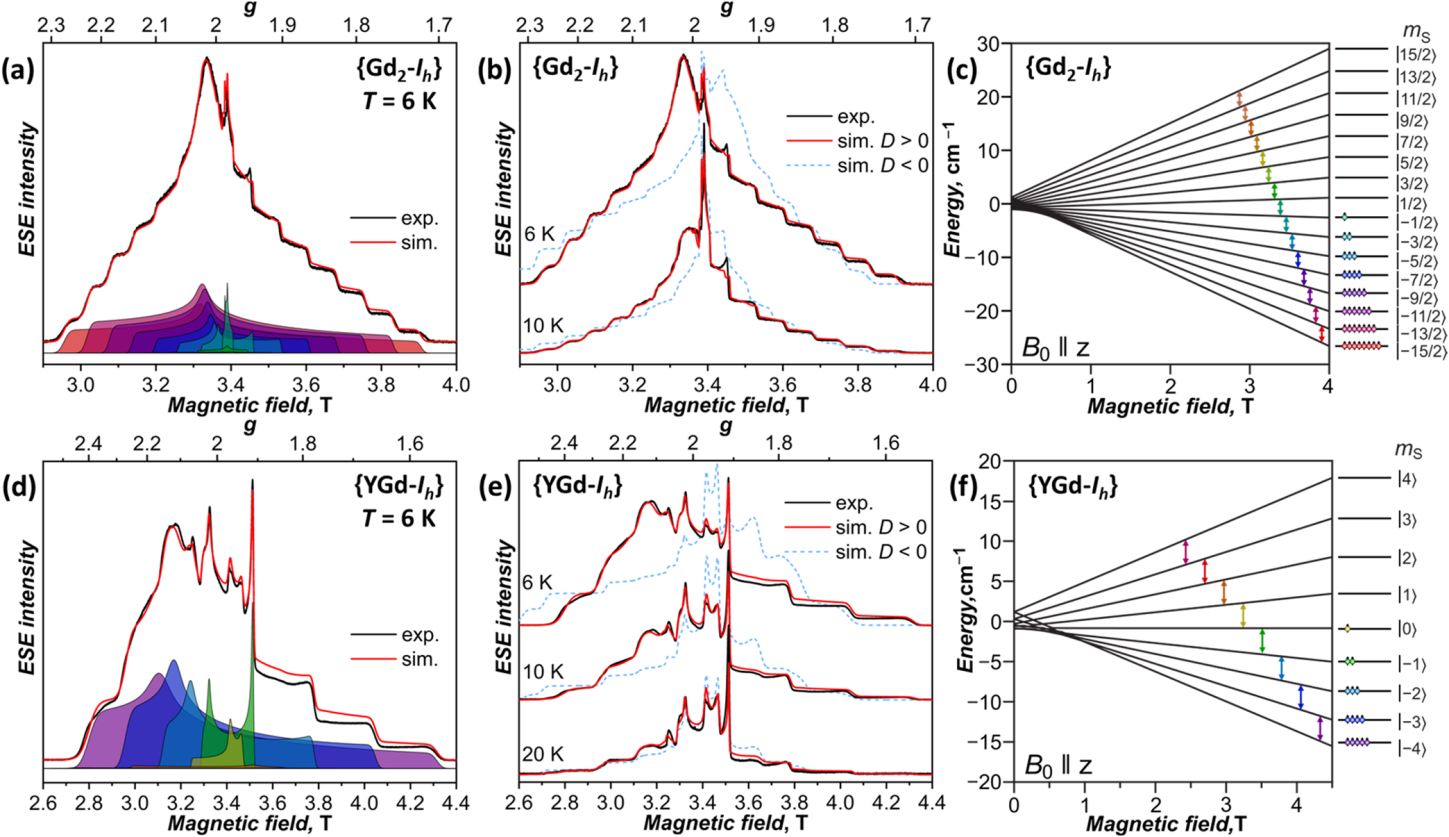
(a, d) ESE-detected W-band EPR spectra of {Gd_2_-*I*_*h*_} and {YGd-*I*_*h*_} measured at 6 K (black) compared to simulated (red) with spin Hamiltonian parameters discussed in the text and listed in Table 2; also shown are simulated contributions of individual |*m*_*S*_⟩ → |*m*_*S*_ + 1⟩ transitions, scaled according to the dependence of the flip angle θ = ω_nut_ *t* on the Rabi frequencies using the formula $\omega_{\mathrm{nut}}=\omega_{1}\sqrt{S\left(S+1\right)-m_{s}\left(m_{s}+1\right)}$ . (b, e) Spectra of {Gd_2_-*I*_*h*_} and {YGd-*I*_*h*_} measured at different temperatures, and simulations used to determine the sign of *D* (solid red curves: positive *D*; dashed blue curves: negative *D*). (c, f) Zeeman diagrams and W-band EPR transitions of {Gd_2_-*I*_*h*_} and {YGd-*I*_*h*_} for the field parallel to *z* principal axes of their *g*-tensors; colors of the arrows, denoting individual excitations, correspond to the colors of those transitions in (a, d). On the right, thermal populations of spin levels are shown schematically for ∼10 K.

For {YGd-*I*_*h*_}, the W-band spectrum (Figure 4d) allows similar, more straightforward analysis, than the X-band as the features of separate |*m*_*S*_⟩ → |*m*_*S*_ + 1⟩ transitions can be well distinguished. Fitting of the 6 K W-band spectrum with the Hamiltonian in eq 3 indicates a small *g*-tensor anisotropy (compare to the isotropic *g*-factor for {Gd_2_-*I*_*h*_}) and significantly higher *D* and *E* values than in {Gd_2_-*I*_*h*_}. The Zeeman splitting of |*m*_*S*_⟩ levels in the field of 3–4 T is sufficiently high to create a large difference in thermal populations, and a comparison of experimental and calculated spectra at different temperatures in the 6–20 K range proves the positive sign of *D*.

### Spin-Lattice Relaxation and Spin Coherence

#### Spin-Lattice Relaxation Times (*T*_1_) of {Y_2_-*D*_5_ *_h_*} and {Y_2_-*I_h_*}

The large resonance magnetic field in W-band EPR and significant *g*-tensor anisotropy enable orientation-selected measurements^124–127^ of spin relaxation times in {Y_2_-*D*_5*h*_} and {Y_2_-*I*_*h*_} since the field ranges with dominating *B*_0_∥*z* and *B*_0_∥*x*,*y* molecular orientations do not overlap (these will be dubbed *z* and *xy* domains in the discussion below). Furthermore, the measurements of *T*_1_ in these field ranges give straightforward access to the *T*_1_ anisotropy. Since we did not find a visible dependence of *T*_1_ on *m*_*I*_, we determine *T*_1,*z*_ and *T*_1,*xy*_ as average *T*_1_ values in respective field intervals (Figure 5) and then use them to compute *T*_1_ anisotropy ρ_*T*_1__ as the *T*_1,*z*_/*T*_1,*xy*_ ratio. See Figures S5 and S12 for inversion recovery data and Hahn echo decays.

**Figure 5:**
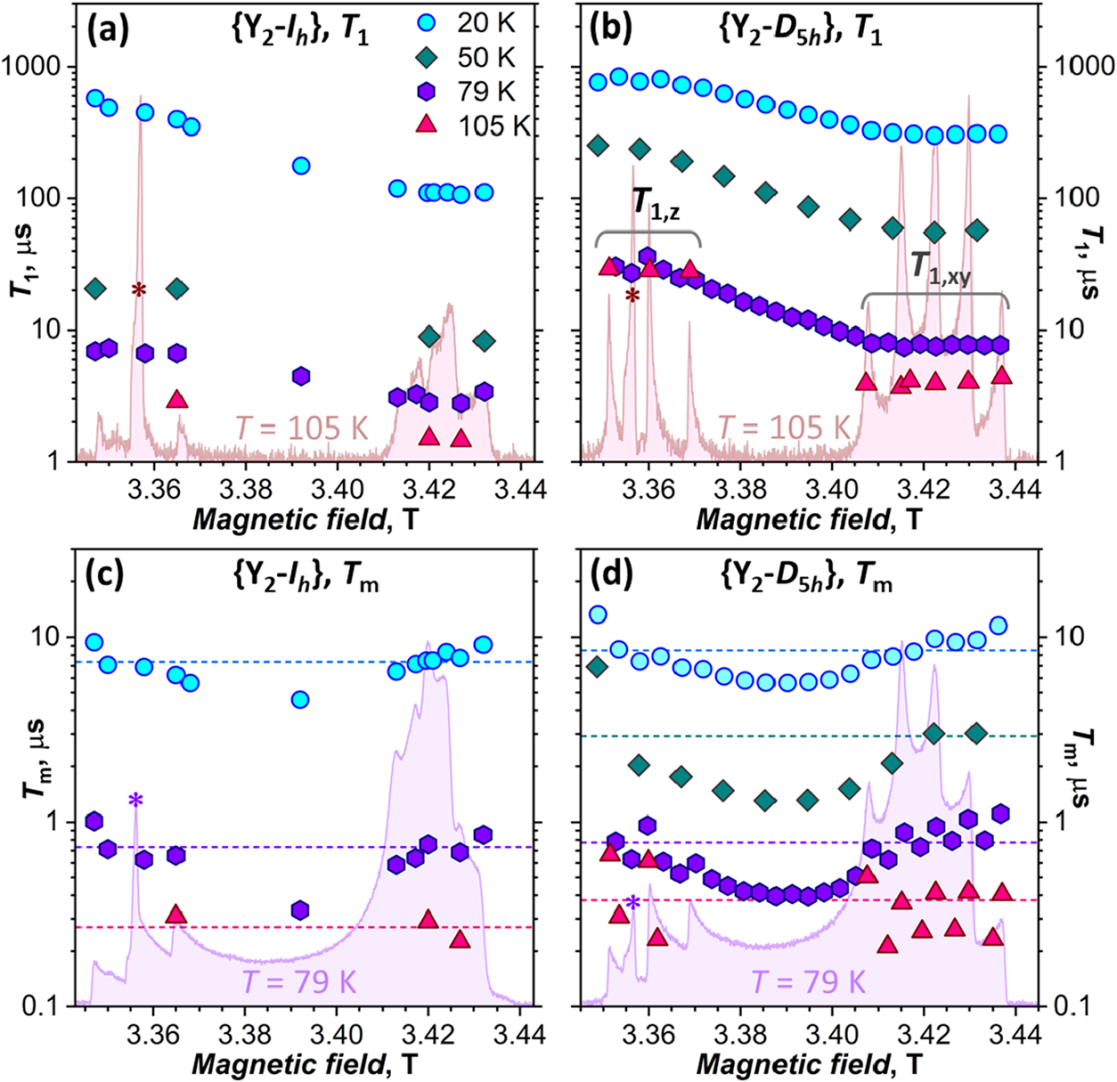
(a, b) Spin-lattice relaxation times *T*_1_ of {Y_2_-*I*_*h*_} and {Y_2_-*D*_5*h*_} measured at the W-band in frozen toluene-*d*_8_ at different temperatures and magnetic fields; ESE-W-band EPR spectra at *T* = 105 K are plotted in the background. (c, d) Phase-memory relaxation times *T*_m_ of {Y_2_-*I*_*h*_} and {Y_2_-*D*_5*h*_} measured under the same conditions as in (a, b); ESE-W-band EPR spectra at *T* = 79 K are plotted in the background. Dashed horizontal lines in (c, d), corresponding to *T*_m,*xyz*_ (average *T*_m_ values in fields parallel to *x*, *y*, and *z* molecular axes), are plotted to guide the eye only. The asterisks mark a radical impurity, producing sharp peak in ESE spectra near 3.357 T; it is seen only at higher temperatures, when *T*_m_ of fullerenes is shortened.

*T*_1_ measurements demonstrate systematic variation of the relaxation time with the molecular orientation with respect to the magnetic field, *T*_1,*z*_ being longer than *T*_1,*xy*_ by a factor of 2–4 (Table 3). The values in the intermediate region between the *z* and *xy* domains decrease almost linearly with the increasing field (Figure 5b). Over the temperature range of 20–105 K, *T*_1_ shortens by 2 orders of magnitude, from hundreds to few μs (Table 3, Figure 6a). At all of these temperatures, the spin-lattice relaxation of {Y_2_-*D*_5*h*_} stays several times longer than that of {Y_2_-*I*_*h*_}, showing that the carbon cage isomerism has a visible influence on *T*_1_.

**Figure 6:**
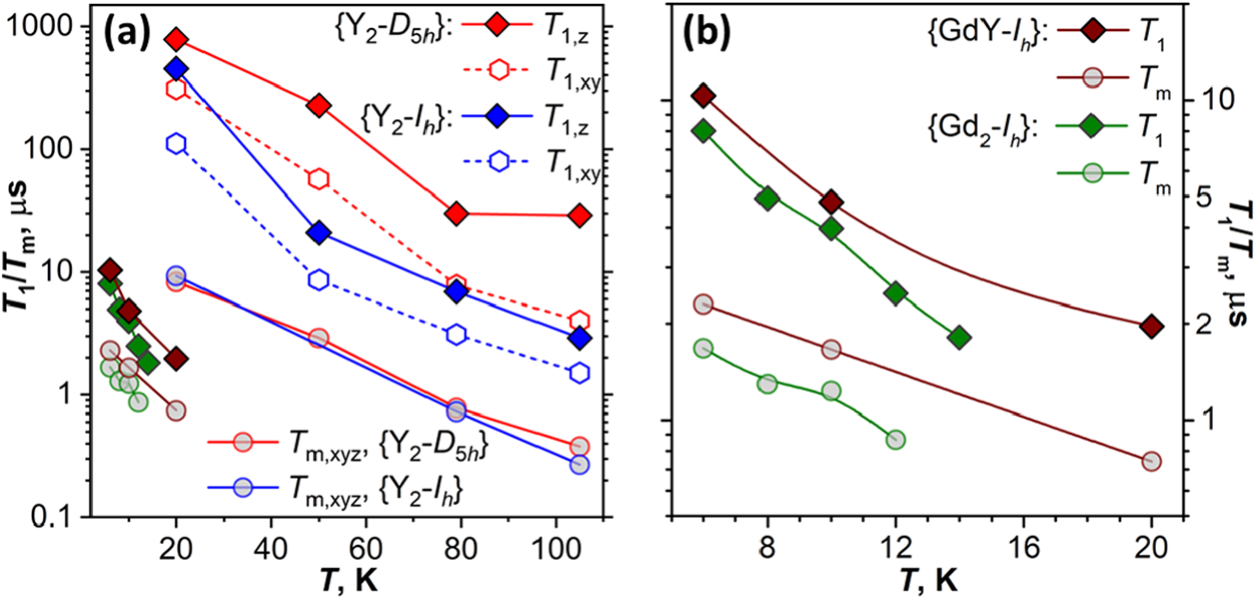
(a) *T*_1,*z*_, *T*_1,*xy*_, and *T*_m,*xyz*_ of {Y_2_-*D*_5*h*_} and {Y_2_-*I*_*h*_} measured at different temperatures. (b) *T*_1_ and *T*_m_ of {YGd-*I*_*h*_} (at 3.50 T) and {Gd_2_-*I*_*h*_} (at 3.35 T) measured at different temperatures. For completeness, data points of {YGd-*I*_*h*_} and {Gd_2_-*I*_*h*_} are also shown in (a). Lines connecting points are plotted to guide the eye only.

**Table 3:** *T*_1_, *T*_1_ Anisotropy (*ρ*_*T*_1__), and *T*_m_ of {Y_2_-*D*_5_ *_h_*} and {Y_2_-*I_h_*} Measured in the Field Ranges Corresponding to Molecular Orientations with *x*, *y*, and *z* Molecular Axes Parallel to the External Field

	*T*, K	*T*_1,*z*_, μs	*T*_1,*xy*_, μs	ρ_*T*_1__	*T*_m,*xyz*_, μs	*T*_m,mid_, μs
{Y_2_-*D*_5*h*_}	20	781	311	2.5	8.3	5.7
	50	228	57	4.0	2.9	1.3
	79	30	7.8	3.8	0.78	0.40
	105	29	4.0	7.1	0.38	
{Y_2_-*I*_*h*_}	20	451	111	4.1	7.4	4.6
	50	21	8.6	2.4		
	79	6.9	3.1	2.3	0.73	0.33
	105	2.9	1.5	1.9	0.27	

Such a type of *T*_1_ anisotropy was observed earlier for metal complexes and nitroxyl and organic radicals and is usually attributed to the modulation of spin-orbit coupling and consequently *g*-tensor anisotropy by molecular vibrations.^124,125,128–134^ It is assumed that a larger number of transverse vibrational modes and their stronger SOC result in the stronger modulation of *g*_*x*,*y*_ components, leading to shorter *T*_1,*xy*_ in comparison to *T*_1,*z*_. The spin-lattice relaxation in {Y_2_-*D*_5*h*_} and {Y_2_-*I*_*h*_} seems to closely follow this reasoning, and the stronger deviation of *g*_*x*,*y*_ from *g*_e_ alone indicates that it should be more susceptible to vibrational modulation than *g*_*z*_. However, Hadt et al. recently concluded that modulation of *g*_*i*_ components cannot be directly correlated with *T*_1,*i*_ and that the more refined approach requires analysis of the wave function and admixture of the opposite spin components by SOC, as these components and their modulation by vibrations are critical for the relaxation.^134^ Still, their model gave an asymptotic value of ρ_*T*_1__ = 2.5 under the assumption of one dominant vibrational mode and equal energies of excited states. In either case, microscopic analysis of the role of molecular vibrations is required to better understand the spin-lattice relaxation.

#### Vibrational Spectra of Y_2_@C_80_(CH_2_Ph) Isomers

When metal atoms are enclosed inside a fullerene, their three translational degrees of freedom transform into two vibrational modes with lateral and one with longitudinal character.^135,136^ In the former, metal atoms are displaced parallel to the fullerene surface, while the latter is sometimes described as a metal-cage stretching mode. Lateral modes of dimetallofullerenes can be further divided into two types: When two metal atoms oscillate in antiphase, overall vibrations have rotational (librational) character and occur at somewhat lower frequencies than in-phase oscillations, which have overall translational character (Figures S13–S15). In Y_2_@C_80_(CH_2_Ph), Y displacements in four lateral modes are parallel to the *xy* plane of the *g*-tensor and are parallel to the *g*-tensor z-axis in two longitudinal modes (Figure 7a). Our DFT calculations predict lateral modes in {Y_2_-*I*_*h*_} at 50, 55, 65, and 69 cm^–1^, while the {Y_2_-*D*_5*h*_} isomer features noticeably higher frequencies at 67, 82, 90, and 94 cm^–1^ (Figures 7b,c and S15). Although the harmonic approximation used in these calculations is questionable for the lateral modes, computed frequencies still reflect the local curvature of the potential energy surface and are in line with molecular dynamics simulations, predicting hindered internal rotation in the {Y_2_-*D*_5*h*_} isomer (Figure 3). Longitudinal modes occur at similar frequencies in both isomers, with the main contributions predicted at 175 and 195 cm^–1^ in {Y_2_-*I*_*h*_} and at 181 and 199 cm^–1^ in {Y_2_-*D*_5*h*_}. Unlike the lateral modes, these vibrations are not fully localized on metal dimers and are partially mixed with radial carbon cage vibrations in the 170–330 cm^–1^ range. Fullerene cages are usually rigid, and their vibrations start at 170 cm^–1^ and dominate the spectra at frequencies greater than 200 cm^–1^. In addition, benzyl groups have several low-frequency modes with librational and translational character, which occur at the same frequencies in both isomers, including three modes at 20–45 cm^–1^, two at 110 and 125 cm^–1^, and one close to the longitudinal mode near 180 cm^–1^, with which it partially mixes.

**Figure 7:**
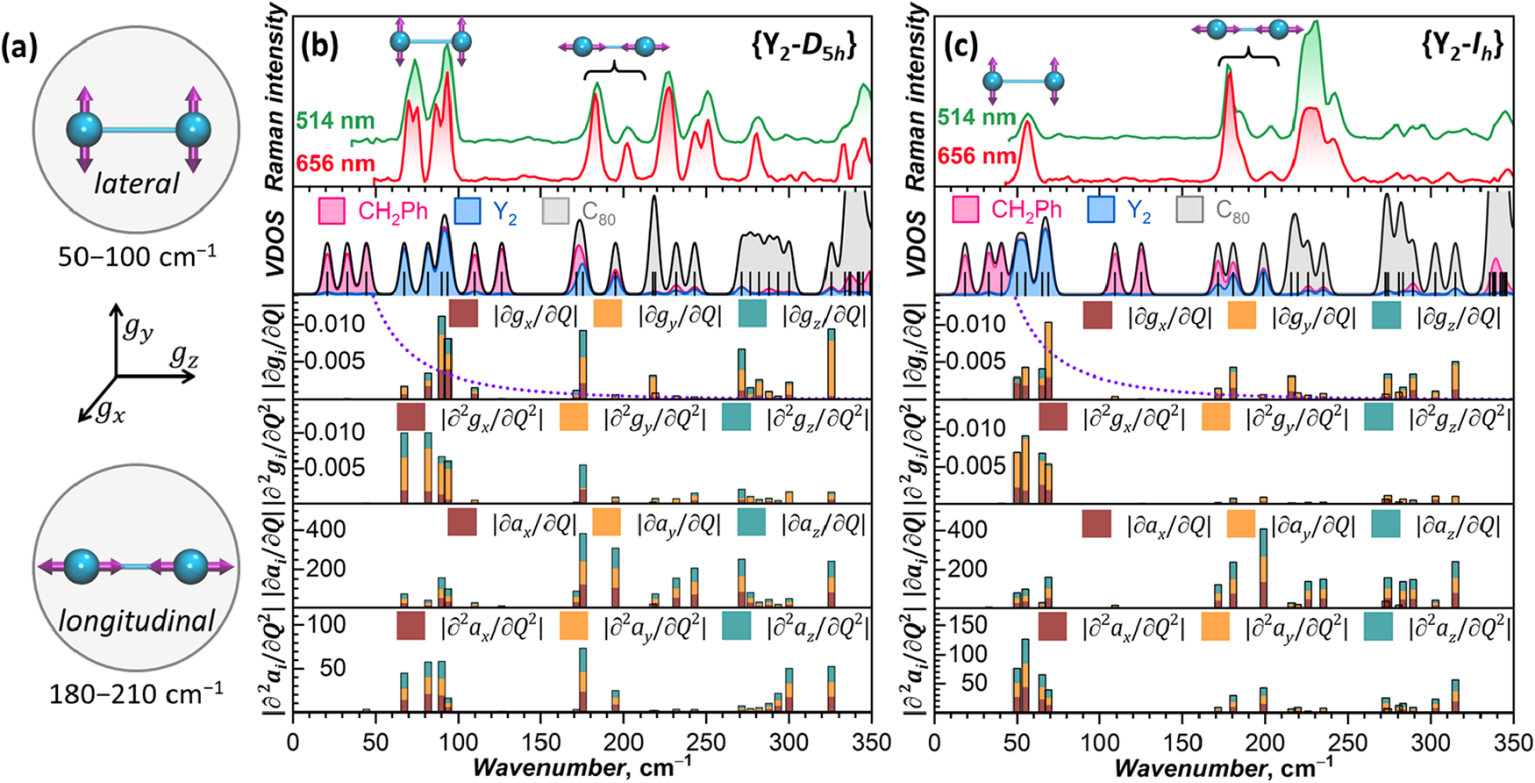
(a) Schematic description of lateral and longitudinal metal modes and orientation of *g*-tensor principal axes in Y_2_@C_80_(CH_2_Ph). (b, c) Low-frequency part of the vibrational spectra of (b) {Y_2_-*I*_*h*_} and (c) {Y_2_-*D*_5*h*_}. Upper panels plot Raman spectra measured at 78 K using laser excitations at 656 and 514 nm; panels second from top show DFT-computed vibrational frequencies broadened by Gaussian with a peak width of 5 cm^–1^ (denoted as VDOS, vibrational density of states); coloration visualizes contributions of structural fragments to the VDOS (benzyl group: pink; Y atoms: blue; carbon cage: gray); four lowest panels show the sum of the first and second derivatives of *g*-tensor and *A*-tensor components with respect to normal coordinates, |∂*g*_*i*_/∂*Q*_*k*_| (in Å^–1^), |∂^2^ *g*_*i*_/∂*Q*_*k*_^2^| (in Å^–2^), |∂*a*_1,2*i*_/∂*Q*_*k*_| (in MHz Å^–1^), and |∂^2^ *a*_1,2*i*_/∂*Q*_*k*_^2^| (in MHz Å^–2^) for each vibrational mode (*i* = *x*, *y*, *z*; indices 1 and 2 label Y atoms); contributions of individual Cartesian components are plotted with different colors. Dotted violet curves demonstrate how the thermal factor exp (*hν*/*k*_B_ *T*) {exp­(*hν*/*k*_B_ *T*) – 1}^−2^ changes across the vibrational spectrum at 100 K.

Experimentally, we studied vibrational spectra by Raman spectroscopy at 78 K (Figure 7b,c). Two excitation lasers at 514 and 656 nm were used to account for possible resonance effects and to ensure that the peaks detected at low frequencies close to the excitation laser line are not measurement artifacts. The symmetric longitudinal mode in dimetallofullerenes usually has medium-strong Raman intensity and a narrow line width^52,55,59,137^ and can be identified at 181 cm^–1^ in {Y_2_-*I*_*h*_} and 184 cm^–1^ in {Y_2_-*D*_5*h*_}, while the antisymmetric counterparts show lower intensity and occur at 205 and 203 cm^–1^, respectively. Lateral modes are harder to detect, as their low frequencies are often below the measurable range. The frequency limit of ∼40 cm^–1^ in our Raman measurements allows clear detection of the lateral modes in {Y_2_-*D*_5*h*_} as two strong doublet peaks at 71/75 and 88/94 cm^–1^ (Figure 7b). For {Y_2_-*I*_*h*_}, only one relatively broad feature at 56 cm^–1^ is found below 100 cm^–1^ (Figure 7b). At this moment, it is not clear whether this peak encompasses all lateral modes of {Y_2_-*I*_*h*_}, or if there might be another band below 40 cm^–1^, outside our detection limit. In either case, the lateral modes of the two isomers have distinctly different frequencies, which is in good agreement with the calculation results. Also, note that a very similar difference in the metal-based modes of the *I*_*h*_ and *D*_5*h*_ isomers was recently found in the Raman spectra of Nd_2_@C_80_(CF_3_).^59^ Unlike the metal-based modes, vibrations of the benzyl groups between 100 and 150 cm^–1^ do not exhibit detectable Raman activity. The fullerene cage modes appear above 200 cm^–1^ and agree well with the theoretical prediction for the squashing modes below 250 cm^–1^, while the comparison at higher frequencies is less straightforward due to the dramatically increased density of vibrational states and the strongly varied Raman intensity.

Both computational and experimental studies show that the effect of the cage isomerism on the lateral-mode frequencies is much more pronounced than for longitudinal modes. This can be explained by the nature of the metal–fullerene bonding in EMFs, which is usually not strongly directional but rather corresponds to the interaction of a metal atom with a certain region of the π-electron density, typically comprising 10–12 atoms.^138^ In the lateral modes, metal atoms slide parallel to the fullerene π-system, and the potential energy surface for such a motion is naturally sensitive to the π-system topology, which is different in two isomers. In longitudinal modes, metal atoms move perpendicular to the fullerene surface and their frequencies represent the net strength of the metal–carbon bonding. As we found earlier, the net metal-cage bonding corresponds to the formal valence of the metal and does not vary much with the isomeric structure of the fullerene cage.^138^

#### Lateral Modes and Spin-Lattice Relaxation

Having ensured that DFT provides a good description of low-frequency molecular vibrations in {Y_2_-*I*_*h*_} and {Y_2_-*D*_5*h*_} and most importantly correctly reproduces the difference in lateral-mode frequencies between the two isomers, we can proceed with the analysis of their role in spin-lattice relaxation. Vibrations relax spins in radicals with *S* = 1/2 via single-phonon direct and two-phonon Raman mechanisms. The direct mechanism implies relaxation via acoustic phonons with a frequency equal to Zeeman splitting Δ*E*_Zee_. This mechanism is dominant only at very low temperatures, when thermal populations of optical modes are negligible, and is not expected to have a strong contribution for Y_2_@C_80_(CH_2_Ph) radicals above 20 K even for W-band measurements (Δ*E*_Zee_ = 3.1 cm^–1^).^124^ Relaxation under the Raman mechanism can be presented as a sum of contributions from individual vibrational modes:

4 $$T_{1}^{-1}∼\Sigma_{k}V_{\mathrm{sp}-\mathrm{ph}}^{\left(k\right)}\exp\left(h\nu_{k}/k_{B}T\right){\left\{\exp\left(h\nu_{k}/k_{B}T\right)-1\right\}}^{-2}$$

where ν_*k*_ and *V*_sp‑ph_^(*k*)^ are the vibrational frequency and spin-phonon coupling parameters of the *k*th mode. Equation 4 naturally emerges in the microscopic analysis of the spin-lattice relaxation in an open quantum system^139–141^ and resembles the classical local-mode mechanism,^142–144^ except that the latter is often reduced to a contribution of a single vibrational mode. *V*_sp‑ph_^(*k*)^ can be estimated through derivatives of the spin Hamiltonian with respect to normal-mode displacements and includes two terms emerging at different levels of perturbation theory, with spin-coupling parameters of ∂^2^ *Ĥ*_spin_/∂*Q*_*k*_∂*Q*_*l*_ and (∂*Ĥ*_spin_/∂*Q*_*k*_)­(∂*Ĥ*_spin_/∂*Q*_*l*_) forms.^145^ The latter term corresponds to relaxation via excited states and has excitation energy in the denominator. It should not be present for radicals with *S* = 1/2 as their spin Hamiltonian has no higher-energy excited states, but recent analysis demonstrated that this term can become substantial or even dominant when the total electronic Hamiltonian is considered instead of the effective spin Hamiltonian.^134,146,147^ As we do not seek here exact predictions of relaxation times but rather wish to establish general trends, we abstained from expensive calculations of mixed partials and electronic excitations. Given the small Zeeman splitting of the spin-up and spin-down energy levels for *S* = 1/2 and the condition on the mode frequencies *|h*ν_*k*_ – *h*ν_*l*_ *|* = Δ*E*_Zee_, the main contribution for molecular vibrations corresponds to *k* = *l*, with the small-energy mismatch covered by frequency dispersions in the *k*-space. On these premises, the importance of a given vibrational mode for Raman relaxation can be evaluated based on the first- and second-order derivatives without considering mixed partials. Furthermore, *V*_sp‑ph_^(*k*)^ = (∂*Ĥ*_spin_/∂*Q*_*k*_)^2^ can then serve as the first approximation for the qualitative analysis,^133,139,148^ although accurate prediction would require a more elaborate treatment of the spin-phonon coupling.

Earlier studies of organic radicals and metal complexes and our results on Y_2_@C_80_(CH_2_Ph) show that *T*_1_ values do not seem to strongly depend on *m*_*I*_, suggesting that the *g*-tensor modulation is the main reason for spin-lattice relaxation.^128–130,134,149^ On the other hand, spin-dynamics simulations performed by Lunghi revealed that vibrational modulation of hyperfine constants caused a faster relaxation than the Zeeman term.^141^ Thus, we assume that derivatives of both the *g*-tensor and *A*-tensor components can be relevant. For each isomer, we computed these derivatives at the PBE-ZORA/def2-TZVP level by numerical differentiation of *g*- and *A*-tensors with respect to atomic displacements of 23 lowest-frequency vibrational modes (see Figure S16 for a representative example and Table S3 for computed derivatives). Absolute values of ∂*g*_*i*_/∂*Q*_*k*_ and ∂*a*_*i*_/∂*Q*_*k*_ summed up over three principal Cartesian components (*i* = *x*, *y*, *z*) and analogous values for second-order derivatives are compared with vibrational spectra in Figure 7b,c. In essence, ∂*Ĥ*_spin_/∂*Q_k_* derivatives, known also as spin-phonon coupling parameters, demonstrate how strongly spin Hamiltonian parameters (such as components of the *g*-tensor and *A*-tensor) change when the molecule is distorted by a vibration with the normal coordinate *Q_k_* (Figure S16). Vibrations, introducing larger variations to spin Hamiltonian parameters, should be more efficient in spin-lattice relaxation.

Figure 7 shows that modes of different types can induce a comparable modulation of *g-* and *A*-tensors. For instance, lateral, longitudinal, and some predominantly cage modes have similarly large ∂*g*_*i*_/∂*Q*_*k*_ derivatives in the {Y_2_-*D*_5*h*_} isomer. In the {Y_2_-*I*_*h*_} isomer, ∂*g*_*i*_/∂*Q*_*k*_ derivatives of lateral modes are of similar size to those of {Y_2_-*D*_5*h*_} counterparts, whereas *g*-tensor derivatives for other vibrations are on average smaller. Among the lateral modes of both isomers, those with an enhanced translational character give larger ∂*g*_*i*_/∂*Q*_*k*_ values. Hyperfine derivatives exhibit a somewhat different pattern. For them, lateral modes are less active than longitudinal and cage modes, while the latter two types give similar ∂*a*_*i*_/∂*Q*_*k*_ values for both isomers. Interestingly, although the contribution of metal atoms to a given vibration appears important for enhancing its spin-vibronic activity, it does not directly translate into the size of the derivative. Thus, some cage modes with ∼10% metal contribution show similarly large ∂*g*_*i*_/∂*Q*_*k*_ derivatives as lateral modes with ∼90% metal contribution. Vibrations of the exohedral benzyl group do not exhibit noticeable spin-vibronic activity for both the first and second derivatives, which is not surprising, given that these vibrations do not change the electronic and geometrical structures of the Y_2_@C_80_ moieties.

Analysis of vibrational contributions to spin-lattice relaxation cannot be complete without considering the thermal weighing, since relaxation requires some phonon population. In fact, including the thermal weight from eq 4 changes the picture quite dramatically because of its very fast decay with increasing frequency (Figure 7b,c). For instance, compared to its value computed at 50 cm^–1^ and 100 K, the exp (*hν*/*k*_B_ *T*) {exp­(*hν*/*k*_B_ *T*) – 1}^−2^ value decreases by the factor of 4.5 at 100 cm^–1^ and 29 at 200 cm^–1^. This scaling essentially allows one to eliminate cage modes from the analysis. Three lowest-frequency benzyl vibrations have favorable temperature scaling, but their ∂*Ĥ*_spin_/∂*Q*_*k*_ derivatives are still too small to produce a significant contribution. Substitution of *V*_sp‑ph_^(*k*)^ = (∂*Ĥ*_spin_/∂*Q*_*k*_)^2^ into eq 4 shows that only lateral modes remain relevant for the relaxation driven by the vibrational modulation of the *g*-tensor, and only lateral and longitudinal modes contribute to the relaxation via the hyperfine term (Figure S17). In both situations, summation in eq 4 demonstrates that because of the lower frequencies of its lateral modes (Figure S17), *T*_1_ values of {Y_2_-*I*_*h*_} should be at least twice shorter than those of {Y_2_-*D*_5*h*_}, which agrees with experimental results (Table 3).

This section can be narrowed down to the statement that the lateral modes play a crucial role in the spin-lattice relaxation of the EMF radicals. In hindsight, this conclusion does not even require calculations of spin-phonon couplings. It suffices that, on the one hand, these modes occur at low frequencies and often are the lowest-frequency optical modes of a given EMF molecule and, on the other hand, they directly involve metal atom(s), on which the spin is localized. We earlier observed that relaxation of magnetization in lanthanide-EMF single-molecule magnets sometimes show an Arrhenius regime with an effective barrier of 20–40 cm^–1^, suggesting the local-mode mechanism with involvement of lateral modes as no other likely candidate modes exist in this frequency range.^56,150,151^ More recently, dominant contributions of these modes to spin-lattice relaxation were predicted for some EMFs by *ab initio* calculations of spin-phonon couplings.^100,152,153^ One can also note that due to the shallow potential energy surface for the lateral motion, these modes are likely considerably anharmonic. Anharmonicity not only decreases the reliability of theoretical predictions of the frequencies but also allows phonon-phonon interactions, decreases phonon lifetimes, and accelerates spin-lattice relaxations. Furthermore, low-frequency vibrations of endohedral atoms and clusters efficiently mix with acoustic phonons, especially outside the Γ-point.^154^ Through this mixing, these modes can further mediate energy dissipation and spin-lattice relaxation via Raman and direct mechanisms. In a broader context, the mixing of low-frequency molecular vibrations with lattice phonons was demonstrated by inelastic neutron scattering^155^ and inelastic X-ray scattering studies,^156^ while DFT calculations and spin-dynamics simulations showed that such low-frequency modes of mixed nature play the main role in spin-lattice relaxation.

#### Spin-Phase-Memory Relaxation Times *T*_m_ of {Y_2_-*D*_5*h*_} and {Y_2_-*I_h_*}

Spin-memory relaxation times *T*_m_ of both isomers, 7–8 μs at 20 K and 0.3–0.4 μs at 105 K, are 1–2 orders of magnitude shorter than *T*_1_ (Table 3), indicating that decoherence is not limited by the spin-lattice relaxation. Remarkably, while the difference between the *T*_1_ values of the two isomers is quite considerable, their *T*_m_ values are very close. Analogous to *T*_1_, *T*_m_ also depends on the molecular orientation, but in a different manner (Figure 5). The shortest *T*_m_ values are found in the middle of the spectrum, in the intermediate region between *z* and *xy* domains, while the longest values are measured at the spectral edges, where the magnetic field is parallel to the *z* or *x* axes. For example, at 20 K, the *T*_m_ of {Y_2_-*D*_5*h*_} increases gradually from 6 μs in the middle to 11–12 μs at the edges (Figure 5d). Unlike *T*_1_, *T*_m_ does not exhibit a pronounced difference between *z* and *xy* values, and in Table 3 and Figure 6a we list two sets of *T*_m_ values: *T*_m,mid_ averaged over the middle range and *T*_m,*xyz*_ averaged over *z* and *xy* domains.

Within *z* and *xy* domains, *T*_m_ exhibits noticeable variations near the principal orientations, especially when the temperature is increased above 50 K. Qualitatively, these variations can be established already from the shape of the ESE spectra, as illustrated in Figure 8 for the {Y_2_-*D*_5*h*_} isomer in the high-field range, corresponding to the *xy* domain. At 20 K, the ESE spectrum resembles CW powder spectra, with the rightmost turning points producing a step-like spectral shape, which parallels the angular distribution of molecular orientations. A similar spectrum is measured at 50 K, but at 79 K the step-edges start to sharpen, indicating that *T*_m_ close to the principal axes is longer than in other orientations. This tendency becomes full-fledged at 105 K, as not only the sharp peaks develop instead of steps at this temperature but also the *T*_m_ values at intermediate fields between *z* and *xy* orientations become shorter than the measurable threshold, giving no detectable spin echo. The trend is further substantiated by sawtooth patterns shown by *T*_m_ values measured at 79 and 105 K at fields alternating between the ESE peaks and the valleys between them (Figure 8). Conversely, the changes in *T*_m_ with the field at 20 K are more gradual.

**Figure 8:**
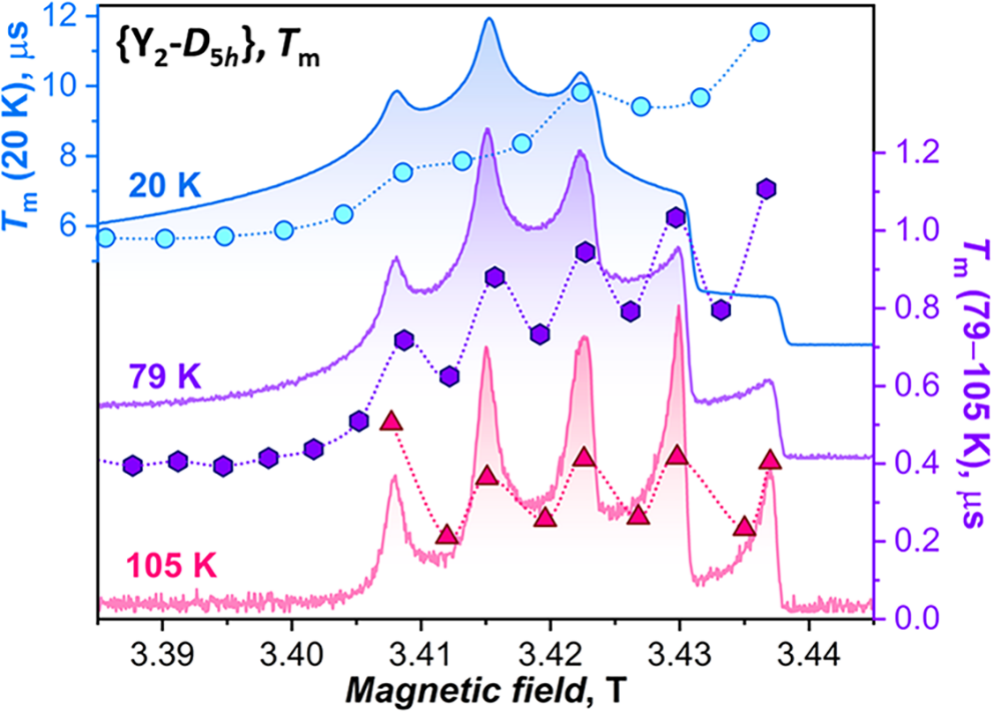
ESE-W-band spectra of {Y_2_-*D*_5*h*_} measured at 20, 79, and 105 K in the field range where resonance conditions correspond to *B*_0_∥*x*,*y* (referred to as the *xy* domain); dots are *T*_m_ measured at these temperatures in different magnetic fields, and connecting lines are shown to only guide the eye.

Such orientational dependence of *T*_m_ is characteristic for the spin decoherence caused by the rotational motion.^126,127,129,130,132,157–159^ Reorientation of excited radicals on the time scale of ESE measurement brings them out of the resonance and hence contributes to spin dephasing. Owing to the shape of the angular distribution with turning points at principal orientations, the molecules oriented along the *x*, *y*, or *z* axes are the least susceptible to this dephasing mechanism and show the longest *T*_m_ values. Furthermore, only the turning points at the highest and lowest fields have no contribution of molecules with intermediate orientations, while other turning points inevitably include a fraction of “misaligned” molecules, which increases on going from the edges of the spectrum to its center. Accordingly, the longest *T*_m_ values can be expected at the very edges, and the times should shorten on moving to the center of the spectrum. Our observations at 20 and 79 K nicely follow these expectations. At 105 K, *T*_m_ values at peak positions appear equalized, which can be tentatively explained by the fast relaxation of molecules with intermediate orientations, which therefore do not contribute to the ESE and thus to *T*_m_.

Given the location of the unpaired electron in the Y_2_@C_80_(CH_2_Ph) radicals on the metal dimer, the reorientation dephasing may be caused by the rotation or libration of the Y_2_ unit inside the fullerene cage or by the analogous motions of the fullerene molecule on the whole. In view of the strong difference in the internal dynamics of Y_2_ in {Y_2_-*D*_5*h*_} and {Y_2_-*I*_*h*_} (see Figure 3 and the discussion of lateral modes above), and very similar *T*_m_ measured for the two isomers, tumbling of the whole fullerene molecules appears to be a more decisive factor. Note that the effect is strong at 79 K and above, when the temperature approaches glass transition in toluene at 117 K, thus softening the matrix and facilitating the tumbling.

#### Comparison with Other Dimetallofullerenes with *S* = 1/2

Relaxation times of {Y_2_-*D*_5*h*_} and {Y_2_-*I*_*h*_} can be compared with those of other di-EMFs with a single-electron metal–metal bond and *S* = 1/2, such as Y_2_@C_79_N and its supramolecular complex Y_2_@C_79_N⊂[4]­CHBC,^70^ mixed-metal complexes CaY@*C*_*s*_(6)-C_82_ ^73^ and CaSc@*C*_*s*_(6)-C_82_,^74^ and Sc_2_@*I*_*h*_-C_80_(CH_2_Ph)^54^ (aka {Sc_2_-*I*_*h*_}); their *T*_1_ and *T*_m_ values for selected temperatures are listed in Table S4. Although measurement conditions, such as solvents or microwave frequency used, are not identical in different groups, certain trends can be revealed. At around 20 K, Y_2_@C_79_N and Y_2_@C_80_(CH_2_Ph) have comparable *T*_1_, which are somewhat shorter than that in CaY@C_82_. By approaching 100 K, *T*_1_ of CaY@C_82_ and {Y_2_-*D*_5*h*_} becomes similar, whereas {Y_2_-*I*_*h*_} relaxes much faster. Analysis of isostructural pairs CaY@C_82_/CaSc@C_82_ and {Y_2_-*I*_*h*_}/{Sc_2_-*I*_*h*_} shows that *T*_1_ of Sc analogues are several times longer. The main reason is presumably the lower mass of Sc, which shifts lateral metal modes to higher frequencies.

*T*_m_ do not follow the same trend as *T*_1_. All di-EMFs demonstrate nearly constant *T*_m_ values below 40 K and even shortening at 10 K, which is the known signature of the tunneling relaxation, often caused by the CH_3_ rotation in toluene. *T*_m_ values below 50 K are typically near 10–20 μs irrespective of the di-EMF type, Y_2_@C_79_N and {Sc_2_-*I*_*h*_} showing the longest values. Above 50 K, all *T*_m_ values decrease to 1–2 μs or below. As we proposed above, rotational motion is one of the possible factors limiting *T*_m_. Another plausible factor is nuclear spin flip-flops of the solvent and the benzyl group.

#### *T*_1_ and *T*_m_ of {YGd-*I*_*h*_} and {Gd_2_-*I*_*h*_}

Substitution of one or two Y atoms with Gd results in much faster spin-lattice relaxation in {YGd-*I*_*h*_} and {Gd_2_-*I*_*h*_} (Figures 6 and 9). *T*_1_ of {YGd-*I*_*h*_} measured at 3.50 T decays from 10 μs at 6 K to 2 μs at 20 K, while *T*_1_ of {Gd_2_-*I*_*h*_} equals 1.8 μs already at 14 K, which is the highest temperature at which ESE is measurable for this compound in *d*_8_-toluene. Likewise, *T*_m_ values of ∼2 μs at 6 K decrease below 0.8 μs at 12 K for {Gd_2_-*I*_*h*_} and at 20 K for {YGd-*I*_*h*_}. The ratio of *T*_1_ to *T*_m_ suggests that the spin decoherence above 10 K is limited by spin-lattice relaxation. *T*_1_ and *T*_m_ of {YGd-*I*_*h*_} are slightly longer than in {Gd_2_-*I*_*h*_}, but this difference is smaller than field variations of these values and is not substantial when compared to {Y_2_-*I*_*h*_} (Figure 6), and definitively does not scale with the number of Gd atoms in the metal dimer. Relaxation times of {YGd-*I*_*h*_} and {Gd_2_-*I*_*h*_} are compared to those of other metallofullerenes with the 4f^7^ shell in Table S5.

**Figure 9:**
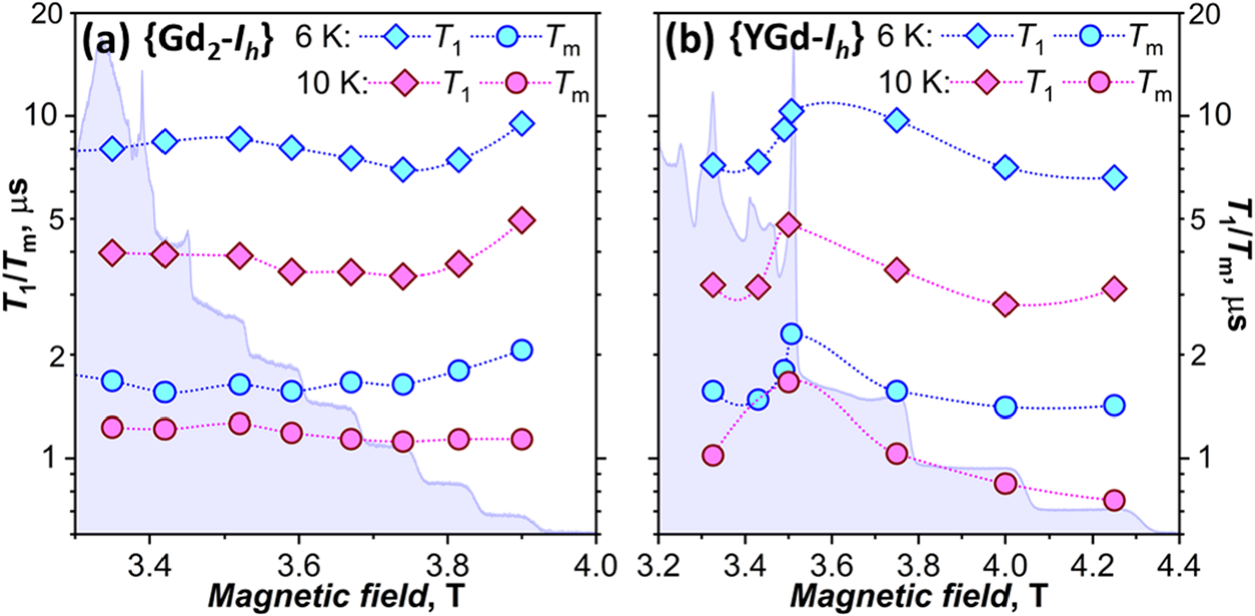
*T*_1_ and *T*_m_ of (a) {Gd_2_-*I*_*h*_} and (b) {GdY-*I*_h_} measured at 6 and 10 K; lines connecting points are plotted to guide the eye only. ESE spectra at 6 K are shown in the background.

Orientational dependence in the EPR spectrum for *S* >1/2 is more complex since the spectrum encompasses several |*m*_*S*_⟩ → |*m*_*S*_ + 1⟩ transitions, each with its own angular distribution determined by the size of *m*_S_, *D*, *E*, and *g*-tensor anisotropy (Figure 4). Therefore, transitions with different *m*_*S*_ values for molecules in different orientations will largely overlap, making it hardly possible to disentangle orientational dependence. Only the |−15/2⟩ → |−13/2⟩ transition for {Gd_2_-*I*_*h*_} and the |−4⟩ → |−3⟩ transition for {YGd-*I*_*h*_}, as the ones with the broadest angular distribution, will have certain field ranges at the edges of the spectrum, in which they do not overlap with other transitions. Six K *T*_1_ measurements for {Gd_2_-*I*_*h*_} at 2.98 T (*B*_0_∥*x*) and 3.90 T (*B*_0_∥*z*) gave the values of 12.9 and 9.5 μs, respectively. The *T*_1_ anisotropy is thus noticeable but considerably smaller than that for {Y_2_-*I*_*h*_} and shows the opposite trend. Besides, different relaxation times at 2.98 and 3.90 T may be caused not only by different molecular orientations but also by the overall field dependence of *T*_1_. At the same time, *T*_m_ values in these two fields are both equal to 2.1 μs.

To understand how *T*_1_ and *T*_m_ depend on *m*_*S*_, the measurements were performed in fields near turning points, corresponding to *B*_0_∥*z* for different |*m*_*S*_⟩ → |*m*_*S*_ + 1⟩ transitions (Figure 9). For {Gd_2_-*I*_*h*_} at 6 K, the longest *T*_1,*z*_ is the aforementioned 9.5 μs for |−15/2⟩ → |−13/2⟩ at 3.90 T, the shortest one is 7.0 μs for |−11/2⟩ →|−9/2⟩ at 3.74 T, while the average *T*_1,*z*_ is ∼8 μs. A similar pattern but with twice shorter times is found at 10 K. The picture is somewhat distorted by the overlap with transitions of molecules, whose orientations deviate from *B*_0_∥*z*, but the overall conclusion about the moderate variation of *T*_1,*z*_ with *m*_*S*_ should remain valid. Furthermore, very similar times are measured at 3.35 T, the field in which *B*_0_∥*y* is observed for most of the transitions (but not for |−1/2⟩ → |1/2⟩, which gives a separate sharp peak at 3.39 T). Finally, the *T*_m_ of {Gd_2_-*I*_*h*_} also does not show a discernible variation with *m*_*S*_.

A more pronounced *m*_*S*_-dependence is found for {YGd-*I*_*h*_}. Here, *T*_1,*z*_ increases opposite to |*m*_*S*_|, from the shortest value of 6.6 μs for the |−4⟩ → |−3⟩ transition at 4.25 T to the longest one of 10.3 μs for the |−1⟩ → |0⟩ transition at 3.50 T. The enhanced *T*_1_ for |−1⟩ → |0⟩ is found only in the z orientation, whereas *T*_1,*xy*_ for the same transition measured at 3.33 T is reduced to 7.2 μs. Similar *T*_1,*xy*_ of 7.3 μs is determined for the |0⟩ → |1⟩ transition at 3.43 T. Unfortunately, because of the strong overlap with other transitions, it is not possible to determine *T*_1,*z*_ for |0⟩ → |1⟩. The 50% enhancement of *T*_1,*z*_ for |−1⟩ → |0⟩ is also observed at 10 K, and it is paralleled by longer *T*_m_ values measured at 3.50 T when compared to other fields (Figure 9).

All arguments about the role of molecular vibrations in spin-lattice relaxation of Y_2_@C_80_(CH_2_Ph) remain valid for Gd-containing analogues. Furthermore, Gd is almost twice as heavy as Y, which shifts metal-based modes to lower vibrational frequencies (see Figures S14 and S15). However, this frequency shift alone is not sufficient to accelerate the spin-lattice relaxation rate of {Gd_2_-*I*_*h*_} and {YGd-*I*_*h*_} by nearly 2 orders of magnitude when compared to {Y_2_-*I*_*h*_}. As the former have additional ZFS terms in spin Hamiltonian (eq 3), it is reasonable to assume that vibrational modulation of ZFS leads to much faster relaxation. In other words, relaxation is mainly caused by electron-electron interactions. Since DFT is not able to provide a reasonable description of *D* and *E* parameters in the [Gd^3+^–e–Gd^3+^] system,^160^ while CASSCF calculations are much more computationally expensive and are still of limited accuracy for this problem, we abstain from numerical evaluation of ∂*D*/∂*Q*_*k*_ and ∂*E*/∂*Q*_*k*_ derivatives in this work and limit the discussion to qualitative arguments only. It is very plausible that lateral modes should be equally crucial for ZFS-modulated spin-lattice relaxation, as had been found for Eu^II^-based monometallofullerenes.^100^

{YGd-*I*_*h*_} has four times larger *D* and twice larger *E* parameters than {Gd_2_-*I*_*h*_} (Table 2), and it is likely that its ∂*D*/∂*Q*_*k*_ and ∂*E*/∂*Q*_*k*_ derivatives will also be larger on average. However, ZFS terms in spin Hamiltonian have *Ŝ*_*i*_^2^-derived multipliers, which will be smaller for {YGd-*I*_*h*_} due to its nearly twice smaller spin. Apparently, these two factors compensate for each other, leading to similar spin-lattice relaxation times of {YGd-*I*_*h*_} and {Gd_2_-*I*_*h*_}. Nonetheless, a larger anisotropy of {YGd-*I*_*h*_} is a plausible reason for its more pronounced *T*_1,*z*_ dependence on *m*_*S*_, because for molecules with *B*_0_∥*z* orientation, the relaxation driven by the modulation of ZFS is determined by *V*_sp‑ph_^(*k*)^ ∼ (∂*D*/∂*Q*_*k*_)^2^ *S*_*z*_^4^, hence the elongation of *T*_1,*z*_ with the decrease of *S*_*z*_ and anomalously long relaxation for the |−1⟩ → |0⟩ transition in *z* orientation. The longer relaxation might be expected for the |−1/2⟩ → |1/2⟩ transition in {Gd_2_-*I*_*h*_} since ZFS enters relaxation only in the second order, but the thermal population of the |−1/2⟩ state remains small below 20 K. Its sharp feature is thus visible in the spectrum, but the density of other transitions at the same field is too high. The prolonged relaxation for the |−1/2⟩ → |1/2⟩ transition in {Gd_2_-*I*_*h*_} can be ascertained from the room-temperature spectrum in liquid solution, in which only one line caused by this transition was observed.^55^

#### Rabi Oscillations

In considering electron spins in molecules as qubit candidates, the main effort is usually devoted to the prolongation of *T*_m_. However, the next step in the realization of quantum information processing based on such qubits will be the design of supramolecular architectures allowing entanglement of a larger number of qubits. In this respect, systems with multiple spin levels, such as those provided by hyperfine interactions or for *S* > 1/2, can be advantageous despite the reduced coherence time usually associated with spin-spin interactions, because increasing the size of the Hilbert space allows encoding of more than one qubit in one molecule. The demonstration of quantum algorithms is beyond the scope of this work, and here we focus only on the first prerequisite for such operations, the ability of the system to maintain entanglement. This can be ascertained through the study of Rabi oscillations, which were performed for {Y_2_-*D*_5*h*_}, {Gd_2_-*I*_*h*_}, and {YGd-*I*_*h*_}. Figure 10 shows that oscillations with the linear dependence of the nutation frequency on the B_1_ field, as required by the Rabi formula, were observed for all studied molecules.

**Figure 10:**
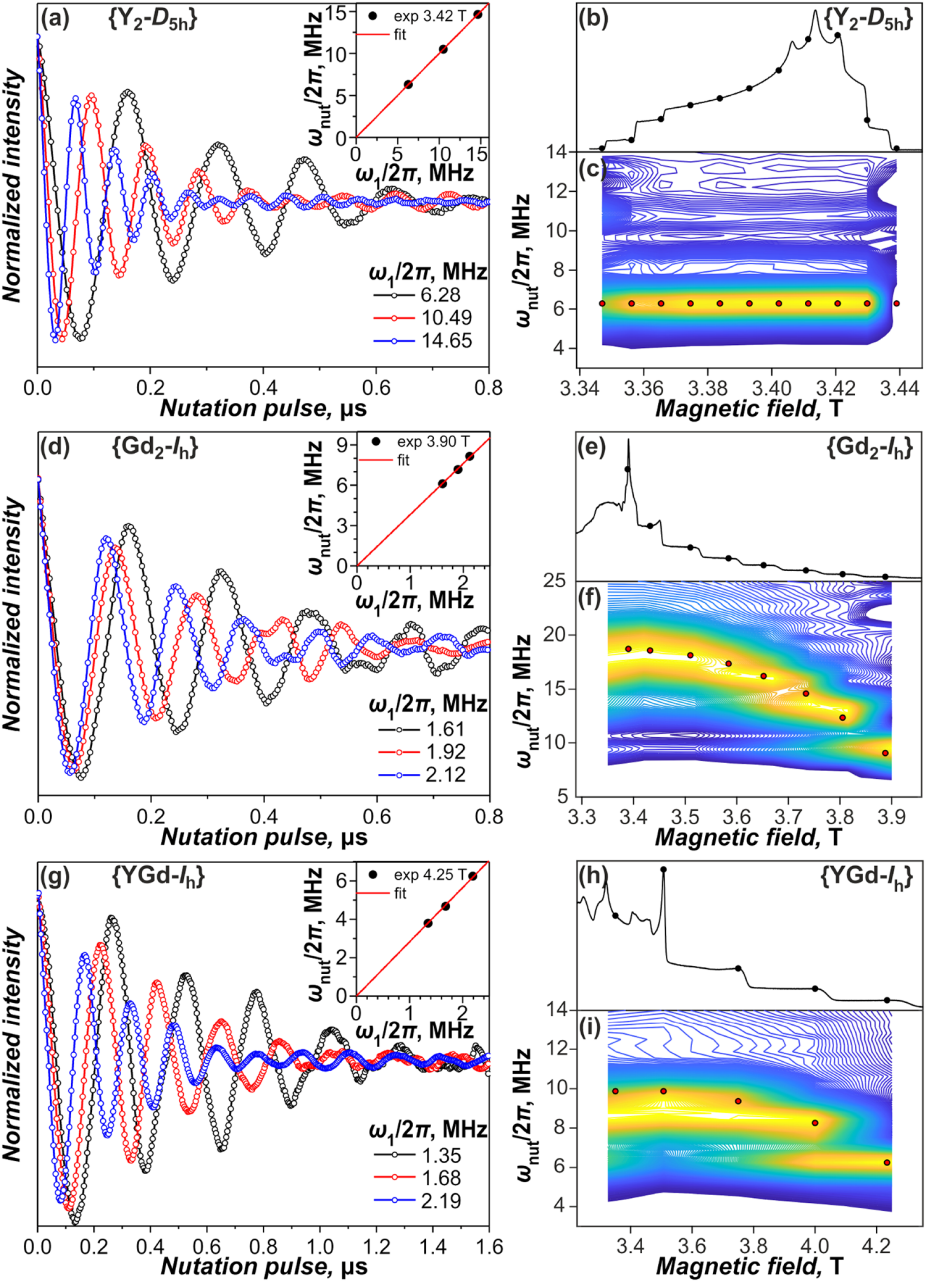
(a, d, g) Rabi oscillations of {Y_2_-*D*_5*h*_} (a, *B*_0_ = 3.42 T; *T* = 20 K), {Gd_2_-*I*_*h*_} (d, *B*_0_ = 3.90 T; *T* = 10 K), and {YGd-*I*_*h*_} (g, *B*_0_ = 4.25 T; *T* = 6 K) at three different values of *B*_1_ field (ω_1_/2π = *g*β_*e*_ *B*_1_/*h* represents the *B*_1_ field in linear frequency units). Insets show the linear dependence of nutation frequency on *B*_1_. (b, e, h) ESE-W-band EPR spectra of {Y_2_-*D*_5*h*_}, {Gd_2_-*I*_*h*_}, and {YGd-*I*_*h*_}, indicating magnetic fields, at which Rabi oscillations were measured. (c, f, i) Fourier transformed Rabi oscillations measured at different fields and plotted as 2D maps in coordinates of nutation frequency and magnetic field (ω_1_/2π = 6.28 MHz for {Y_2_-*D*_5*h*_}, 2.12 MHz for {Gd_2_-*I*_*h*_}, and 2.19 MHz for {YGd-*I*_*h*_}). Individual Fourier transformed spectra can be found in Figure S18. Dots mark the positions of Rabi frequencies calculated as $\omega_{\mathrm{nut}}=\omega_{1}\sqrt{S\left(S+1\right)-m_{s}\left(m_{s}+1\right)}$ .

The measurements at different fields were performed to analyze if the frequencies depend on the orientation and type of EPR transitions. For {Y_2_-*D*_5*h*_}, no variation of the Rabi frequency was found across the whole ESE spectrum (Figure 10b,c). Evidently, *g*-tensor anisotropy and hyperfine interactions for this *S* = 1/2 system do not produce sufficient differences in transition matrix elements to give measurable deviation in the Rabi frequency for different molecular orientations. Quite a different situation is found for {Gd_2_-*I*_*h*_} and {YGd-*I*_*h*_}. Here, the measurements in different fields excite different |*m*_*S*_⟩ → |*m*_*S*_ + 1⟩ transitions, whose Rabi frequency in the first approximation are expected to vary as

5 $$\omega_{\mathrm{nut}}=\omega_{1}\sqrt{S\left(S+1\right)-m_{S}\left(m_{S}+1\right)}$$

where ω_1_ represents the *B*_1_ field in frequency units and is given by ω_1_ = 2*πg*β_*e*_ *B*_1_/*h*. Indeed, we observe a noticeable field dependence of the Rabi frequencies in both Gd-containing EMFs. A single peak appears in the FT spectrum only when excited at the high-field edge of the ESE spectrum, while the number of FT peaks increases at smaller fields, when more and more |*m*_*S*_⟩ → |*m*_*S*_ + 1⟩ transitions are excited at the same time (Figure S18). Peak maxima roughly follow eq 5. The deviations are caused by the overlap of several transitions with different Rabi frequencies as well as the approximate nature of eq 5, since *m*_*S*_ is not a good quantum number for spin systems with rhombic anisotropy and may require a more elaborate procedure for definition of ω_nut_.^65,76^

## Conclusions

In this work, we performed a pulsed EPR study of a family of dimetallofullerenes M_2_@C_80_(CH_2_Ph) with a single-electron M–M bond and a metal composition from Y_2_ through YGd to Gd_2_. The main questions addressed were how the fullerene isomerism and the total spin of encapsulated metal dimer affect the spin-lattice relaxation and spin decoherence. W-band EPR spectroscopy is crucial for the studies of orientational dependence and for interpretation of the spectra of Gd-EMFs, since the overlap of several |*m*_*S*_⟩ → |*m*_*S*_ + 1⟩ transitions in the latter severely complicate the spectra measured in lower magnetic fields.

The role of cage isomerism was revealed in a comparative study of Y_2_@*I*_*h*_-C_80_(CH_2_Ph) and Y_2_@*D*_5*h*_-C_80_(CH_2_Ph). The two isomers have different potential energy surfaces for the motion of the metal dimer, which is reflected in several dynamic phenomena. While Y_2_ rotates freely at room temperature in the *I*_*h*_ isomer, rotation inside the *D*_5*h*_ cage is hindered. The latter also features higher vibrational frequencies of lateral metal modes and eventually exhibits a longer spin-lattice relaxation. The direct connection between the lateral modes and the spin-lattice relaxation was established by calculations of spin-phonon couplings. Lateral metal modes are intrinsic to metallofullerenes and are destined to play a crucial role in the spin-lattice relaxation of EMFs simply by virtue of their low-vibrational frequencies and the direct involvement of metal atoms, ensuring large spin-phonon coupling. Metal-fullerene bonding in EMFs is usually not strongly directional but rather corresponds to the interaction of a metal atom with a certain region of π-electron density. Lateral metal vibrations are then nothing else than the sliding of metals along the surface of the said density, and their low frequencies are rooted in the relatively large atomic mass of metals and the flat potential energy surface (PES) for the motion parallel to the fullerene inner surface. Increasing their frequencies is the obvious strategy to prolong the relaxation times of EMFs, and since the metal atomic mass is the invariable constant, it is the curvature of the PES stemming from the π-system topology that should be dealt with. Fullerene isomerism is one way to tackle this problem, as exemplified in this work by *I*_*h*_ and *D*_5*h*_ cage isomers of Y_2_@C_80_(CH_2_Ph) or by different metal-cage binding sites studied in the Eu@C_2*n*_ family in ref 100. Another approach might be a chemical modification of the fullerene surface, as addition of exohedral groups changes the fullerene π-system and can lead to more localized metal-cage bonding. This approach may have an adversary side effect by introducing new low-frequency modes of exohedral groups, but our calculations for Y_2_@C_80_(CH_2_Ph) demonstrate that the spin-phonon coupling for such vibrations are very small. As to the spin decoherence, for Y_2_@C_80_(CH_2_Ph) in frozen toluene, it seems to be limited by the rotational motion of the molecules, which might be addressed by using more rigid and crystalline matrices. Exohedral modification can also hinder the rotational motion, suggesting that it is worth studying spin dynamics in chemical derivatives of di-EMFs.

Replacement of Y with Gd has the immediate effect of drastically shortening the relaxation times. We also did not observe a considerable difference in the *T*_1_ and *T*_m_ values of YGd@*I*_*h*_-C_80_(CH_2_Ph) and Gd_2_@*I*_*h*_-C_80_(CH_2_Ph), showing that acceleration of relaxation is not scaling with the number of heavier lanthanides. *D* and *E* parameters of heterometallic YGd are higher than those in symmetric Gd_2_, but as the total spin of the latter is almost twice as high, their overall zero-field splitting is similar. Studies of relaxation times for individual |*m*_*S*_⟩ → |*m*_*S*_ + 1⟩ transitions demonstrated a certain variation with *m*_*S*_, but the effect is not very pronounced. More importantly, YGd@C_80_(CH_2_Ph) and Gd_2_@C_80_(CH_2_Ph) EMFs show a distinct variation of Rabi frequencies with *m*_*S*_, which is not observed for hyperfine transitions of Y_2_@C_80_(CH_2_Ph).

## Supplementary Material



## Notes

Experimental and computational details, description of HPLC separation, additional EPR spectra, details of relaxation measurements, Raman spectra, computational results, and spin-phonon coupling parameters (PDF)
