# Supplementary material for: Orientationally Resolved Electron-Spin Relaxation and Coherence in Fullerene-Encapsulated Rare-Earth Dimers with a Single-Electron Metal–Metal Bond
Source: J Am Chem Soc. 2026 Feb 10;148(7):7857–75. doi: 10.1021/jacs.5c22495 (PMC12951446; doi:10.1021/jacs.5c22495)
Supplement: Supplementary file 1 [file ja5c22495_si_001.pdf]

Orientationally-resolved electron spin relaxation and coherence in fullerene-encapsulated rare-earth dimers with a single-electron metal-metal bond

Michal Zalibera[1]*, Lukas Spree[2,3], Fupin Liu[2,4], Marco Rosenkranz[2], Leonid Rapatskiy[5], Alexander Schnegg[5], Alexey A. Popov[2]*

[1] *Institute of Physical Chemistry and Chemical Physics, Slovak University of Technology, 81237 Bratislava, Slovakia*

[2] *Leibniz Institute for Solid State and Materials Research (IFW Dresden), 01069 Dresden, Germany*

[3] *Center for Quantum Nanoscience, Institute for Basic Science (IBS), 03760 Seoul, Republic of Korea*

[4] *School of Chemistry and Materials Science, Nanjing Normal University, Nanjing, 210023 China*

[5] *Max Planck Institute for Chemical Energy Conversion, 45470 Mülheim (Ruhr), Germany*

Supporting Information

Experimental and computational details	S2
Synthesis and separation (HPLC)	S4
Variable-temperature CW X-band EPR spectra	S6
EPR parameters of selected Y-centered radicals	S8
Spin parameters of selected Gd-based EMFs	S10
T_1 and T_m measurements	S11
Raman spectra	S14
DFT-optimized Cartesian coordinates	S18
Derivatives of g and A-tensors with respect to normal modes	S20
Relaxation times of selected EMFs	S27
Rabi oscillations	S29
References	S30

Experimental details

Synthesis and separation. The synthesis of $Y_2@I_h$-$C_{80}(CH_2Ph)$ and $Gd_2@I_h$-$C_{80}(CH_2Ph)$ was described in Refs. [1, 2] The graphite rods (length 100 mm, diameter 8 mm) are packed with metal oxides M_2O_3 mixed with graphite (molar ratio of M:C=1:15) and evaporated by direct-current arc discharge in Krätschmer-Huffman fullerene generator with the current of 100 A in 180 mbar helium atmosphere. The as-produced soot was Soxhlet-extracted by dimethylformamide (DMF) for 24 h, giving a mixture of fullerene anions in DMF solution. The fullerene anions were then reacted with excess of benzyl bromide under N_2 protection at elevated temperature (~100°C) for 20 hours to yield neutral air-stable benzyl monoadducts. After reaction, DMF was evaporated under reduced pressure, the residue (the mixture of fullerene benzyl adducts) was washed with methanol, then dissolved in toluene and subjected to high performance liquid chromatography (HPLC). For the synthesis of $YGd@I_h$-$C_{80}(CH_2Ph)$, the same procedure was performed using the mixture of Y_2O_3 and Gd_2O_3 in the arc-discharge evaporation. HPLC separation of $YGd@I_h$-$C_{80}(CH_2Ph)$ is described in Figure S1. HPLC separation of $Y_2@D_{5h}$-$C_{80}(CH_2Ph)$ is described in Figure S2.

Spectroscopic measurements. Matrix-assisted laser desorption/ionization time-of-flight (MALDI-TOF) ***mass-spectra*** were measured with a Bruker autoflex mass-spectrometer with 1,1,4,4-tetraphenyl-1,3-butadiene as a matrix. ***UV-vis-NIR absorption spectra*** were measured in toluene solution at room temperature with Shimadzu 3100 spectrophotometer. ***Raman spectra*** were recorded at 78 K on a T 64000 triple spectrometer (Jobin Yvon) using 514 nm line of the Ar gas laser and 656 nm excitation wavelength of the tunable dye laser Matisse 2 (Sirah Lasertechnik). Samples for Raman measurements were prepared by drop-casting toluene solutions on KBr single-crystalline substrate.

EPR measurements. Continuous-wave (CW) ***X-band*** EPR measurements were performed using a Bruker EMXmicro spectrometer. The samples were dissolved in toluene and transferred to standard 4 mm quartz EPR tubes. ***W-band*** measurements were performed at 5–110 K using a Bruker ELEXSYS E680 spectrometer operating at about 94 GHz. All experiments were carried out with a homebuilt EPR/ENDOR microwave cavity.[3-5] The samples were dissolved in d_8-toluene at concentration around 1 mg/mL, filled into a 0.7 mm (o.d.) quartz tube and flash frozen in liquid N_2 to ensure glass formation before inserting into He cryostat. Electron spin echo-detected (ESE) field-swept spectra were measured using the pulse sequence t_p–τ–$2t_p$–τ–echo with t_p = 16 ns and τ = 286 ns. Generally, 50 echos were accumulated and integrated over 140 ns around their maximum at each field position. The pulse repetition time was set to 1.7 ms. The same sequence was used to record the phase memory T_m decay kinetics by incrementing the initial interpulse delay t in 32–128 ns steps, depending on the echo decay rate. The inversion recovery traces were collected with a t_{inv}–T–t_p–$2t_p$–τ–echo sequence using t_{inv} = 80 ns, t_p = 16 ns, and τ = 286 ns and incrementing the T delay in steps of 0.2–7 µs (adjusted for each sample and temperature) starting from the initial value of T = 1 µs. Transient nutation experiments were performed with the t_{prep}–T–t_p–τ–$2t_p$–τ–echo sequence using t_{prep} = 4–1000 ns incremented in 4 ns steps, t_p = 16 ns, T = 3 µs and τ = 500 ns.
The EPR spectra were simulated by using the EasySpin toolbox[6] based on Matlab.

DFT calculations were performed using Orca suite.[7-9] Molecular structures and hessians of $Y_2@C_{80}(CH_2Ph)$ isomers were computed at the PBE/def2-TZVP level with Dolg's effective core potential for Y. Hyperfine coupling and g-tensors of $Y_2@C_{80}(CH_2Ph)$ isomers in optimized and distorted molecular geometries were computed at the PBE-ZORA level with ZORA-adjusted def2-TZVP basis set.[10, 11] Computations of spin density plotted in Figure 1c and broken-symmetry calculations of exchange interactions in $YGd@C_{80}(CH_2Ph)$ were performed at the PBE0 level with DKH scalar-relativistic correction.

DFT-based Born-Oppenheimer molecular dynamics simulations of $Y_2@C_{80}(CH_2Ph)$ isomers (Figure 3) were performed at the PBE/DZVP level using CP2K package[12, 13] and employed velocity Verlet algorithm with the time step of 0.5 fs and a Nosé–Hoover thermostat set at 300 K; total propagation time of trajectories was 60 ps. Molecular structures, isosurfaces and BOMD trajectories were visualized using the VMD package.[14]

HPLC separation of {YGd-I_h}

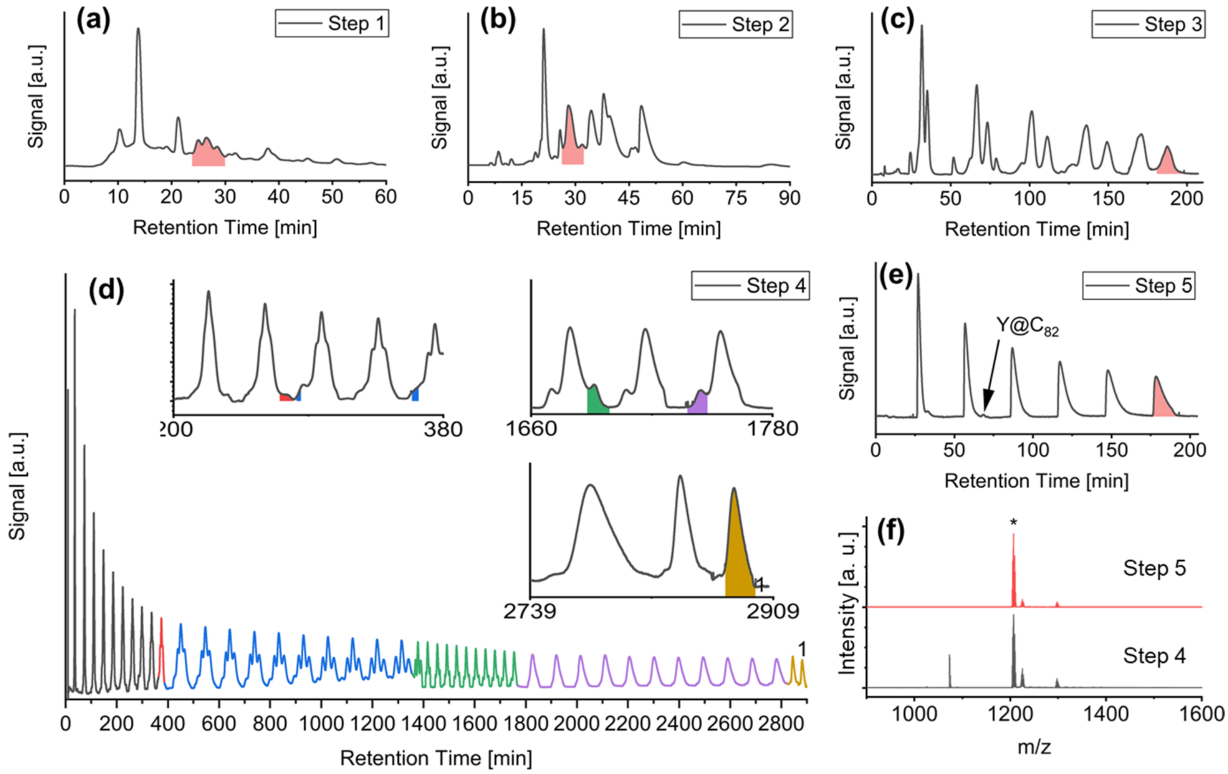

Figure S1. Separation of {YGd-I_h}. (a) Step 1, crude mixture of Y-Gd EMF derivatives after benzylation (toluene, 40°C, 5 mL/min, 2xBuckyprep semipreparative columns); fraction highlighted in pink is selected for the further separation. (b) Step 2 (toluene, 40°C, 5 mL/min, 2xBuckyprep-D semipreparative columns). (c) Step 3, recycling HPLC (Toluene, RT, 2.5 mL/min, Buckyprep semipreparative column); the fraction collected at this step mainly contains the mixture of {Y_2-I_h}, {YGd-I_h}, and {Gd_2-I_h}. (d) Step 4; recycling (Toluene, RT, 2.5 mL/min (black, red, and green), 1.0 mL/min (blue and purple), Buckyprep semipreparative column); the fraction collected in the end contained the target {YGd-I_h}, but also showed Y@C_{82} signal in mass-spectra. (e) Step 5, removal of Y@C_{82}(CH_2Ph) admixture, recycling (Toluene, RT, 2.5 mL/min, Buckyprep-D semipreparative column). (f) MALDI Mass-spectra of the collected fraction after step 4 and step 5; the signal of the YGd@C_{80} fragment is marked with *.

HPLC separation of {Y_2-D_{5h}}

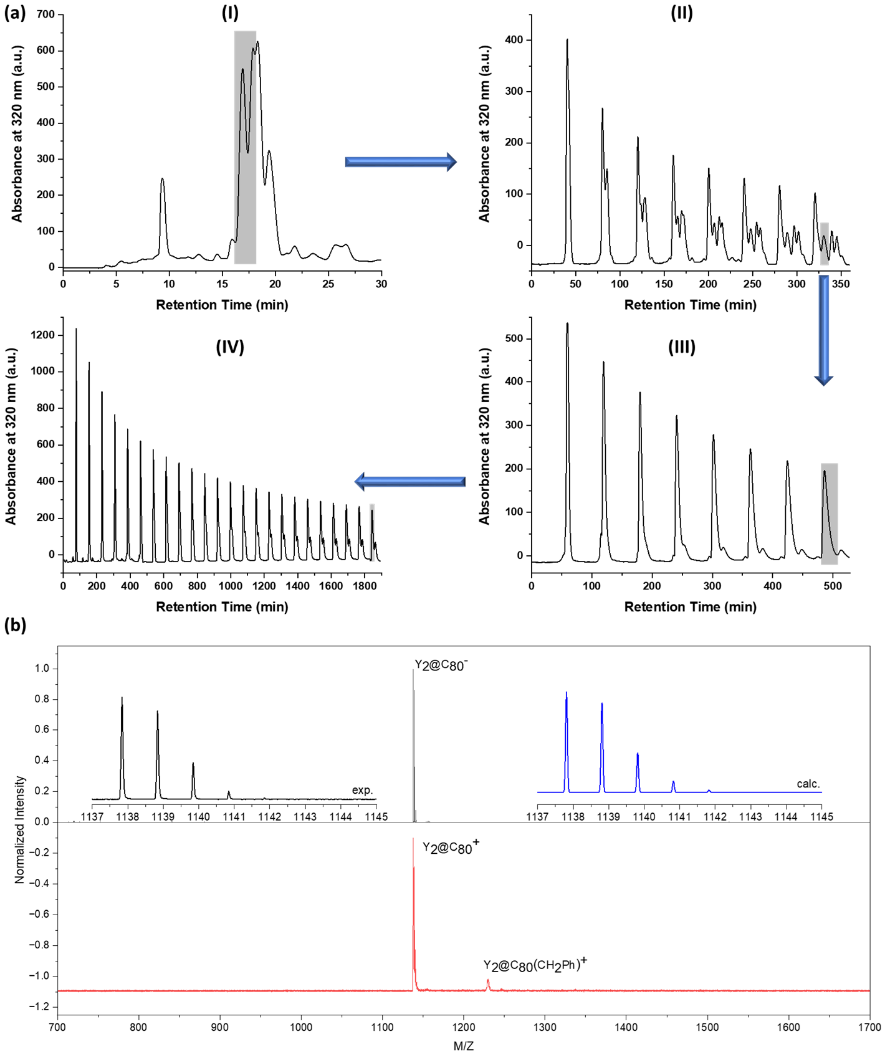

Figure S2. {Y_2-D_{5h}} was obtained through four steps HPLC separation shown in (a). (I) HPLC profile of the mixture of benzyl-derivatized Y-EMFs; the highlighted fraction contained {Y_2-D_{5h}} and was collected for further separation (linear combination of two 4.6 × 250 mm Buckyprep columns; flow rate 1.6 mL/min; injection volume 800 μL; toluene as eluent; 40 °C). (II) Recycling HPLC separation of the fraction collected in the first step; the highlighted fraction was collected for further separation (10 × 250 mm Buckyprep column; flow rate 2.0 mL/min; injection volume 4.5 mL; toluene as eluent). (III) HPLC separation of the fraction collected in the second step; the highlighted fraction was collected for further separation (10 × 250 mm Buckyprep-M column; flow rate 1.0 mL/min; injection volume 4.5 mL; toluene as eluent). (IV) HPLC separation of the fraction collected in the third step; pure {Y_2-D_{5h}} was obtained as the highlighted fraction (10 × 250 mm Buckyprep column; flow rate 1.0 mL/min; injection volume 4.5 mL; toluene as eluent). (b) Matrix-assisted laser desorption/ionization time-of-flight (MALDI TOF) mass-spectra of {Y_2-D_{5h}}. Negative (top) and linear positive (bottom) ionization modes, 1,1,4,4-tetraphenyl-1,3-butadiene was used as matrix. Resolution in positive mode is not high enough for analysis of isotopic distribution. In the negative ion mode, strong fragmentation does not allow for detection of molecular peak, but spectral resolution is sufficient to prove correct isotopic distribution of the Y_2@C_{80} fragment.

Variable-temperature CW X-band EPR spectra

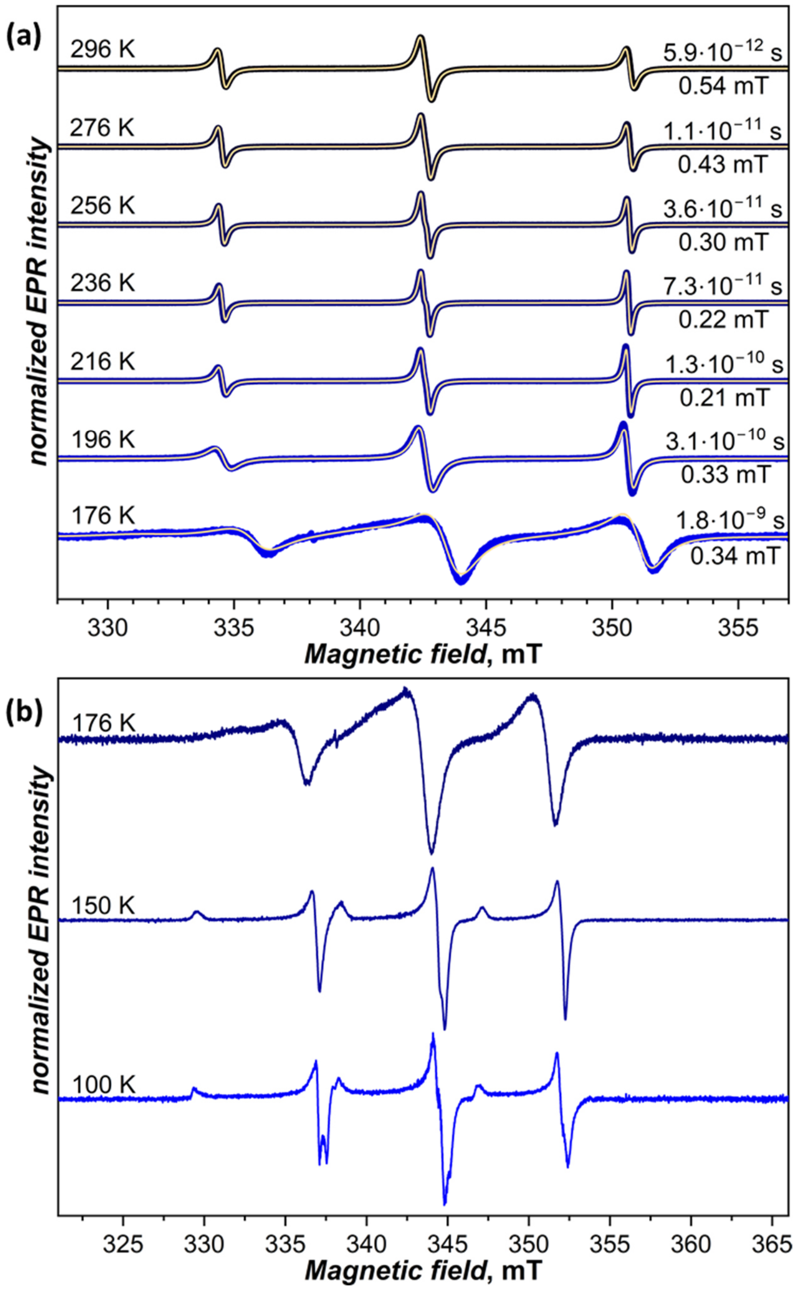

Figure S3. CW X-band EPR spectra of {Y_2-I_h} in toluene measured at different temperatures: (a) liquid solution (thick blue curves), and their fitting for slow motion regime using *chili* function in EasySpin (thin yellow curves); (b) frozen solution.

Fitting of the spectra in slow-motion regime employed *g* and *A*-tensors determined for 150 K. The only fitting parameters were rotation correlation time (T_{corr}) and Lorentzian linewidth. Fitted values are listed for each temperature. Note that the spectra 276 K and 296 K show essentially isotropic regime, which results in not very reliable values of T_{corr}. At the same time, these temperatures also demonstrate anomalous increase of the Lorentzian linewidth.

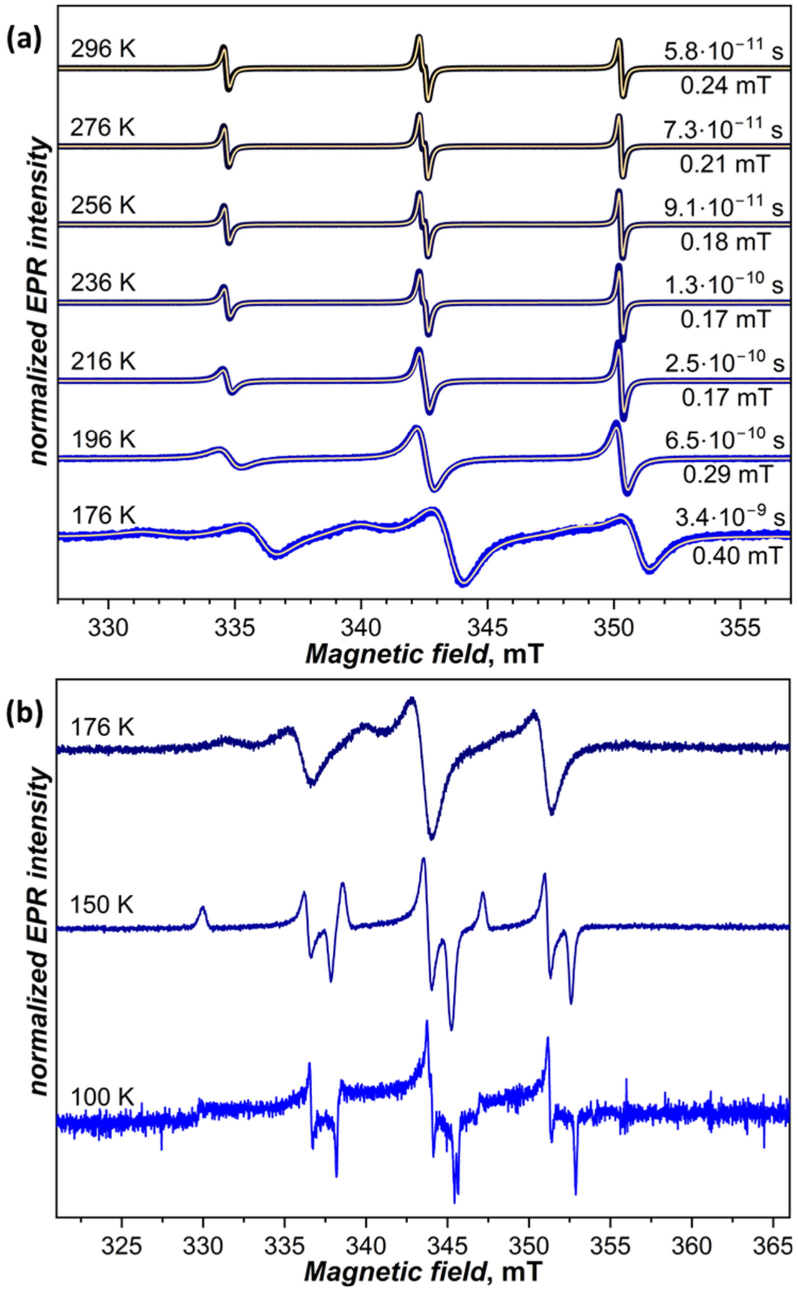

Figure S4. CW X-band EPR spectra of {Y_2-D_{5h}} in toluene measured at different temperatures: (a) liquid solution (thick blue curves), and their fitting for slow motion regime using *chili* function in EasySpin (thin yellow curves); (b) frozen solution.

Fitting of the spectra in slow-motion regime employed *g* and *A*-tensors determined for 150 K. The only fitting parameters were rotation correlation time (T_{corr}) and Lorentzian linewidth. Fitted values are listed for each temperature. Note that τ_r times of {Y_2-D_{5h}} are systematically longer that those of {Y_2-I_h}.

Table S1. EPR parameters of selected Y-centered radicals

	g	A, MHz	conditions	Ref.
**Y–Y bond**				
**Y_2@I_h-C_{80}(CH_2Ph)**	g_{iso} = 1.973	a_{iso} = 224	tol, RT, X-band	2
	$g_{x,y}$ = 1.962<br>g_z = 1.998	$a_{x,y}$= 208, a_z = 246	tol, 150 K, X-band	2
	g_x = 1.958<br>g_y = 1.962<br>g_z = 1.998	$a_{x,y}$ = 199, a_z = 258	tol, 20 K, ESE-W-band	tw
**Y_2@D_{5h}-C_{80}(CH_2Ph)**			tol, RT, X-band	tw
			tol, 150 K, X-band	tw
			tol, 20 K, ESE-W-band	tw
**Y_2@I_h-C_{80}(CF_3)**	g_{iso} = 1.9728	a_{iso} = 225	tol, RT, X-band	15
	$g_{x,y}$ = 1.9610<br>g_z = 1.9985	Y1: $a_{x,y}$= 215, a_z = 249<br>Y2: $a_{x,y}$= 207, a_z = 242	tol, 150 K, X-band	15
**Y_2@I_h-C_{80}(CH_2Ph)-pyr$_2$**	g_{iso} = 1.973	a_{iso} = 222, 230	tol, RT, X-band	16
	g_x = 1.9591<br>g_y = 1.9616<br>g_z = 1.9997	Y1: $a_{x,y}$= 219, a_z = 246<br>Y2: $a_{x,y}$= 207, a_z = 258	tol, 150 K, X-band	16
**Y_2@I_h-C_{79}N**	g_{iso} = 1.9740	a_{iso} = 215	tol, RT, X-band	17
	$g_{x,y}$ = 1.961<br>g_z = 1.999	$a_{x,y}$= 209, a_z = 253	@MOF, 80 K, X-band	18
**ThY@D_{3h}-C_{78}**	g_{iso} = 1.825	a_{iso} = 200	CS_2, 150–220 K, X-band	19
**(Cp^{iPr5})$_2$$Y_2$$I_3$**	g_x = 1.989<br>g_y = 1.995<br>g_z = 2.012	$a_{x,y}$ = 5, a_z = 2	3-methylpentane, 10 K, X-band	20
**[K(crypt)][(Cp^{An})$_2$Y(μ-H)]$_2$**	g_{iso} = 1.99	a_{iso} = 111	THF, RT, X-band	21
localized Y(II)/Y(III)	$g_{x,y}$ = 1.99<br>g_z = 2.00	Y(II): $a_{x,y}$= 35, a_z = 21	THF, 77 K, X-band	21
**molecular Y(II)**				
**Y(NHAr*)$_2$**	g_{iso} = 1.995	a_{iso} = 49	Et_2O, RT, X-band	22
	$g_{x,y}$ = 2.004<br>g_z = 1.985	$a_{x,y}$= 41, a_z = 39	Et_2O, 60 K, X-band	22
**[Y($^tBu_3C_6H_3$)$_2$]**	$g_{x,y}$ = 2.005<br>g_z = 2.085	a_z = 88	metylcyclohexane, 77 K X-band	23
**[($C_5H_4SiMe_3$)$_3$Y]$^-$**	g_{iso} = 1.991	a_{iso} = 102	Et_2O, RT, X-band	24
	$g_{x,y}$ = 1.986<br>g_z = 2.001	$a_{x,y}$= 101, a_z = 99	THF, 50 K, X-band	25
	$g_{x,y}$ = 1.986<br>g_z = 1.999	$a_{x,y}$= 101, a_z = 100	dil SC, 200 K, X-band	25
**[[{$C_5H_3(SiMe_3)_2$}$_2$YCp]$^-$**	g_{iso} = 1.9904	a_{iso} = 96	THF, RT, X-band	26
**[[{$C_5H_3(SiMe_3)_2$}$_3$Y]$^-$**	g_{iso} = 1.9908	a_{iso} = 101	THF, RT, X-band	26
**[{$C_5H_3(SiMe_3)_2$}$_2$YCp^{Me}]$^-$**	g_{iso} = 1.9904	a_{iso} = 101	THF, RT, X-band	26

	g	A, MHz	conditions	Ref.
**[Cp$_3$Y(THF)]$^-$**	g_{iso} = 1.9905	a_{iso} = 119	THF, RT, X-band	26
**[Cp$^{Me}{}_3$Y(THF)]$^-$**	g_{iso} = 1.9903	a_{iso} = 131	THF, RT, X-band	26
**[Cp$^{t}{}_3$Y]$^-$**	g_{iso} = 1.988	a_{iso} = 142	Et$_2$O, RT, X-band	27
	$g_{x,y}$ = 1.984<br>g_z = 2.002	$a_{x,y}$= 142, a_z = 133	Et$_2$O, 77 K, X-band	27
**[(C$_5$Me$_4$H)$_3$Y]$^-$**	g_{iso} = 1.986	a_{iso} = 180	THF, RT, X-band	28
	$g_{x,y}$ = 1.981<br>g_z = 2.001	$a_{x,y}$= a_z = 175	THF, 77 K, X-band	28
**[(C$_5$Me$_4$H)$_2${N(SiMe$_3$)$_2$}Y]$^-$**	g_{iso} = 1.975	a_{iso} = 197	RT, X-band	29
	g_x = 1.998<br>g_y = 1.985<br>g_z = 1.941	a_x = 185<br>a_y = 186<br>a_z = 178	77 K, X-band	29
**[(C$_5$Me$_5$)$_2${N(SiMe$_3$)$_2$}Y]$^-$**	g_{iso} = 1.975	a_{iso} = 206	RT, X-band	29
	g_x = 1.999<br>g_y = 1.985<br>g_z = 1.942	a_x = 208<br>a_y = 217<br>a_z = 210	77 K, X-band	29
**[{N(SiMe$_3$)$_2$}$_3$Y]$^-$**	g_{iso} = 1.976	a_{iso} = 304	THF, −78C, X-band	30
**[Y(OAr$^{t\text{-}Bu,t\text{-}Bu,Me}$)$_3$]$^-$**	$g_{x,y}$ = 1.97<br>g_z = 2.00	$a_{x,y}$= 428, a_z = 416	THF, 77 K, X-band	31
**[Y(OAr$^{Ad,Ad,t\text{-}Bu}$)$_3$]$^-$**	g_{iso} = 1.98	a_{iso} = 425	THF, RT, X-band	31
	$g_{x,y}$ = 1.97<br>g_z = 2.00	$a_{x,y}$= 432, a_z = 414	THF, 77 K, X-band	31
**Y(Cp^{iPr5})$_2$**	g_{iso} = 1.998	$a_{x,y}$= 500, a_z = 515	3-methylpentane, 5 K, X-band	32
**YO**	$g_{x,y}$ = 2.0021<br>g_z = 2.0016	$a_{x,y}$= −798 , a_z = −827	Ne matrix, 4 K, X-band	33
	$g_{x,y}$ = 2.000<br>g_z = 2.000	$a_{x,y}$= −791 , a_z = −822	Ar matrix, 4 K, X-band	33

Table S2. Spin parameters of {Gd_2-I_h}, {YGd-I_h}, Gd^{III} and Eu^{II} metallofullerenes and selected Gd-compounds.

	S	g	D (strain), GHz	E (strain), GHz	conditions	Ref.
**{Gd_2-I_h}**	15/2	g_{iso} = 1.987	1.03 (0.029)	0.23 (0.027)	tol., W-band, 6 K<br>tol., X, Q-band, 100 K	t.w.<br>[1]
**Gd_2@C_{79}N**	15/2	g_{iso} = 1.978	0.97(0.11)	0.11	CS_2, X,W-band, 4–20 K	[34, 35]
**Gd_2@C_{79}N**	15/2	g_{iso} = 1.99	0.96 (0.060)	0.14 (0.045)	tol., X, Q-band, 3–8 K	[36]
**[Gd_2@I_h-C_{80}]$^-$**	15/2	g_{iso} = 1.99	1.02	0.31	Q-band, 6 K	[37]
**{YGd-I_h}**	4	$g_\perp$ = 1.977,<br>$g_\parallel$ = 1.988	3.75 (0.18)	0.41 (0.07)	tol., W-band, 6 K	t.w.
**Gd@C_{82}**	7/2	1.99/1.99/2.00	6.30	0.54	pwdr, X,W-band, 4–20 K	[38]
**Gd@C_{82}**	7/2(3)	2.009/2.010/1.9775	7.72	0.21	TCB, X,W-band, 4–20 K	[38]
**Gd@C_{82}(Mrph)$_5$**	7/2	g_{iso} = 1.96	9.20	0.84	tol, X-band, 20 K	[39]
**Gd@C_{82}(Mrph)$_7$**	7/2	g_{iso} = 1.98	6.52	0.65	tol, X-band, 20 K	[39]
**Gd@C_{82}(Mrph)$_9$**	7/2	g_{iso} = 1.99	7.62	0.76	tol, X-band, 20 K	[39]
**Gd_3N@C_{80}**	21/2	g_{iso} = 1.995	11.5 per Gd^{3+}	n/a	powder, 5 K, 210 GHz, 315 GHz	[40]
**Eu@C_{74}**	7/2	g_{iso} = 1.99	3.90 (0.18)	0.39 (0.09)	CS_2, X-band, 4 K	[41]
**Eu@C_{80}**	7/2	g_{iso} = 1.99	8.24 (0.18)	0.08 (0.09)	CS_2, X-band, 4 K	[41]
**Eu@C_{82}-C_s**	7/2	g_{iso} = 1.99	8.69 (0.18)	0.08 (0.09)	CS_2, X-band, 4 K	[41]
**Eu@C_{84}**	7/2	g_{iso} = 1.99	8.39 (0.18)	0.54 (0.09)	CS_2, X-band, 4 K	[41]
**Eu@C_{74}**	7/2	1.994/1.989/1.988	3.83	0.13	CS_2, X, W-band, 1.5–20 K	[42]
**Eu@C_{82}-C_s**	7/2	1.995/1.993/1.995	8.75	0.22	CS_2, X, W-band, 1.5–20 K	[42]
**Eu@C_{82}-C_2**	7/2	1.992/1.993/1.993	8.74	1.81	CS_2, X, W-band, 1.5–20 K	[42]
**Eu@C_{82}-C_{2v}**	7/2	1.993/1.992/1.994	8.17	0.12	CS_2, X, W-band, 1.5–20 K	[42]
**Eu@C_{72}**	9/2	1.97/1.98/2.0	3.96	0.68	CS_2, W-band, 9 K	[43]

Spin Hamiltonian includes higher-order ZFS terms

T_1 and T_m electron spin relaxation at W-band

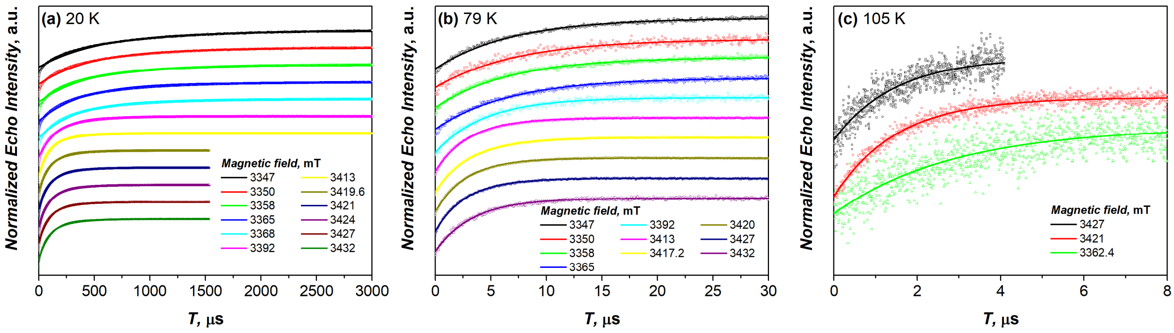

Figure S5. W-band EPR inversion recovery data for {Y_2-I_h} in d_8-toluene, recorded at different magnetic fields (points) at (a) 20 K, (b) 79 K and (c) 105 K. The corresponding 1−exp(−T/T_1) fits, where T represents the recovery delay after the inversion pulse and T_1 the spin-lattice relaxation time, are shown as solid lines.

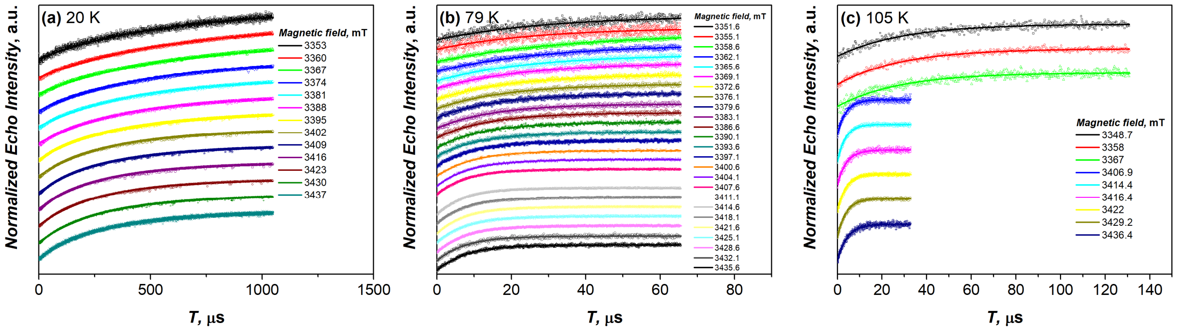

Figure S6. W-band EPR inversion recovery data for {Y_2-D_{5h}} in d_8-toluene, recorded at different magnetic fields (points) at (a) 20 K, (b) 79 K and (c) 105 K. The corresponding 1−exp(−T/T_1) fits, where T represents the recovery delay after the inversion pulse and T_1 the spin-lattice relaxation time, are shown as solid lines.

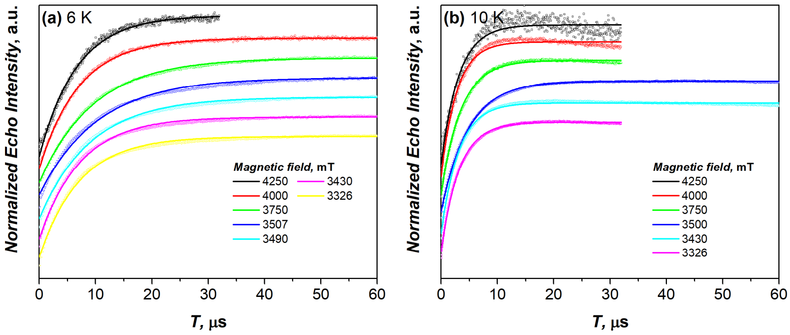

Figure S7. W-band EPR inversion recovery data for {YGd-I_h} in d_8-toluene, recorded at different magnetic fields (points) at (a) 6 K and (b) 10 K. The corresponding 1−exp(−T/T_1) fits, where T represents the recovery delay after the inversion pulse and T_1 the spin-lattice relaxation time, are shown as solid lines.

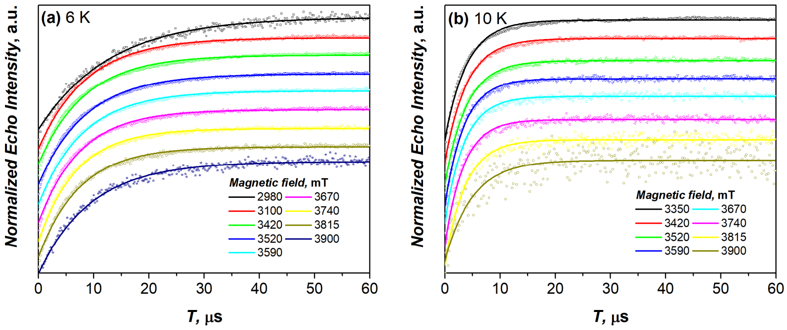

Figure S8. W-band EPR inversion recovery data for {Gd_2-I_h} in d_8-toluene, recorded at different magnetic fields (points) at (a) 6 K and (b) 10 K. The corresponding $1-\exp(-T/T_1)$ fits, where T represents the recovery delay after the inversion pulse and T_1 the spin-lattice relaxation time, are shown as solid lines.

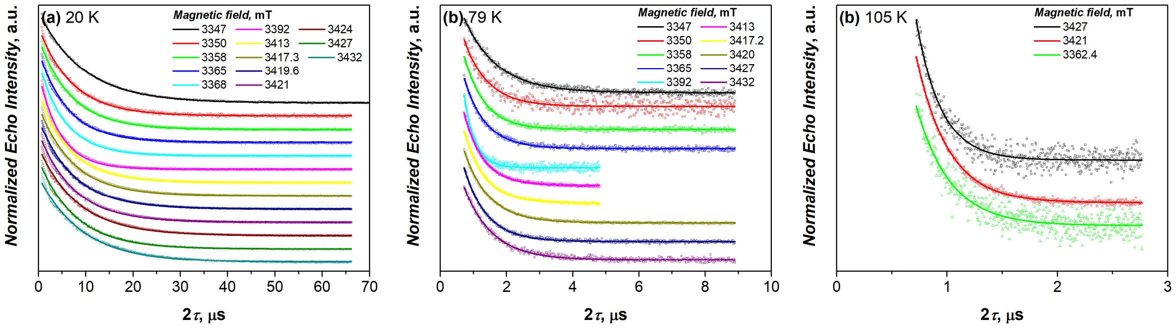

Figure S9. W-band EPR Hahn echo decays for {Y_2-I_h} in d_8-toluene, recorded at different magnetic fields (points) at (a) 20 K, (b) 79 K and (c) 105 K. The corresponding $\exp(-2\tau/T_m)$ fits, where τ represents the echo delay and T_m the phase-memory time, are shown as solid lines.

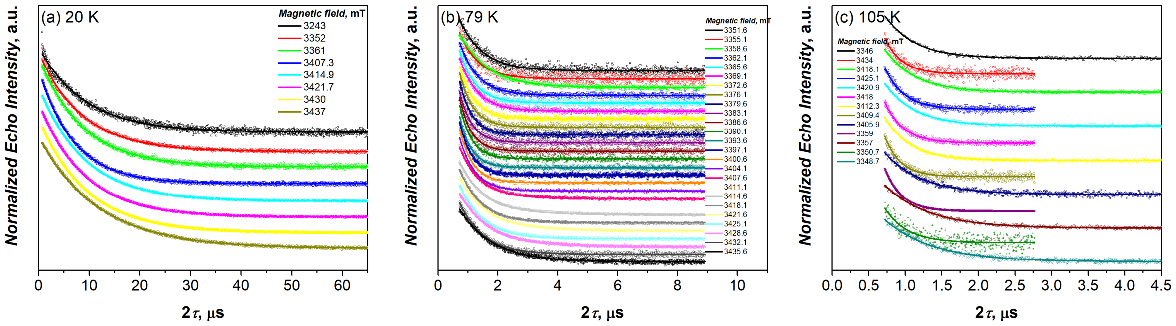

Figure S10. W-band EPR Hahn echo decays for {Y_2-D_{5h}} in d_8-toluene, recorded at different magnetic fields (points) at (a) 20 K, (b) 79 K and (c) 105 K. The corresponding $\exp(-2\tau/T_m)$ fits, where τ represents the echo delay and T_m the phase-memory time, are shown as solid lines..

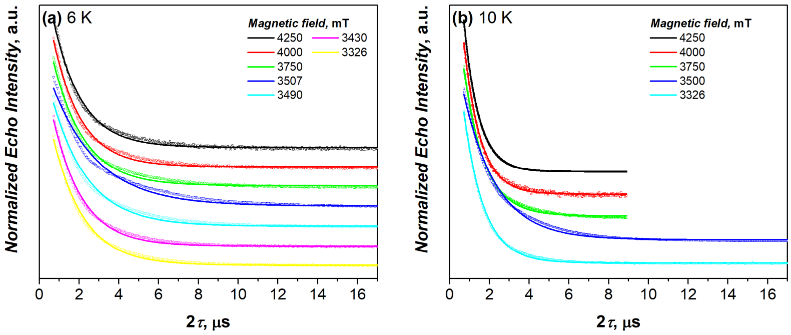

Figure S11. W-band EPR Hahn echo decays for {YGd-I_h} in d_8-toluene, recorded at different magnetic fields (points) at (a) 6 K and (b) 10 K. The corresponding exp(−2τ/T_m) fits, where τ represents the echo delay and T_m the phase-memory time, are shown as solid lines.

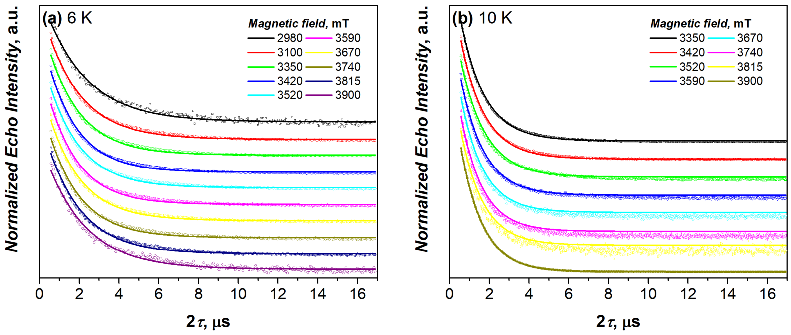

Figure S12. W-band EPR Hahn echo decays for {Gd_2-I_h} in d_8-toluene, recorded at different magnetic fields (points) at (a) 6 K and (b) 10 K. The corresponding exp(−2τ/T_m) fits, where τ represents the echo delay and T_m the phase-memory time, are shown as solid lines.

Raman spectra of {Y_2-I_h} and {Y_2-D_{5h}}

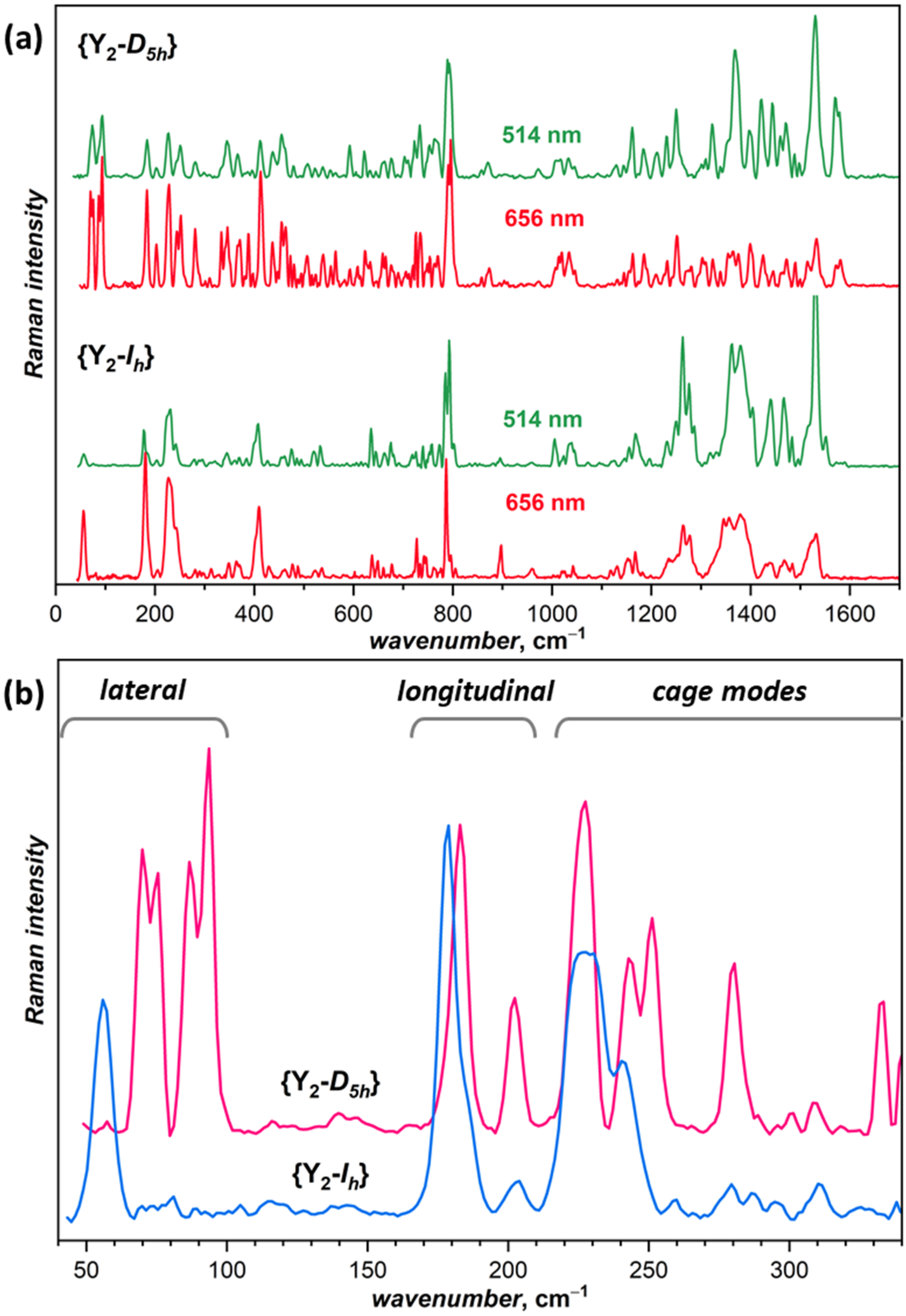

Figure S13. (a) Raman spectra of {Y_2-I_h} and {Y_2-D_{5h}} measured at 78 K with 514 and 656 nm excitation. (b) Comparison of the low-frequency part of the spectra of {Y_2-I_h} and {Y_2-D_{5h}}, 656 nm excitation.

Raman spectra of {Y_2-I_h}, {YTb-I_h}, and {Gd_2-I_h}

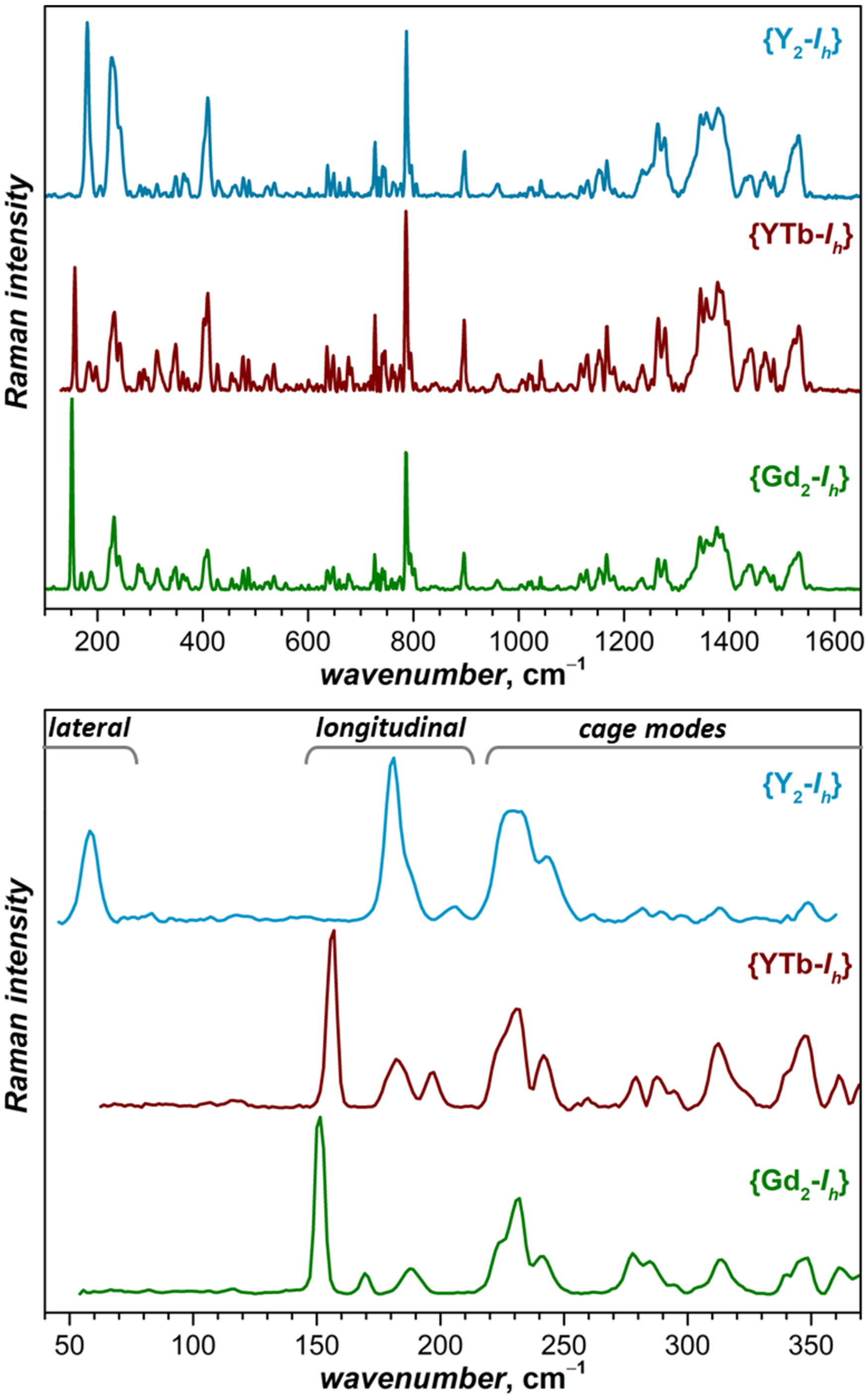

Figure S14. (top) Raman spectra of {Y_2-I_h}, {YTb-I_h}, and {Gd_2-I_h} measured at 78 K with 656 nm excitation (from Ref. [1]). (bottom) Comparison of the low-frequency part of the spectra of {Y_2-I_h}, {YTb-I_h}, and {Gd_2-I_h}. Raman spectra of {YGd-I_h} were not measured in this work, but close atomic mass of Gd and Tb suggests that the spectra of {YGd-I_h} and {YTb-I_h} should be very similar.

Metal-based vibrational modes

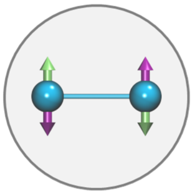

lateral anti-phase: libration

{Y_2-D_{5h}}: calc. 67, 82 cm^{-1}; exp. 71, 75 cm^{-1}
{Y_2-I_h}: calc. 50, 55 cm^{-1}; exp. 56 (?) cm^{-1}
{YGd-I_h}: calc. 43, 48 cm^{-1}
{Gd_2-I_h}: calc. 39, 43 cm^{-1}

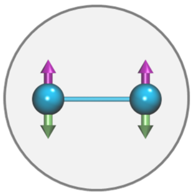

lateral in-phase: translation

{Y_2-D_{5h}}: calc. 92, 94 cm^{-1}; exp. 88, 94 cm^{-1}
{Y_2-I_h}: calc. 65, 69 cm^{-1}; exp. 56 cm^{-1}
{YGd-I_h}: calc. 54, 68 cm^{-1}
{Gd_2-I_h}: calc. 52, 55 cm^{-1}

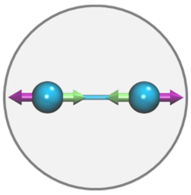

symmetric longitudinal: "M−M stretching"

{Y_2-D_{5h}}: calc. 181 cm^{-1}, exp. 184 cm^{-1}
{Y_2-I_h}: calc. 175 cm^{-1}, exp. 181 cm^{-1}
{YGd-I_h}: calc. 155 cm^{-1}
{Gd_2-I_h}: calc. 146 cm^{-1}, exp. 151 cm^{-1}

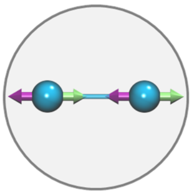

antisymmetric longitudinal: "translation"

{Y_2-D_{5h}}: calc. 199 cm^{-1}, exp. 203 cm^{-1}
{Y_2-I_h}: calc. 195 cm^{-1}, exp. 205 cm^{-1}
{YGd-I_h}: calc. 192 cm^{-1}
{Gd_2-I_h}: calc. 165 cm^{-1}, exp. 170 cm^{-1}

Figure S15a. Different types of metal-based modes in dimetallofullerenes and their calculated and experimental frequencies.

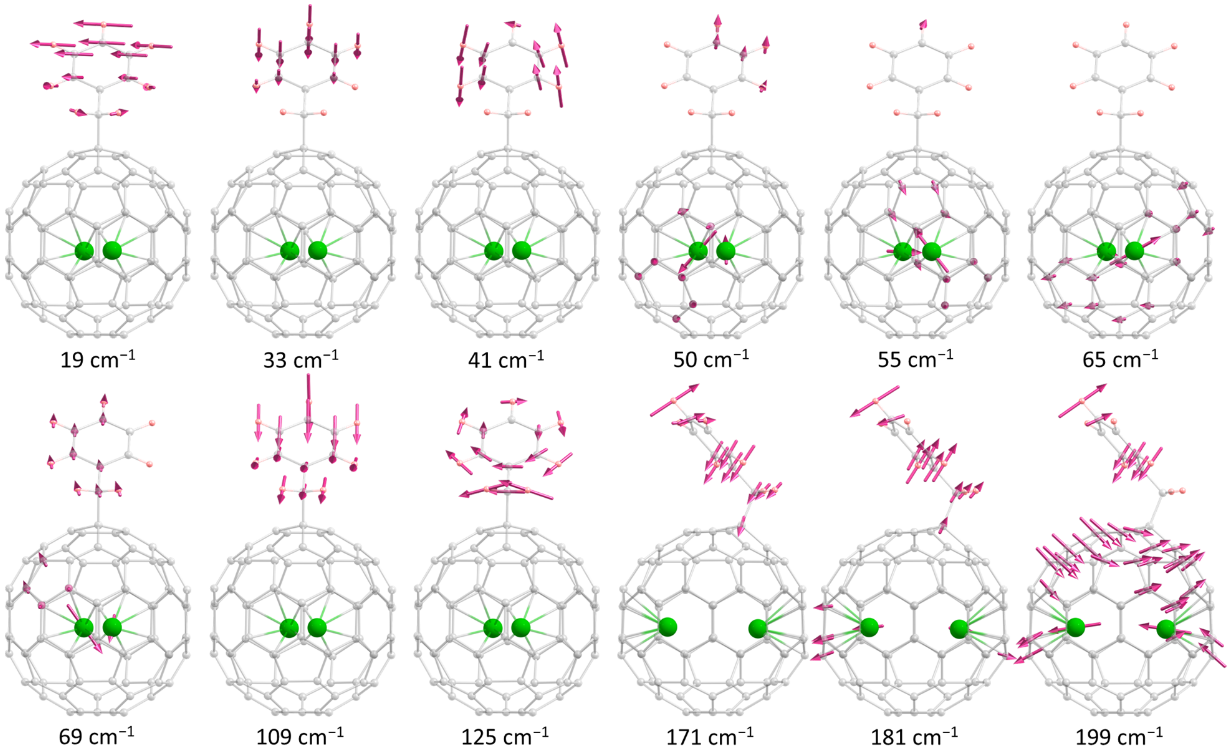

Figure S15b. Vibrational displacements of the lowest-frequency modes of {Y_2-I_h}.

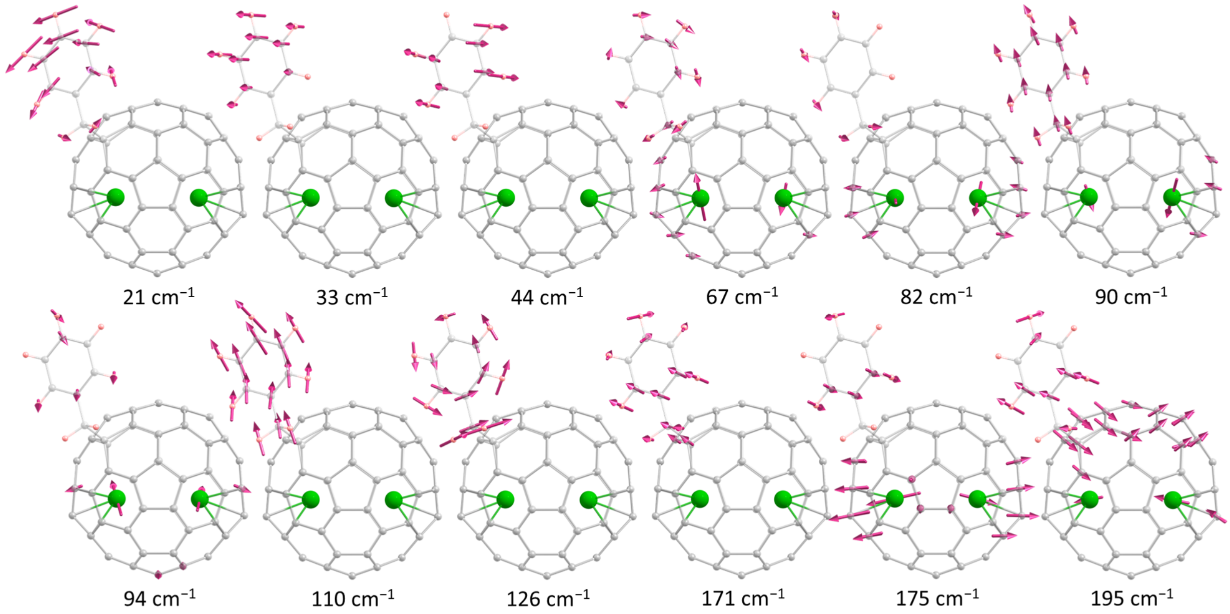

Figure S15c. Vibrational displacements of the lowest-frequency modes of {Y_2-D_{5h}}.

DFT-optimized Cartesian coordinates of {Y_2-I_h}

C	2.798950000	-2.962046000	1.244517000	C	2.893735000	1.531718000	-2.656467000
C	2.810697000	-2.078405000	2.395293000	C	2.989830000	3.028730000	0.705297000
C	1.572692000	-2.266606000	3.122690000	C	2.984495000	3.017989000	-0.725599000
C	1.560464000	-3.668460000	1.249070000	C	1.114814000	3.272348000	2.323144000
C	0.805106000	-3.248683000	2.403714000	C	1.783399000	3.686986000	1.143249000
C	0.885000000	-4.015048000	0.026768000	C	-1.090551000	2.375885000	3.094515000
C	1.550432000	-3.705684000	-1.194344000	C	-0.307758000	3.346964000	2.356307000
C	3.427161000	-2.568525000	0.018057000	C	-2.979970000	0.925313000	2.350285000
C	2.800182000	-2.986416000	-1.199226000	C	-2.331303000	2.194397000	2.364887000
C	4.144784000	-1.342346000	0.007747000	C	-3.719015000	-0.831682000	0.733205000
C	3.436219000	-0.828180000	2.370220000	C	-3.719475000	-0.842593000	-0.701102000
C	4.423039000	-0.503393000	1.259900000	C	-3.672406000	0.545831000	1.169988000
C	2.867749000	0.220376000	3.126280000	C	-3.668519000	0.524643000	-1.154597000
C	0.976897000	-1.228714000	3.905687000	C	-3.646665000	1.384615000	-0.000087000
C	1.656968000	0.037427000	3.900620000	C	-2.381920000	-0.160464000	-3.136323000
C	-0.477965000	-1.196670000	3.997497000	C	-1.211224000	0.019654000	-3.987807000
C	-0.611283000	-3.171570000	2.374385000	C	-2.974061000	0.888193000	-2.343712000
C	-1.217687000	-2.119507000	3.159967000	C	0.941881000	1.245871000	-3.925405000
C	-0.539489000	-4.014823000	0.033795000	C	1.711744000	2.184577000	-3.128306000
C	-1.283497000	-3.597464000	1.195707000	C	-0.507783000	1.302335000	-3.939822000
C	-1.288698000	-3.621488000	-1.131009000	C	1.780266000	3.675838000	-1.182306000
C	0.801926000	-3.307504000	-2.352825000	C	1.035201000	4.083022000	-0.023214000
C	-0.616923000	-3.217564000	-2.316605000	C	1.110466000	3.234400000	-2.354095000
C	2.818606000	-2.147114000	-2.356166000	C	-1.054031000	3.745951000	1.201873000
C	1.578091000	-2.335300000	-3.085433000	C	-2.307921000	3.034361000	1.207199000
C	4.122637000	-0.506091000	-1.116580000	C	-2.931100000	2.616713000	-0.011220000
C	3.445417000	-0.877611000	-2.323491000	C	-0.390735000	4.072942000	-0.021828000
C	4.221631000	0.918226000	0.724486000	C	-2.333355000	2.157124000	-2.381975000
C	4.166491000	0.886504000	-0.676146000	C	-1.091516000	2.327983000	-3.116671000
C	2.916667000	1.591439000	2.676952000	C	-0.311885000	3.311315000	-2.390325000
C	3.562672000	1.948789000	1.443073000	C	-2.310614000	3.015841000	-1.236990000
C	0.938314000	1.302519000	3.905769000	C	-1.056392000	3.725963000	-1.241521000
C	1.725879000	2.239107000	3.115154000	Y	-0.066242000	0.010192000	1.977075000
C	-1.219384000	0.081500000	4.016872000	Y	0.012737000	0.029433000	-1.974344000
C	-0.506709000	1.360491000	3.928740000	C	5.884553000	-0.735030000	1.836821000
C	-2.401052000	-1.464970000	2.683702000	H	5.992221000	-0.064045000	2.702010000
C	-2.390343000	-0.111731000	3.165741000	H	5.916624000	-1.767995000	2.214076000
C	-2.489223000	-2.952432000	0.747416000	C	7.001448000	-0.504336000	0.852246000
C	-2.493922000	-2.966063000	-0.688635000	C	7.579332000	0.764810000	0.704019000
C	-3.049588000	-1.851586000	1.460332000	H	7.213113000	1.596104000	1.311364000
C	-3.051369000	-1.876968000	-1.414300000	C	8.617991000	0.976017000	-0.204629000
C	-1.217014000	-2.170247000	-3.105614000	H	9.058428000	1.969748000	-0.302951000
C	-2.398371000	-1.507863000	-2.640253000	C	9.094830000	-0.082128000	-0.981548000
C	0.983934000	-1.298482000	-3.872436000	H	9.908329000	0.081115000	-1.690436000
C	-0.469708000	-1.257977000	-3.949763000	C	8.529655000	-1.351832000	-0.841810000
C	2.861211000	0.167334000	-3.104205000	C	7.491106000	-1.558674000	0.067523000
C	1.668496000	-0.026393000	-3.896966000	H	7.055511000	-2.555218000	0.174573000
C	3.530103000	1.917654000	-1.434634000	H	8.900630000	-2.185743000	-1.440295000

DFT-optimized Cartesian coordinates of {Y_2-I_h}

C	-3.164959000	1.417206000	-1.226547000
C	-3.182754000	-1.424678000	-1.179673000
C	-3.588441000	0.714878000	-0.049793000
C	-2.712470000	0.706157000	-2.377292000
C	-3.598853000	-0.678551000	-0.025453000
C	-2.717884000	-0.757799000	-2.348996000
C	-2.562434000	2.662233000	-0.787390000
C	-2.581511000	-2.655377000	-0.695840000
C	-3.186914000	1.457952000	1.114321000
C	-1.719540000	1.365979000	-3.212051000
C	-3.206312000	-1.383060000	1.163813000
C	-1.723976000	-1.450272000	-3.157036000
C	-2.576724000	2.686074000	0.646677000
C	-2.597262000	-2.624855000	0.734108000
C	-0.768897000	0.664297000	-4.062845000
C	-0.771096000	-0.781381000	-4.029698000
C	-1.075166000	2.561166000	-2.714767000
C	-1.088776000	-2.633640000	-2.624860000
C	-1.471565000	3.222189000	-1.502286000
C	-1.489899000	-3.248366000	-1.388609000
C	-2.760013000	0.788070000	2.297004000
C	-2.764837000	-0.676938000	2.322054000
C	0.508072000	1.361582000	-3.949496000
C	0.501382000	-1.481750000	-3.891258000
C	0.285775000	2.556775000	-3.156871000
C	0.270936000	-2.651641000	-3.066161000
C	-1.498764000	3.271255000	1.365343000
C	-1.523474000	-3.193922000	1.476027000
C	-0.463019000	3.929525000	-0.789193000
C	-0.487585000	-3.929307000	-0.647321000
C	-1.779010000	1.469976000	3.122465000
C	-1.790807000	-1.342518000	3.171563000
C	-0.475991000	3.953746000	0.645038000
C	-0.505391000	-3.898607000	0.779538000
C	-1.128951000	2.651194000	2.604632000
C	-1.147618000	-2.537964000	2.685642000
C	1.788274000	0.672049000	-3.825103000
C	1.784511000	-0.799866000	-3.799773000
C	1.313084000	3.201218000	-2.385866000
C	1.298326000	-3.271422000	-2.274505000
C	0.910275000	3.915342000	-1.221005000
C	0.896443000	-3.944944000	-1.073818000
C	-0.843885000	0.794411000	4.011122000
C	-0.843577000	-0.648879000	4.042023000
C	0.889064000	3.955111000	1.099779000
C	0.860786000	-3.884770000	1.240956000
C	0.227508000	2.658406000	3.069603000
C	0.215590000	-2.541723000	3.145638000
C	1.745553000	3.933528000	-0.053402000
C	1.728661000	-3.948482000	0.078266000
C	0.436886000	1.487819000	3.895531000
C	2.818016000	1.360177000	-3.114725000
C	0.423699000	-1.357057000	3.966693000
C	2.818969000	-1.472874000	-3.078250000
C	2.600222000	2.607724000	-2.410599000
C	2.579502000	-2.688605000	-2.318759000
C	1.265978000	3.276691000	2.293541000
C	1.229229000	-3.190902000	2.398859000
C	3.830559000	0.644072000	-2.384129000
C	3.832066000	-0.745832000	-2.374176000
C	3.436748000	2.618775000	-1.239706000
C	3.421160000	-2.674932000	-1.146737000
C	2.994914000	3.240662000	-0.034803000
C	2.988723000	-3.266363000	0.078837000
C	1.713768000	0.791620000	3.817618000
C	1.717437000	-0.681695000	3.855050000
C	2.547517000	2.673696000	2.353575000
C	2.678559000	-2.807237000	2.630252000
C	4.186434000	1.387557000	-1.207204000
C	4.173177000	-1.453751000	-1.162793000
C	3.410093000	2.647704000	1.195369000
C	3.414447000	-2.647615000	1.284434000
C	2.756567000	1.459417000	3.104387000
C	2.759067000	-1.375030000	3.182992000
C	4.474285000	0.715834000	0.016820000
C	4.458722000	-0.747525000	0.034780000
C	4.152917000	1.419603000	1.220277000
C	4.099774000	-1.423112000	1.251836000
C	3.778062000	0.720985000	2.417133000
C	3.728009000	-0.667723000	2.435494000
Y	-0.027629000	0.016748000	1.954191000
Y	-0.011585000	-0.000694000	-1.957128000
C	3.346123000	-3.883720000	3.575245000
H	3.262817000	-4.854085000	3.062707000
H	2.725570000	-3.940396000	4.482438000
C	4.779906000	-3.603719000	3.942576000
C	5.093638000	-2.862043000	5.091100000
H	4.286384000	-2.493758000	5.729118000
C	6.420997000	-2.595860000	5.432158000
H	6.644866000	-2.020172000	6.331978000
H	8.497108000	-2.864152000	4.892380000
C	7.458775000	-3.069002000	4.626342000
C	7.160729000	-3.812048000	3.481645000
C	5.832158000	-4.075253000	3.143920000
H	5.607415000	-4.660881000	2.248992000
H	7.965897000	-4.191920000	2.850268000

Derivatives of g and A-tensors with respect to normal modes

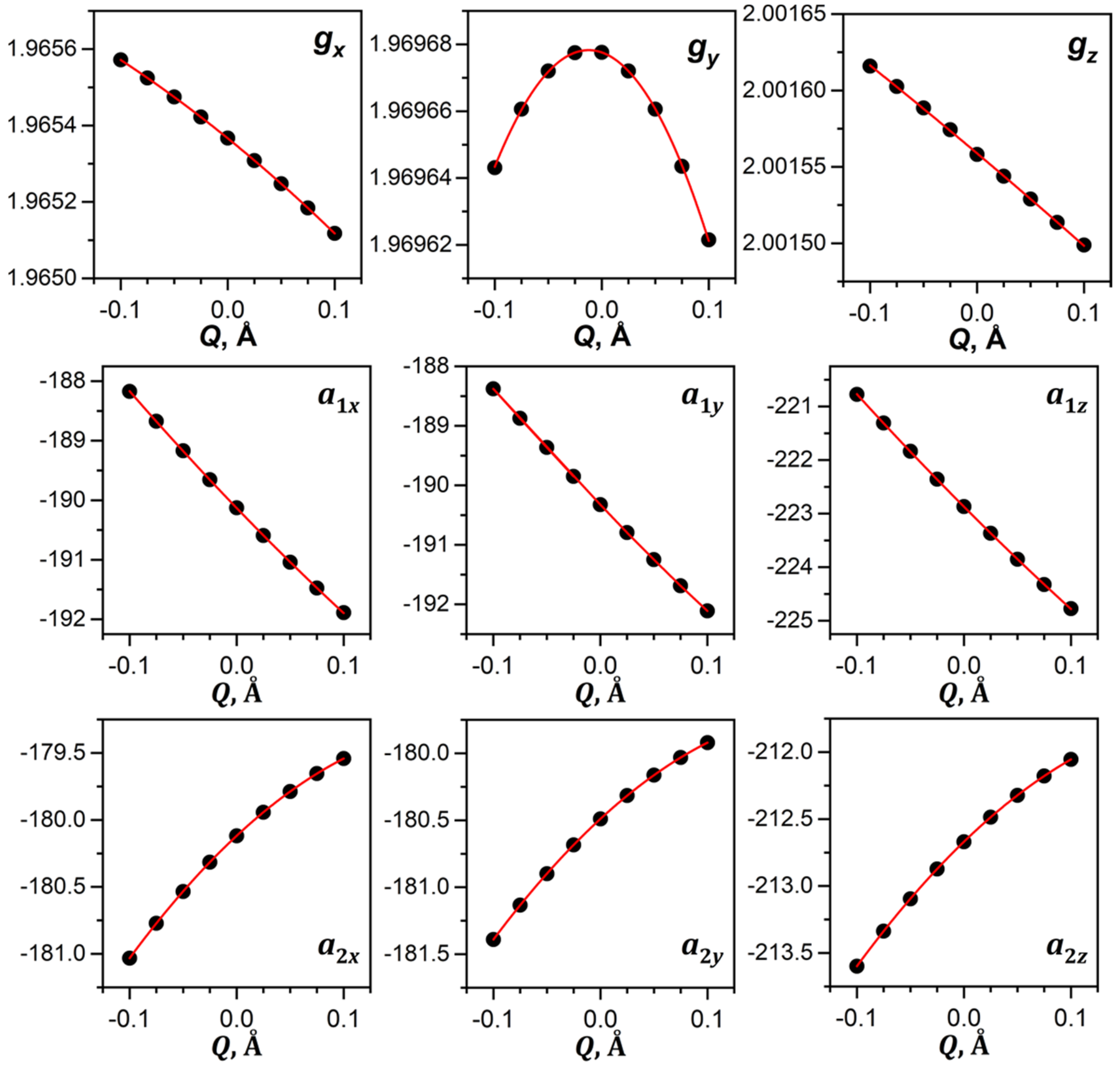

Figure S16. Representative example of the derivatization of g and A-tensors with respect to a normal mode Q (mode N 4, {Y_2-I_h} isomer). Dots are computed g_i and $a_{1,2i}$ values ($i = x, y, z$; indices 1 and 2 label Y atoms, hyperfine constants are in MHz) with different degree of geometry distortion along the normal mode coordinate, while red curves are fits by a quadratic polynomial. $\partial g_i/\partial Q$ and $\partial a_{1,2i}/\partial Q$ derivatives are determined as coefficients at the linear term in the fitted function.

Table S3a. Computed vibrational frequencies, contributions of Y_2, CH_2Ph, and C_{80} to a given mode, and absolute values of $\partial g_i/\partial Q$ derivatives for {Y_2-I_h}.

N	$\boldsymbol{\nu}$ cm^{-1}	Y_2	CH_2Ph	C_{80}	$\lvert\partial g_x/\partial Q\rvert$ Å^{-1}	$\lvert\partial g_y/\partial Q\rvert$ Å^{-1}	$\lvert\partial g_z/\partial Q\rvert$ Å^{-1}
1	18.6	0.00	**0.77**	**0.23**	2.49E-05	5.73E-05	1.43E-05
2	33.1	0.04	**0.77**	**0.19**	4.15E-05	1.07E-06	5.13E-06
3	40.8	0.00	**0.94**	0.06	4.60E-06	9.40E-06	3.40E-06
4	49.9	**0.88**	0.02	0.10	2.27E-03	1.11E-04	5.90E-04
5	54.9	**0.88**	0.01	0.11	1.88E-03	2.20E-03	2.00E-04
6	65.2	**0.85**	0.00	0.15	1.95E-03	9.42E-04	1.18E-03
7	68.9	**0.84**	0.02	0.13	2.98E-03	7.37E-03	1.37E-05
8	109.0	0.01	**0.79**	**0.20**	8.89E-05	3.30E-04	4.33E-06
9	125.1	0.00	**0.88**	0.12	2.40E-05	5.68E-05	3.67E-05
10	171.3	**0.19**	**0.46**	**0.35**	7.72E-04	5.58E-04	2.18E-04
11	180.6	**0.39**	**0.23**	**0.37**	1.60E-03	2.11E-03	5.63E-04
12	199.0	**0.46**	0.04	**0.49**	3.57E-04	2.99E-04	2.33E-05
13	215.9	0.00	0.00	**1.00**	1.14E-03	1.87E-03	1.15E-04
14	219.4	0.00	0.00	**1.00**	3.39E-04	5.34E-04	5.14E-05
15	225.7	**0.07**	0.09	**0.84**	7.88E-05	4.54E-04	9.19E-05
16	235.0	**0.08**	0.06	**0.86**	4.43E-04	3.29E-04	5.65E-05
17	272.8	0.00	0.00	**0.99**	4.16E-04	3.01E-04	2.54E-05
18	274.1	**0.05**	0.00	**0.94**	4.11E-04	2.46E-03	4.85E-04
19	280.5	0.00	0.01	**0.99**	1.67E-04	8.43E-04	9.33E-06
20	283.1	**0.05**	0.00	**0.95**	4.91E-04	8.28E-04	3.79E-04
21	289.2	**0.06**	**0.15**	**0.79**	1.29E-03	1.64E-03	4.34E-04
22	302.8	0.01	0.01	**0.99**	3.35E-04	6.04E-04	2.28E-04
23	315.0	**0.11**	0.00	**0.89**	1.35E-03	3.29E-03	4.00E-04

Table S3b. Computed vibrational frequencies, contributions of Y_2, CH_2Ph, and C_{80} to a given mode, and absolute values of $\partial g_i/\partial Q$ derivatives for {Y_2-D_{5h}}.

N	ν cm^{-1}	Y_2	CH_2Ph	C_{80}	$\lvert\partial g_x/\partial Q\rvert$ Å^{-1}	$\lvert\partial g_y/\partial Q\rvert$ Å^{-1}	$\lvert\partial g_z/\partial Q\rvert$ Å^{-1}
1	20.9	0.03	**0.75**	**0.22**	2.62E-05	1.13E-05	6.00E-06
2	33.1	0.01	**0.78**	**0.21**	5.41E-05	4.12E-05	2.22E-05
3	44.3	0.01	**0.96**	0.03	4.79E-05	4.21E-05	7.91E-05
4	67.4	**0.88**	0.02	0.10	6.88E-04	9.11E-04	1.84E-04
5	81.9	**0.90**	0.01	0.09	1.80E-03	7.67E-04	8.77E-04
6	90.2	**0.81**	0.05	0.13	3.91E-03	4.76E-03	2.47E-03
7	93.8	**0.81**	0.02	0.17	3.97E-03	2.09E-03	2.07E-03
8	109.8	0.04	**0.73**	**0.23**	7.59E-04	3.72E-04	4.43E-04
9	126.3	0.01	**0.87**	0.12	2.37E-05	9.58E-05	8.67E-07
10	171.4	**0.14**	**0.58**	**0.28**	3.20E-04	2.97E-04	6.20E-04
11	175.4	**0.55**	0.04	**0.41**	2.20E-03	3.42E-03	3.60E-03
12	195.2	**0.36**	**0.11**	**0.53**	7.67E-05	2.35E-04	3.28E-04
13	217.5	0.01	0.00	**0.99**	6.50E-05	2.87E-03	2.13E-04
14	219.0	0.02	0.02	**0.96**	1.68E-04	6.71E-04	1.59E-05
15	231.7	**0.06**	**0.11**	**0.83**	8.63E-05	2.28E-04	1.46E-04
16	243.0	**0.09**	0.05	**0.86**	1.29E-04	1.24E-04	8.15E-05
17	271.2	**0.13**	0.01	**0.86**	1.21E-03	2.72E-03	2.77E-03
18	276.5	0.01	0.00	**0.98**	2.08E-04	5.47E-04	8.41E-04
19	281.8	0.01	0.00	**0.99**	5.58E-04	1.77E-03	3.38E-04
20	287.8	0.01	**0.09**	**0.90**	1.89E-04	7.59E-04	1.37E-04
21	293.4	0.01	0.02	**0.97**	1.94E-04	7.05E-05	1.82E-04
22	300.2	0.01	0.03	**0.96**	6.36E-04	1.36E-03	3.34E-04
23	325.4	**0.10**	0.04	**0.87**	4.54E-04	7.44E-03	1.52E-03

Table S3c. Computed vibrational frequencies and absolute values of $\partial a_i/\partial \boldsymbol{Q}$ derivatives for {Y_2-I_h}.

N	ν cm^{-1}	$\lvert\partial a_{1x}/\partial Q\rvert$ MHz Å^{-1}	$\lvert\partial a_{1y}/\partial Q\rvert$ MHz Å^{-1}	$\lvert\partial a_{1z}/\partial Q\rvert$ MHz Å^{-1}	$\lvert\partial a_{2x}/\partial Q\rvert$ MHz Å^{-1}	$\lvert\partial a_{2y}/\partial Q\rvert$ MHz Å^{-1}	$\lvert\partial a_{2z}/\partial Q\rvert$ MHz Å^{-1}
1	18.6	2.94E-02	3.07E-02	4.25E-02	3.40E-01	3.24E-01	3.57E-01
2	33.1	7.70E-01	7.48E-01	7.69E-01	8.63E-01	8.43E-01	9.25E-01
3	40.8	7.95E-02	7.68E-02	8.71E-02	4.25E-02	3.64E-02	4.33E-02
4	49.9	1.86E+01	1.87E+01	2.01E+01	7.45E+00	7.35E+00	7.72E+00
5	54.9	1.08E+01	9.94E+00	9.55E+00	2.28E+01	2.19E+01	2.42E+01
6	65.2	6.18E+00	6.07E+00	5.99E+00	2.88E+00	3.12E+00	4.26E+00
7	68.9	3.75E+01	3.62E+01	4.03E+01	1.47E+01	1.45E+01	1.74E+01
8	109.0	3.96E+00	3.89E+00	4.08E+00	8.40E-01	8.37E-01	8.25E-01
9	125.1	5.51E-02	2.67E-02	5.63E-02	3.96E-01	3.81E-01	4.15E-01
10	171.3	2.60E+01	2.60E+01	2.95E+01	1.24E+01	1.25E+01	1.57E+01
11	180.6	3.10E+01	3.10E+01	3.68E+01	4.47E+01	4.47E+01	5.11E+01
12	199.0	7.05E+01	7.05E+01	7.21E+01	6.46E+01	6.47E+01	6.63E+01
13	215.9	2.61E+00	2.62E+00	2.90E+00	6.26E+00	6.31E+00	6.68E+00
14	219.4	3.12E+00	3.27E+00	3.33E+00	2.88E+00	3.02E+00	3.15E+00
15	225.7	3.01E+01	3.02E+01	3.16E+01	1.56E+01	1.56E+01	1.53E+01
16	235.0	1.88E+01	1.90E+01	1.90E+01	3.09E+01	3.10E+01	3.22E+01
17	272.8	4.98E+00	5.06E+00	4.92E+00	7.09E+00	7.11E+00	7.26E+00
18	274.1	2.97E+01	2.97E+01	3.17E+01	2.18E+01	2.18E+01	2.38E+01
19	280.5	3.91E+00	3.83E+00	4.34E+00	1.04E+00	9.23E-01	1.27E+00
20	283.1	2.85E+01	2.86E+01	3.03E+01	1.63E+01	1.63E+01	1.79E+01
21	289.2	2.31E+01	2.32E+01	2.48E+01	2.54E+01	2.54E+01	2.72E+01
22	302.8	8.88E+00	8.94E+00	9.28E+00	4.86E+00	4.78E+00	4.63E+00
23	315.0	3.56E+01	3.57E+01	3.82E+01	4.32E+01	4.33E+01	4.63E+01

Table S3d. Computed vibrational frequencies and absolute values of $\partial a_i/\partial\boldsymbol{Q}$ derivatives for {Y_2-D_{5h}}.

N	$\boldsymbol{\nu}$ cm^{-1}	$\lvert\partial a_{1x}/\partial Q\rvert$ MHz Å^{-1}	$\lvert\partial a_{1y}/\partial Q\rvert$ MHz Å^{-1}	$\lvert\partial a_{1z}/\partial Q\rvert$ MHz Å^{-1}	$\lvert\partial a_{2x}/\partial Q\rvert$ MHz Å^{-1}	$\lvert\partial a_{2y}/\partial Q\rvert$ MHz Å^{-1}	$\lvert\partial a_{2z}/\partial Q\rvert$ MHz Å^{-1}
1	20.9	5.14E-01	5.26E-01	5.72E-01	3.43E-01	3.49E-01	3.63E-01
2	33.1	1.00E-03	1.53E-03	7.00E-03	9.28E-02	9.75E-02	1.15E-01
3	44.3	2.80E-01	2.85E-01	3.38E-01	8.41E-02	8.58E-02	9.74E-02
4	67.4	1.65E+01	1.68E+01	1.87E+01	6.42E+00	6.77E+00	6.65E+00
5	81.9	3.46E+00	3.13E+00	4.02E+00	8.76E+00	8.81E+00	1.06E+01
6	90.2	2.57E+01	2.56E+01	2.94E+01	2.36E+01	2.36E+01	2.75E+01
7	93.8	1.64E+01	1.64E+01	1.91E+01	1.46E+01	1.46E+01	1.71E+01
8	109.8	3.52E+00	3.54E+00	4.20E+00	4.74E+00	4.73E+00	5.34E+00
9	126.3	1.60E+00	1.59E+00	1.60E+00	1.52E+00	1.56E+00	1.53E+00
10	171.4	4.20E+00	4.18E+00	2.93E+00	2.45E+01	2.44E+01	2.63E+01
11	175.4	7.36E+01	7.37E+01	8.27E+01	4.78E+01	4.79E+01	5.67E+01
12	195.2	4.71E+01	4.71E+01	4.74E+01	5.58E+01	5.57E+01	5.74E+01
13	217.5	1.70E+00	1.78E+00	1.84E+00	4.38E+00	4.28E+00	4.32E+00
14	219.0	1.35E+01	1.35E+01	1.38E+01	1.04E+01	1.04E+01	1.04E+01
15	231.7	2.34E+01	2.33E+01	2.38E+01	2.75E+01	2.75E+01	2.83E+01
16	243.0	3.57E+01	3.56E+01	3.66E+01	3.25E+01	3.25E+01	3.33E+01
17	271.2	4.44E+01	4.44E+01	4.71E+01	3.81E+01	3.80E+01	4.08E+01
18	276.5	1.54E+01	1.55E+01	1.63E+01	1.02E+01	1.02E+01	1.11E+01
19	281.8	3.62E-01	1.46E-01	4.61E-01	9.51E+00	9.33E+00	9.89E+00
20	287.8	1.14E+01	1.14E+01	1.16E+01	3.01E+00	3.04E+00	2.86E+00
21	293.4	1.47E+00	1.47E+00	1.44E+00	4.06E+00	4.14E+00	4.48E+00
22	300.2	1.03E+01	1.01E+01	1.06E+01	5.21E+00	5.26E+00	5.79E+00
23	325.4	3.91E+01	3.87E+01	4.06E+01	4.07E+01	4.04E+01	4.26E+01

Vibrational modulation of g-tensor

Intensities in Fig. S17a are sums of squared derivatives of g-tensor components with respect to normal modes multiplied by a temperature factor:

$$\left(\left(\frac{\partial g_x}{\partial Q_k}\right)^2+\left(\frac{\partial g_y}{\partial Q_k}\right)^2+\left(\frac{\partial g_z}{\partial Q_k}\right)^2\right)\frac{\exp(h\nu_k/k_BT)}{(\exp(h\nu_k/k_BT)-1)^2}$$

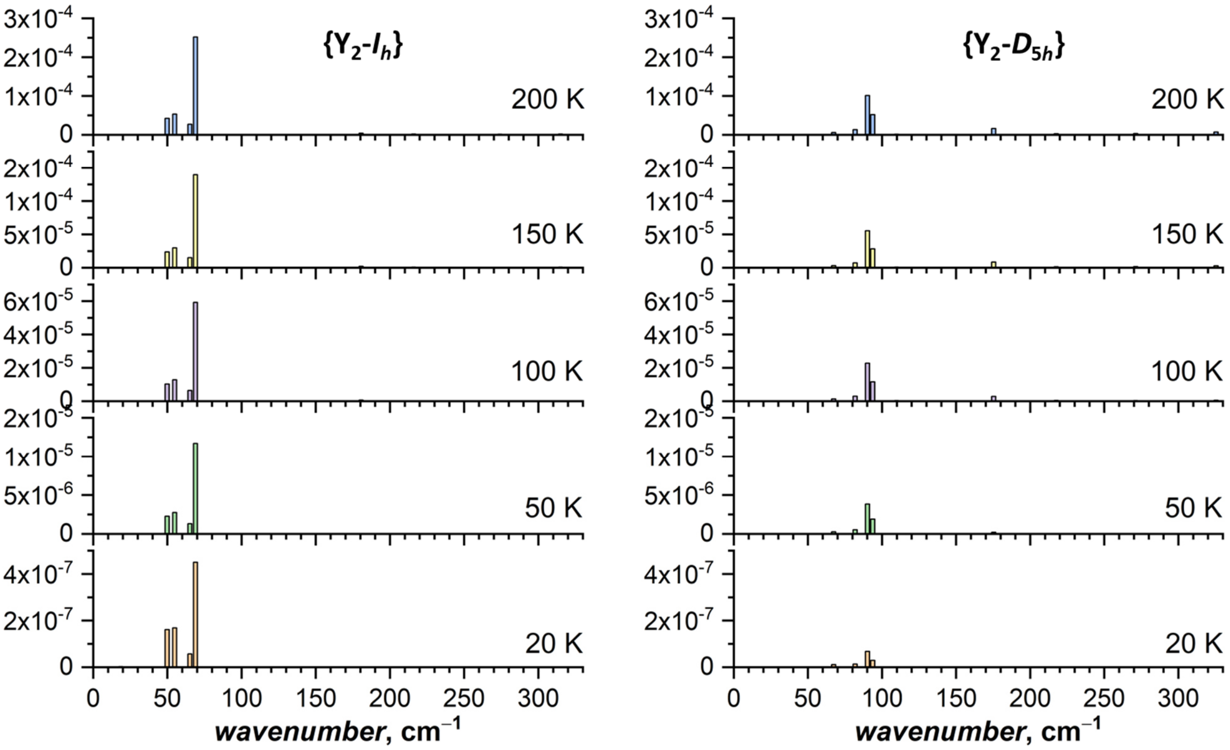

Figure S17a. The products of temperature factor and squared g-tensor derivatives for low-frequency vibrational modes of {Y_2-I_h} and {Y_2-D_{5h}} at different temperatures.

Vibrational modulation of hyperfine tensor

Intensities in Fig. S17b are sums of squared derivatives of A-tensor components with respect to normal modes multiplied by a temperature factor:

$$\left(\left(\frac{\partial a_{1x}}{\partial Q_k}\right)^2+\left(\frac{\partial a_{1y}}{\partial Q_k}\right)^2+\left(\frac{\partial a_{1z}}{\partial Q_k}\right)^2+\left(\frac{\partial a_{2x}}{\partial Q_k}\right)^2+\left(\frac{\partial a_{2y}}{\partial Q_k}\right)^2+\left(\frac{\partial a_{2z}}{\partial Q_k}\right)^2\right)\frac{\exp(h\nu_k/k_BT)}{(\exp(h\nu_k/k_BT)-1)^2}$$

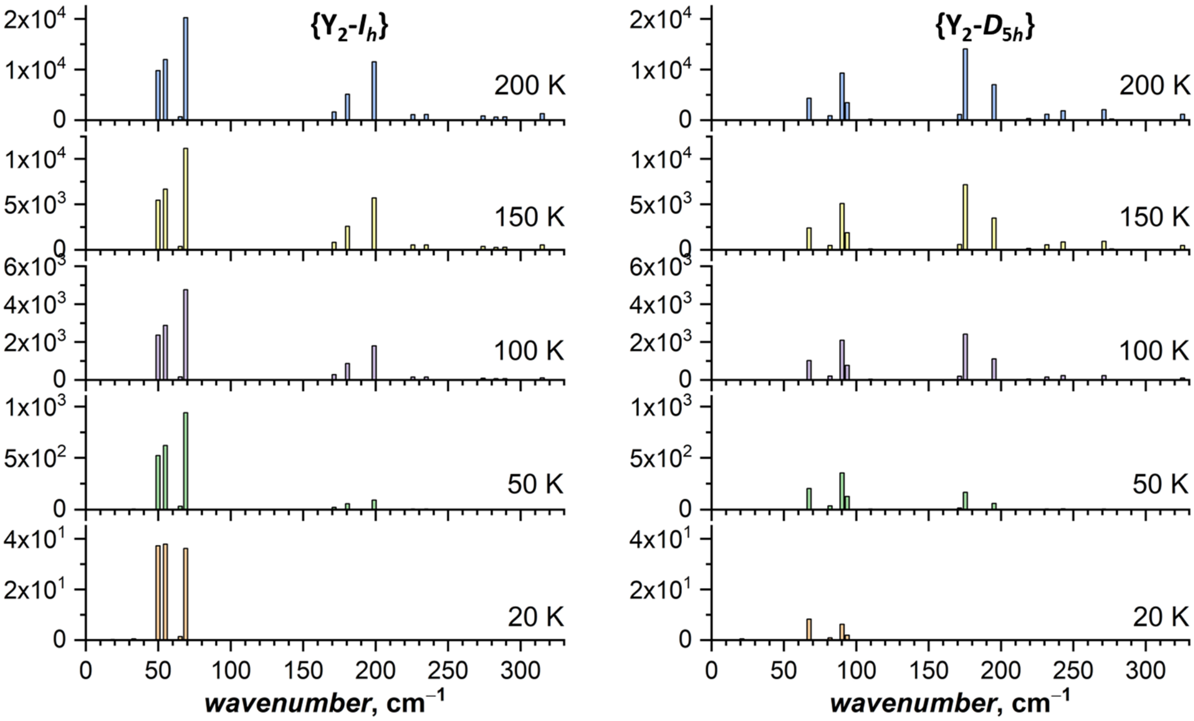

Figure S17b. The products of temperature factor and squared A-tensor derivatives for low-frequency vibrational modes of {Y_2-I_h} and {Y_2-D_{5h}} at different temperatures.

Table S4. T_1 and T_m times of Sc and Y dimetallofullerenes with single-electron M–M bond[a]

compound	T_1@$\mu_0 H$ µs@mTesla	*T*, K	T_m@$\mu_0 H$ µs@mTesla	*T*, K	conditions	ref
**Y_2@C_{79}N**	71153@345.1	5 K	23@345.1	5 K	CS_2, X-band	[1]
	4487@345.1	10 K	22@345.1	10 K		
	449@345.1	20 K	20@345.1	20 K		
	26@345.1	30 K	16@345.1	30 K		
**Y_2@C_{79}N⊂[4]CHBC**	75216@345.1	5 K	11@345.1	5 K	CS_2, X-band	[1]
	6724@345.1	10 K	10@345.1	10 K		
	893@345.1	20 K	9@345.1	20 K		
	41@345.1	30 K	9@345.1	30 K		
**CaY@*C_s*(6)-C_{82}**	8090@354	10 K	6.52@354	10 K	toluene, X-band	[2]
	1929@354	20 K	7.25@354	20 K		
	401@354	40 K	7.74@354	40 K		
	157.2@354	60 K	7.50@354	60 K		
	26@354	120 K	1.22@354	120 K		
	9.5@354	170 K	0.78@354	170 K		
**CaSc@*C_s*(6)-C_{82}**	41450@322.6	10 K	6.42@322.6	10 K	toluene, X-band	[3]
	7330@322.6	20 K	6.88@322.6	20 K		
	1740@322.6	40 K	7.43@322.6	40 K		
	725@322.6	60 K	6.96@322.6	60 K		
	90@322.6	120 K	0.76@322.6	120 K		
	20@322.6	160 K	0.46@322.6	170 K		
**Sc_2@I_h-C_{80}(CH_2Ph)**	32570@305.6	10 K	17.0@305.6	10 K	d_8-tol, X-band	[4]
	3570@305.6	20 K	16.7@305.6	20 K		
	296@305.6	40 K	13.5@305.6	40 K		
	76.5@305.6	60 K	11.6@305.6	60 K		
	26.0@305.6	120 K	2.2@305.6	120 K		
	$\boldsymbol{T_{1,z}/T_{1,xy}}$		$\boldsymbol{T_{m,xyz}/T_{m,mid}}$			
**Y_2@I_h-C_{80}(CH_2Ph)**	451/111	20 K	7.4/4.6	20 K	d_8-tol, W-band	t.w.
	21/8.6	50 K				
	6.9/3.1	79 K	0.73/0.33	79 K		
	2.9/1.5	105 K	0.27	105 K		
**Y_2@D_{5h}-C_{80}(CH_2Ph)**	781/311	20 K	8.3/5.7	20 K	d_8-tol, W-band	t.w.
	228/57	50 K	2.9/1.3	50 K		
	30/7.8	79 K	0.78/0.40	79 K		
	20/4.0	105 K	0.38	105 K		

T_1 and T_m the times are given for selected temperatures, including the lowest and the highest temperature reported in the papers.

Table S5. T_1 and T_m times of Gd^{III} and Eu^{II} metallofullerenes [a]

compound	T_1@μ_0H μs@mTesla	*T*, K	T_m@μ_0H μs@mTesla	*T*, K	conditions	ref
**YGd@I_h-C_{80}(CH_2Ph)**	10.3@3500	6 K	2.3@3500	6 K	d_8-tol, W-band	t.w.
	4.8@3500	10 K	1.7@3500	10 K		
	2.0@3500	20 K	0.7@3500	20 K		
**Gd_2@I_h-C_{80}(CH_2Ph)**	8.0@3350	6 K	1.7@3350	6 K	d_8-tol, W-band	t.w.
	4.9@3350	8 K	1.3@3350	8 K		
	4.0@3350	10 K	1.2@3350	10 K		
	2.5@3350	12 K	0.9@3350	12 K		
	1.8@3350	14 K				
**Gd_2@C_{79}N**	5.8@358.6	6 K	1.0@358.6	6 K	d_8-tol, X-band	[36]
	~0.8@358.6	14 K	~0.5@358.6	14 K		
**Gd@C_{82}**	1.7@34.5	5 K	0.56@34.5	5 K	d_8-tol, X-band	[44]
	0.59@34.5	10 K	0.24@34.5	10 K		
**Gd@C_{82}(Mrph)$_5$**	62@140	5 K	5.1@140	5 K	d_8-tol, X-band	[44]
	2.1@140	15 K	1.8@140	15 K		
	0.5@140	40 K				
**Gd@C_{82}(Mrph)$_7$**	72@140	5 K	4.4@140	5 K	d_8-tol, X-band	[44]
	2.6@140	15 K	1.6@140	15 K		
	1.0@140	20 K				
**Gd@C_{82}(Mrph)$_9$**	104@140	5 K	4.5@140	5 K	d_8-tol, X-band	[44]
	4.6@140	15 K	1.9@140	15 K		
	0.8@140	40 K				
**Eu@C_{74} (3)**	~45@194	3.3 K	~5@194	3.3 K	CS_2, X-band	[41]
	~2@194	15 K	~1.5@194	10 K		
	~55@8	3.3 K	~0.65@8	3.3 K		
	~2.5@8	15 K	~0.5@8	15 K		
			1.2@194	3.3 K	d_8-tol, X-band	[41]
**Eu@C_{80} (3')**	~50@116	3.7 K	6.0@116	3.7 K	CS_2, X-band	[41]
	~28@116	7 K	~3.5@116	6 K		
**Eu@C_{82} (2)**	~45@102	3.3 K	~2@102	3.3 K	CS_2, X-band	[41]
	~3.5@102	7 K	~1.7@102	5 K		
			1.7@269	3.3 K	d_8-tol, X-band	[41]
**Eu@C_{84} (1)**	~12@180	~4.4 K	~3.5@180	3.3 K	CS_2, X-band	[41]
	~3@180	8 K	~0.6@180	8 K		

T_1 and T_m times are given for the lowest and the highest temperature reported in the papers; in certain cases, the values measured at different fields are also shown; see Table S2 for spin parameters. When the numerical values are not listed in the original paper, but can be approximately read from the figures, they are listed with ~ prefix.

Rabi oscillations

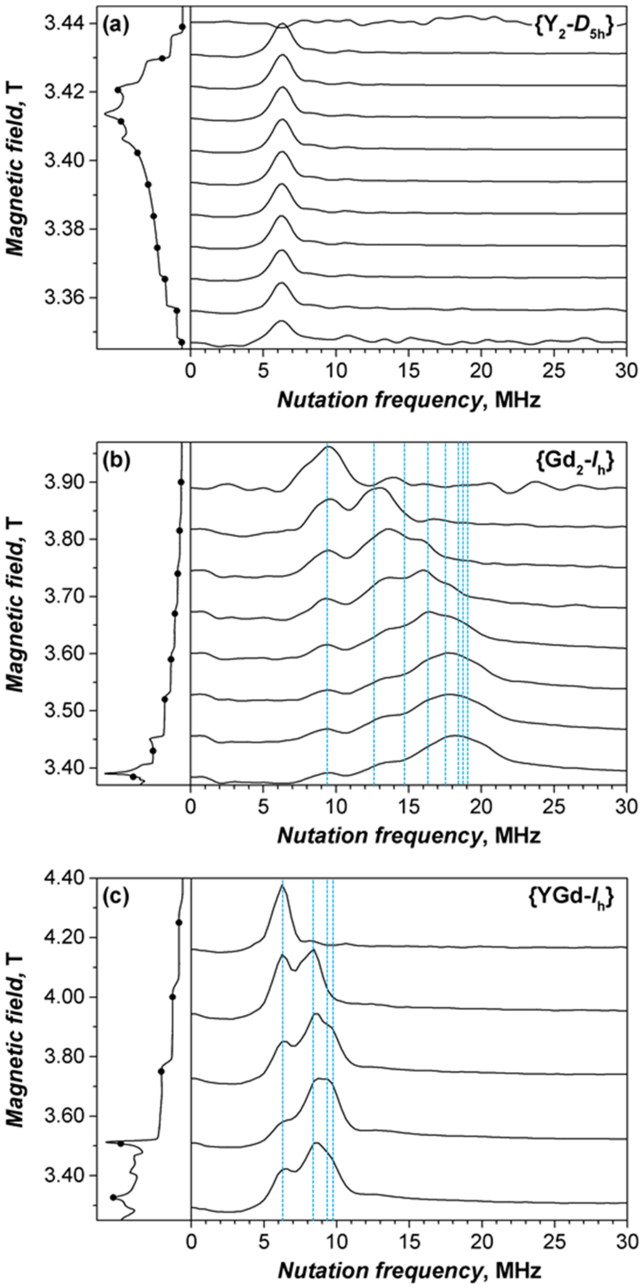

Figure S18. Normalized electron spin nutation spectra obtained by FT of Rabi oscillations at different field positions (dots in the ESE spectra, shown in the left panels) for (a) {Y_2-D_{5h}} at 20 K, with B_1 of 6.28 MHz, (b) {Gd_2-I_h} at 10 K, with B_1 of 2.12 MHz and (c) {YGd-I_h} at 6 K, with B_1 of 2.19 MHz. Vertical dashed lines mark theoretical positions of Rabi frequencies calculated as $\omega_{\mathrm{nut}} = \omega_1\sqrt{S(S+1) - m_S(m_S+1)}$. In (b) and (c), note a single peak for the spectra at the highest field, when only one $|m_S\rangle \rightarrow |m_S{+}1\rangle$ transition is excited, and increase of the number of (overlapping) peaks, when the excitation field is moved towards the center of the spectrum, where more and more individual $|m_S\rangle \rightarrow |m_S{+}1\rangle$ transitions are excited.

References

1. Liu, F.; Velkos, G.; Krylov, D. S.; Spree, L.; Zalibera, M.; Ray, R.; Samoylova, N. A.; Chen, C.-H.; Rosenkranz, M.; Schiemenz, S.; et al. Air-stable redox-active nanomagnets with lanthanide spins radical-bridged by a metal-metal bond. *Nat. Commun.* **2019**, *10*, 571.
2. Liu, F.; Krylov, D. S.; Spree, L.; Avdoshenko, S. M.; Samoylova, N. A.; Rosenkranz, M.; Kostanyan, A.; Greber, T.; Wolter, A. U. B.; Büchner, B.; et al. Single molecule magnet with an unpaired electron trapped between two lanthanide ions inside a fullerene. *Nat. Commun.* **2017**, *8*, 16098.
3. Cox, N.; Lubitz, W.; Savitsky, A. W-band ELDOR-detected NMR (EDNMR) spectroscopy as a versatile technique for the characterisation of transition metal–ligand interactions. *Mol. Phys.* **2013**, *111* (18-19), 2788-2808.
4. Nalepa, A.; Möbius, K.; Lubitz, W.; Savitsky, A. High-field ELDOR-detected NMR study of a nitroxide radical in disordered solids: Towards characterization of heterogeneity of microenvironments in spin-labeled systems. *J. Magn. Reson.* **2014**, *242*, 203-213.
5. Rapatskiy, L.; Cox, N.; Savitsky, A.; Ames, W. M.; Sander, J.; Nowaczyk, M. M.; Rögner, M.; Boussac, A.; Neese, F.; Messinger, J.; et al. Detection of the Water-Binding Sites of the Oxygen-Evolving Complex of Photosystem II Using W-Band ^{17}O Electron–Electron Double Resonance-Detected NMR Spectroscopy. *J. Am. Chem. Soc.* **2012**, *134* (40), 16619-16634.
6. Stoll, S.; Schweiger, A. EasySpin, a comprehensive software package for spectral simulation and analysis in EPR. *J. Magn. Reson.* **2006**, *178* (1), 42-55.
7. Neese, F. The ORCA program system. *WIREs Comput. Mol. Sci.* **2012**, *2* (1), 73-78.
8. Neese, F. Software update: the ORCA program system, version 4.0. *WIREs Comput. Mol. Sci.* **2018**, *8* (1), e1327.
9. Neese, F. Software update: The ORCA program system—Version 5.0. *WIREs Computational Molecular Science* **2022**, *12* (5), e1606.
10. Pantazis, D. A.; Chen, X.-Y.; Landis, C. R.; Neese, F. All-Electron Scalar Relativistic Basis Sets for Third-Row Transition Metal Atoms. *J. Chem. Theory Comput.* **2008**, *4* (6), 908-919.
11. Neese, F. Efficient and Accurate Approximations to the Molecular Spin-Orbit Coupling Operator and their use in Molecular g-Tensor Calculations. *J. Chem. Phys.* **2005**, *122*, 034107.
12. Hutter, J.; Iannuzzi, M.; Schiffmann, F.; VandeVondele, J. cp2k: atomistic simulations of condensed matter systems. *WIREs Comput. Mol. Sci.* **2014**, *4* (1), 15-25.
13. VandeVondele, J.; Krack, M.; Mohamed, F.; Parrinello, M.; Chassaing, T.; Hutter, J. Quickstep: Fast and Accurate Density Functional Calculations Using a Mixed Gaussian and Plane Waves Approach. *Comput. Phys. Commun.* **2005**, *167*, 103-128.
14. Humphrey, W.; Dalke, A.; Schulten, K. VMD - Visual Molecular Dynamics. *J. Molec. Graphics* **1996**, *14*, 33-38.
15. Wang, Y.; Velkos, G.; Israel, N. J.; Rosenkranz, M.; Büchner, B.; Liu, F.; Popov, A. A. Electrophilic Trifluoromethylation of Dimetallofullerene Anions en Route to Air-Stable Single-Molecule Magnets with High Blocking Temperature of Magnetization. *J. Am. Chem. Soc.* **2021**, *143* (43), 18139–18149.
16. Spree, L.; Liu, F.; Neu, V.; Rosenkranz, M.; Velkos, G.; Wang, Y.; Schiemenz, S.; Dreiser, J.; Gargiani, P.; Valvidares, M.; et al. Robust Single Molecule Magnet Monolayers on Graphene and Graphite with Magnetic Hysteresis up to 28 K. *Adv. Funct. Mater.* **2021**, *31*, 2105516.
17. Zuo, T.; Xu, L.; Beavers, C. M.; Olmstead, M. M.; Fu, W.; Crawford, T. D.; Balch, A. L.; Dorn, H. C. $M_2@C_{79}N$ (M = Y, Tb): Isolation and Characterization of Stable Endohedral Metallofullerenes Exhibiting M···M Bonding Interactions inside Aza[80]fullerene Cages. *J. Am. Chem. Soc.* **2008**, *130* (39), 12992-12997.
18. Feng, Y.; Wang, T.; Li, Y.; Li, J.; Wu, J.; Wu, B.; Jiang, L.; Wang, C. Steering Metallofullerene Electron Spin in Porous Metal–Organic Framework. *J. Am. Chem. Soc.* **2015**, *137* (47), 15055-15060.

19. Yan, Y.; Abella, L.; Sun, R.; Fang, Y.-H.; Roselló, Y.; Shen, Y.; Jin, M.; Rodríguez-Fortea, A.; de Graaf, C.; Meng, Q.; et al. Actinide-lanthanide single electron metal-metal bond formed in mixed-valence di-metallofullerenes. *Nat. Commun.* **2023**, *14* (1), 6637.
20. Gould, C. A.; McClain, K. R.; Reta, D.; Kragskow, J. G. C.; Marchiori, D. A.; Lachman, E.; Choi, E.-S.; Analytis, J. G.; Britt, R. D.; Chilton, N. F.; et al. Ultrahard magnetism from mixed-valence dilanthanide complexes with metal-metal bonding. *Science* **2022**, *375* (6577), 198-202.
21. Wedal, J. C.; Anderson-Sanchez, L. M.; Dumas, M. T.; Gould, C. A.; Beltrán-Leiva, M. J.; Celis-Barros, C.; Páez-Hernández, D.; Ziller, J. W.; Long, J. R.; Evans, W. J. Synthesis and Crystallographic Characterization of a Reduced Bimetallic Yttrium ansa-Metallocene Hydride Complex, [K(crypt)][(μ-CpAn)Y(μ-H)]2 (CpAn = Me2Si[C5H3(SiMe3)-3]2), with a 3.4 Å Yttrium–Yttrium Distance. *J. Am. Chem. Soc.* **2023**, *145* (19), 10730-10742.
22. Jena, R.; Benner, F.; Delano, F.; Holmes, D.; McCracken, J.; Demir, S.; Odom, A. L. A rare isocyanide derived from an unprecedented neutral yttrium(ii) bis(amide) complex. *Chem. Sci.* **2023**, *14* (16), 4257-4264.
23. Brennan, J. G.; Cloke, F. G. N.; Sameh, A. A.; Zalkin, A. Synthesis of bis(η-1,3,5-tri-t-butylbenzene) sandwich complexes of yttrium(0) and gadolinium(0); the X-ray crystal structure of the first authentic lanthanide(0) complex, [Gd(η-$Bu^{t3}C_6H_3)_2$]. *J. Chem. Soc., Chem. Commun.* **1987**, 10.1039/C39870001668 (21), 1668-1669.
24. MacDonald, M. R.; Ziller, J. W.; Evans, W. J. Synthesis of a Crystalline Molecular Complex of Y^{2+}, [(18-crown-6)K][$(C_5H_4SiMe_3)_3Y$]. *J. Am. Chem. Soc.* **2011**, *133* (40), 15914-15917.
25. Ariciu, A.-M.; Woen, D. H.; Huh, D. N.; Nodaraki, L. E.; Kostopoulos, A. K.; Goodwin, C. A. P.; Chilton, N. F.; McInnes, E. J. L.; Winpenny, R. E. P.; Evans, W. J.; et al. Engineering electronic structure to prolong relaxation times in molecular qubits by minimising orbital angular momentum. *Nat. Commun.* **2019**, *10* (1), 3330.
26. Corbey, J. F.; Woen, D. H.; Palumbo, C. T.; Fieser, M. E.; Ziller, J. W.; Furche, F.; Evans, W. J. Ligand Effects in the Synthesis of Ln^{2+} Complexes by Reduction of Tris(cyclopentadienyl) Precursors Including C–H Bond Activation of an Indenyl Anion. *Organometallics* **2015**, *34* (15), 3909-3921.
27. Angadol, M. A.; Woen, D. H.; Windorff, C. J.; Ziller, J. W.; Evans, W. J. tert-Butyl(cyclopentadienyl) Ligands Will Stabilize Nontraditional +2 Rare-Earth Metal Ions. *Organometallics* **2019**, *38* (5), 1151-1158.
28. Jenkins, T. F.; Woen, D. H.; Mohanam, L. N.; Ziller, J. W.; Furche, F.; Evans, W. J. Tetramethylcyclopentadienyl Ligands Allow Isolation of Ln(II) Ions across the Lanthanide Series in [K(2.2.2-cryptand)][$(C_5Me_4H)_3Ln$] Complexes. *Organometallics* **2018**, *37* (21), 3863-3873.
29. Jenkins, T. F.; Bekoe, S.; Ziller, J. W.; Furche, F.; Evans, W. J. Synthesis of a Heteroleptic Pentamethylcyclopentadienyl Yttrium(II) Complex, [K(2.2.2-Cryptand)]{$(C_5Me_5)_2Y^{II}[N(SiMe_3)_2]$}, and Its C–H Bond Activated Y(III) Derivative. *Organometallics* **2021**, *40* (23), 3917-3925.
30. Fang, M.; Lee, D. S.; Ziller, J. W.; Doedens, R. J.; Bates, J. E.; Furche, F.; Evans, W. J. Synthesis of the $(N_2)^{3-}$ Radical from Y^{2+} and Its Protonolysis Reactivity To Form $(N_2H_2)^{2-}$ via the $Y[N(SiMe_3)_2]_3/KC_8$ Reduction System. *J. Am. Chem. Soc.* **2011**, *133* (11), 3784-3787
31. Moehring, S. A.; Miehlich, M.; Hoerger, C. J.; Meyer, K.; Ziller, J. W.; Evans, W. J. A Room-Temperature Stable Y(II) Aryloxide: Using Steric Saturation to Kinetically Stabilize Y(II) Complexes. *Inorg. Chem.* **2020**, *59* (5), 3207-3214.
32. McClain, K. R.; Gould, C. A.; Marchiori, D. A.; Kwon, H.; Nguyen, T. T.; Rosenkoetter, K. E.; Kuzmina, D.; Tuna, F.; Britt, R. D.; Long, J. R.; et al. Divalent Lanthanide Metallocene Complexes with a Linear Coordination Geometry and Pronounced 6s–5d Orbital Mixing. *J. Am. Chem. Soc.* **2022**, *144* (48), 22193-22201.
33. Knight, L. B., Jr.; Kaup, J. G.; Petzoldt, B.; Ayyad, R.; Ghanty, T. K.; Davidson, E. R. Electron spin resonance studies of $^{45}Sc^{17}O$, $^{8}9Y^{17}O$, and $^{139}La^{17}O$ in rare gas matrices: Comparison with ab initio electronic structure and nuclear hyperfine calculations. *J. Chem. Phys.* **1999**, *110* (12), 5658-5669.

34. Fu, W.; Zhang, J.; Fuhrer, T.; Champion, H.; Furukawa, K.; Kato, T.; Mahaney, J. E.; Burke, B. G.; Williams, K. A.; Walker, K.; et al. $Gd_2@C_{79}N$: Isolation, Characterization, and Monoadduct Formation of a Very Stable Heterofullerene with a Magnetic Spin State of S = 15/2. *J. Am. Chem. Soc.* **2011**, *133*, 9741-9750.
35. Wang, X.; McKay, J. E.; Lama, B.; van Tol, J.; Li, T.; Kirkpatrick, K.; Gan, Z.; Hill, S.; Long, J. R.; Dorn, H. C. Gadolinium based endohedral metallofullerene $Gd_2@C_{79}N$ as a relaxation boosting agent for dissolution DNP at high fields. *Chem. Commun.* **2018**, *54* (19), 2425-2428.
36. Hu, Z.; Dong, B.-W.; Liu, Z.; Liu, J.-J.; Su, J.; Yu, C.; Xiong, J.; Shi, D.-E.; Wang, Y.; Wang, B.-W.; et al. Endohedral Metallofullerene as Molecular High Spin Qubit: Diverse Rabi Cycles in $Gd_2@C_{79}N$. *J. Am. Chem. Soc.* **2018**, *140*, 1123-1130.
37. Yamaguchi, T.; Nakatori, N.; Mitani, T.; Kikuchi, K.; Kodama, T.; Furukawa, K.; Kato, T. ESR spectra of Gd and Y di-metallofullerenes. In The 50th Fullerenes-Nanotubes-Graphene General Symposium, Tokyo, Japan; 2016.
38. Furukawa, K.; Okubo, S.; Kato, H.; Shinohara, H.; Kato, T. High-field/high-frequency ESR study of $Gd@C_{82}$-I. *J. Phys. Chem. A* **2003**, *107* (50), 10933-10937.
39. Huang, H.; Liu, Z.; Zhang, L.; Guo, X.; Cui, R.; Dong, J.; Chang, F.; Jiang, S.; Gao, S.; Sun, B. Paramagnetic properties adjustment for $Gd@C_{2v}(9)$-C_{82} by regioselective multi-amination. *Carbon* **2020**, *158*, 320-326.
40. B. Náfrádi; Á. Antal; Á. Pásztor; L. Forró; L.F. Kiss; T. Fehér; É. Kováts; Pekker, S.; Jánossy, A. Molecular and Spin Dynamics in the Paramagnetic Endohedral Fullerene $Gd_3N@C_{80}$. *J. Phys. Chem. Lett.* **2012**, *3*, 3291-3296.
41. Hu, Z.; Ullah, A.; Prima-Garcia, H.; Chin, S.-H.; Wang, Y.; Aragó, J.; Shi, Z.; Gaita-Ariño, A.; Coronado, E. Binding Sites, Vibrations and Spin-Lattice Relaxation Times in Europium(II)-Based Metallofullerene Spin Qubits. *Chem.-Eur. J.* **2021**, *27* (52), 13242-13248.
42. Matsuoka, H.; Ozawa, N.; Kodama, T.; Nishikawa, H.; Ikemoto, I.; Kikuchi, K.; Furukawa, K.; Sato, K.; Shiomi, D.; Takui, T.; et al. Multifrequency EPR study of metallofullerenes: $Eu@C_{82}$ and $Eu@C_{74}$. *J. Phys. Chem. B* **2004**, *108* (37), 13972-13976.
43. Bucher, K.; Mende, J.; Mehring, M.; Jansen, M. Isolation and spectroscopic characterization of $Eu@C_{72}$. *Fuller. Nanotub. Carbon Nanostruct.* **2007**, *15* (1), 29-42.
44. Liu, Z.; Huang, H.; Wang, Y.-X.; Dong, B.-W.; Sun, B.-Y.; Jiang, S.-D.; Gao, S. Amination of the $Gd@C_{82}$ endohedral fullerene: tunable substitution effect on quantum coherence behaviors. *Chem. Sci.* **2020**, *11* (39), 10737-10743.
